# Base editing-mediated exon-specific knockout of glutamine synthetase generates a high-productivity CHO cell line for antibody manufacturing

**DOI:** 10.1186/s40643-026-01150-0

**Published:** 2026-10-23

**Authors:** Tianting Pu, Chengcheng Wang, Hui Lou, Ye Mi, Saiyi Zhang, Siyu Liu, Zhihua Xiao, Qiyao Wang, Qin Liu, Yajing Liu

**Affiliations:** 1 State Key Laboratory of Bioreactor Engineering, East China University of Science and Technology, Shanghai, 200237 China; 2 Shanghai OPM Biosciences Co. Ltd, Shanghai, 201321 China

**Keywords:** Chinese hamster ovary (CHO) cell, Glutamine synthetase, Base editing, Premature termination codon

## Abstract

The Chinese hamster ovary (CHO) cell platform dominates recombinant protein production, with the glutamine synthetase (GS) system widely used for clonal selection. However, we observed variable performance between two commercial CHO lines (ATCC CHO-K1 and CHOZN^®^), which carry distinct intragenic GS deletions and differential residual transcript levels, raising the possibility that the premature termination codon (PTC) position contributes to this phenotypic divergence. To systematically assess the impact of the PTC position, we used base editing to introduce PTCs into each of the six coding exons of *Glul*. Among the resulting clonal lines, the exon 7-edited (E7) variant exhibited complete GS ablation and sustained high viability. In fed-batch production of an anti-PD-1 monoclonal antibody, E7 achieved higher titers and specific productivities than CHOZN^®^, with sustained viability. Our findings demonstrate that PTC location is a key determinant of GS knockout completeness and cellular phenotype, identifying exon 7 as a critical knockout target that offers a defined, exon-resolved strategy for accelerating CHO cell line development and improving recombinant protein titers.

**Graphical abstract:**

**Figure:**
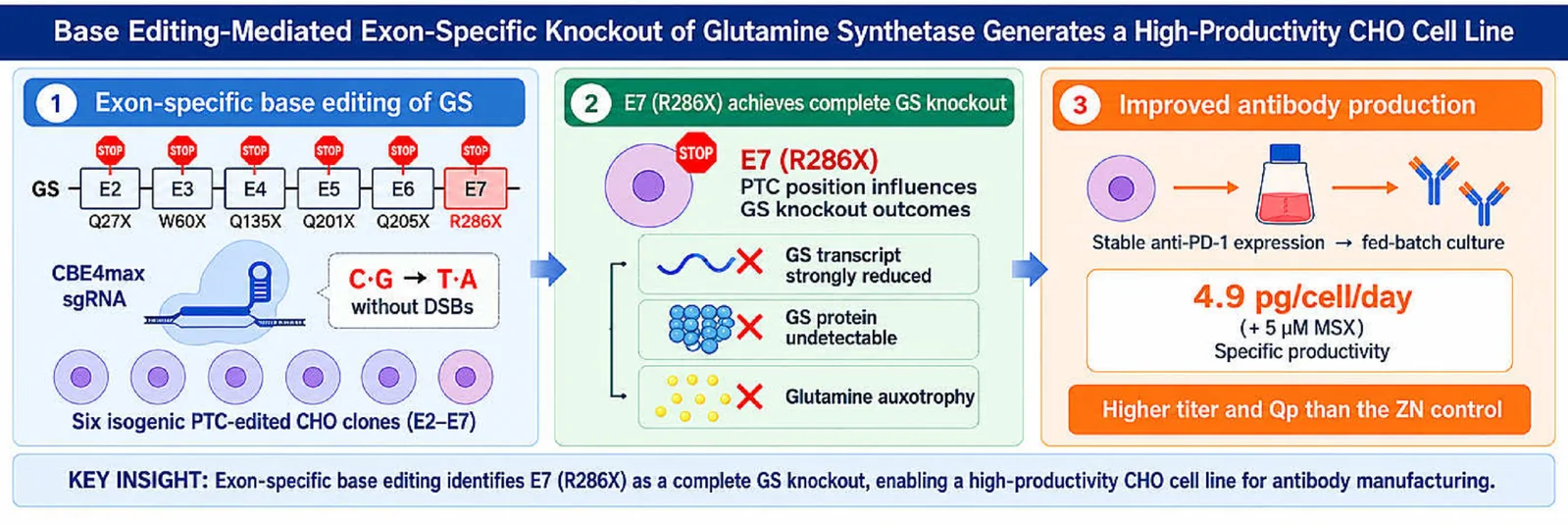

**Supplementary Information:**

The online version contains supplementary material available at https://doi.org/10.1186/s40643-026-01150-0.

## Introduction

CHO cells have become the predominant mammalian host cell system for the production of recombinant therapeutic proteins (RTPs), owing to their broad applicability in biopharmaceutical manufacturing (Walsh and Walsh 2022). Biologics, including monoclonal antibodies, fusion proteins, and other complex therapeutics, account for a substantial and growing share of new drug approvals, with monoclonal antibodies reaching a record number of approvals in 2024 (Martins et al. 2025; Wurm 2004). This widespread use is largely attributable to the ability of CHO cells to perform human-like post-translational modifications, which can support therapeutic efficacy while minimizing immunogenicity (Xu et al. 2011). CHO cells thrive in high-density suspension cultures, enabling scalable production in bioreactors exceeding 12,000 L, while their resistance to human pathogenic viruses and adaptability to serum-free, chemically defined media further enhance both manufacturing safety and regulatory compliance (Xu et al. 2023). Supported by decades of genomic characterization and genetic engineering, including dihydrofolate reductase (DHFR) (Urlaub and Chasin 1980) and glutamine synthetase (GS) (Liu et al. 2010) selection systems, CHO cells have therefore become a widely used platform for cell line development for RTP production (Walsh and Walsh 2022).

Efficient and reliable production of RTPs hinges on establishing stable cell lines capable of sustaining high-level, consistent expression. Currently, random transgene integration is the industry standard for stable cell line development for RTP production (Fan et al. 2012; Keating et al. 2021). To efficiently obtain productive cell lines, the DHFR-based methotrexate (MTX) amplification system has been adopted but often requires multiple rounds of selection and gene amplification, complicating workflows and increasing timelines. In recent years, the GS-based methionine sulfoximine (MSX) selection system has gained prominence in cell line development (CLD) screening (Bebbington et al. 1992; Chin et al. 2019; Xu et al. 2023). MSX-mediated selection enables the rapid establishment of stable cell lines (Brown et al. 1992), and GS-based selection additionally mitigates ammonia accumulation in culture media by converting glutamate and ammonia to glutamine, thereby enhancing cellular fitness and productivity (Noh et al. 2017). Owing to these advantages, the GS system has become a fundamental methodology for developing cell lines that are both robust and efficient.

The ATCC CHO-K1 cell line, established as the first subclone derived from parental CHO-ori cells, harbors a 26-bp deletion in exon 2 of the *Glul* gene (Xu et al. 2011). This frameshift mutation leads to premature GS translation termination and glutamine deficiency in standard medium. Building on this property, diverse gene-editing strategies have been applied to engineer endogenous *Glul* loci for selection purposes. In the CHOZN^®^ cells (Merck), zinc finger nuclease (ZFN) technology was used to generate a 10-bp deletion and a 1-bp substitution in *Glul* exon 6, conferring glutamine auxotrophy selection via frameshift-mediated inactivation (Fan et al. 2012; Liu et al. 2010). With the advancement of gene editing technology, the CRISPR/Cas system has become the mainstream gene editing tool for CHO cell engineering (Cong et al. 2013; Lee et al. 2015). More recently, CRISPR/Cpf1 was used to delete both the highly expressed *GS5* and low-expressed *GS1* genes in CHO-K1, reducing functional redundancy to improve selection stringency (Srila et al. 2023). However, these strategies share a common assumption: that any frameshift-derived PTC, regardless of its position within the gene, reliably abolishes GS function. This assumption has not been systematically tested, and it may help explain why CLD timelines and RTP titers remain highly variable across GS-optimized CHO platforms (Handlogten et al. 2018; Ladiwala et al. 2024; Yang et al. 2015).

This raises a fundamental question: does the location of a PTC within a gene determine its functional impact? PTCs near the translation initiation site, in particular, could be bypassed by ribosomes via leaky scanning or reinitiation, potentially yielding truncated GS proteins with residual enzymatic activity (Qiu et al. 2023), a possibility that conventional frameshift-based knockout strategies do not account for. We reasoned that truncation variants arising from different exons might therefore elicit divergent cellular effects, potentially explaining the variable recombinant protein yields observed across existing GS-knockout platforms. Defining how PTC position drives functional loss is thus essential for generating a complete and predictable GS-knockout (GS-KO) CHO cell line. Here, we address this question by systematically introducing PTCs into each exon of the GS gene and characterizing the resulting transcriptional and phenotypic outcomes.

To this end, we employed a cytosine base editor (CBE) system (Komor et al. 2016; Qiu et al. 2023; Rees and Liu 2018; Walton et al. 2020), which enables precise cytosine to thymine (C-to-T) conversion to generate PTCs without inducing double-strand breaks (DSBs). Using sgRNAs, we generated a series of isogenic CHO cell lines harboring distinct PTCs in each of the six coding exons of *Glul*: exon 2 (Q27X), exon 3 (W60X), exon 4 (Q135X), exon 5 (Q201X), exon 6 (Q205X) and exon 7 (R286X). Screening based on glutamine auxotrophy and MSX sensitivity identified the exon 7-edited line (E7) as exhibiting a complete GS-KO phenotype, whereas E6 retained partial GS activity or residual transcript stability, consistent with a position-dependent effect of PTC placement on knockout completeness. Functional characterization of E7 confirmed a complete functional GS-deficient phenotype, with no detectable GS protein and strongly depleted GS transcripts. Beyond establishing a complete GS-KO line, this exon-specific PTC engineering strategy offers a generalizable approach for achieving predictable gene inactivation in CHO cell line development (Ha et al. 2022).

## Materials and methods

### Plasmid construction

Single guide RNA (sgRNA) target sequences were designed to introduce PTCs into coding exons 2–7 of the Chinese hamster *Glul* gene using the CBE4max-SpRY cytosine base editor. Candidate sgRNAs were selected according to the sequence requirements and editing window of CBE4max-SpRY. For sgRNA plasmid construction, complementary oligonucleotides were annealed by mixing 10 µL of each oligonucleotide (10 µM), heating at 98 °C for 5 min, cooling to 37 °C at a rate of 0.1 °C/s, and holding at 37 °C for 5 min. The PGL3-U6-ACCG-BSD vector was digested with BsaI and purified. Annealed double-stranded oligonucleotides were ligated into the linearized vector in a 5 µL reaction containing 50 ng gel-purified vector, 1 µL annealed oligonucleotide product, 0.5 µL 10× T4 DNA ligase buffer, 0.5 µL T4 DNA ligase (TaKaRa, Beijing, China), and nuclease-free water. Ligation was performed at 16 °C for 1 h.

For comparison with conventional DSB-based genome editing approaches, ZFN and TALEN constructs targeting the *Glul* locus were also generated. The ZFN pair was synthesized based on the ZFN sequences previously reported for the generation of the CHOZN^®^ GS^−/−^ cell line (Fan et al. 2012), and the corresponding ZFN coding sequences were cloned into expression plasmids. TALEN constructs targeting *Glul* were designed and assembled using the modular assembly strategy described previously (Cermak et al. 2011). Seven TALEN pairs targeting *Glul* exon 6 were generated and subsequently evaluated for genome-editing activity. All sgRNA target sequences and the nucleotide sequences of the ZFN and TALEN constructs used in this study are provided in Table S1.

### Cell culture

ECACC CHO-K1 cells (European Collection of Authenticated Cell Cultures, UK; designated EK1), ATCC CHO-K1 GS^−/−^ cells (American Type Culture Collection, USA; designated AK1), and CHOZN^®^ GS^−/−^ cells (Merck, Darmstadt, Germany; designated ZN) were maintained in EX-CELL CD CHO Fusion medium (Merck) supplemented with 6 mM L-glutamine (Thermo Fisher Scientific, Darmstadt, Germany). Unless otherwise indicated, CHO cells were maintained in suspension at 37℃ with 8% CO₂ and shaking at 120 rpm. Cells were routinely seeded at 5 × 10⁵ viable cells/mL and passaged every 2–4 days. Viable cell density (VCD) and viability were determined by trypan-blue exclusion (Beyotime, Shanghai, China) using a Countstar automated cell-counting system (Alit Biotech, Shanghai, China).

### Transfection and determination of editing efficiency

ECACC CHO-K1 cells were seeded at 5 × 10^5^ cells/mL at 24 h before transfection. Cells were collected by centrifugation at 200 × g for 5 min, and the supernatant was discarded. For each electroporation, 1 × 10⁷ cells were resuspended in pre-cooled CD CHO Fusion electroporation buffer supplemented with 6 mM L-glutamine. For CRISPR/Cas9- and CBE-mediated editing, cells were electroporated with a total of 15 µg plasmid DNA, consisting of 10 µg Cas9 or CBE4max-SpRY expression plasmid and 5 µg of the corresponding sgRNA plasmid. For TALEN- and ZFN-mediated editing, cells were electroporated with 7.5 µg of each plasmid encoding the left and right nuclease monomers, respectively, for a total of 15 µg plasmid DNA. Electroporation was performed using a Bio-Rad electroporator (CA, USA) at 300 V and 950 µF. Following electroporation, cells were transferred to T-flasks for recovery and cultured under the conditions described above. Cells were harvested 48 h after transfection for subsequent analysis of genome-editing efficiency.

### Editing-efficiency analysis

Cells were harvested 48 h after transfection, and genomic DNA was extracted. Genomic regions encompassing the corresponding target sites were amplified by PCR using target-specific primers listed in Table S1. PCR products were subjected to Sanger sequencing (Biosune Inc., Shanghai, China). For CBE-mediated editing, Sanger sequencing chromatograms were analyzed using EditR to quantify nucleotide conversion frequencies at individual positions within the target region. Base-editing efficiency was defined as the frequency of the intended C-to-T conversion at the target nucleotide.

For DSB-mediated genome editing using CRISPR/Cas9, TALEN, or ZFN, Sanger sequencing chromatograms were analyzed using TIDE (Tracking of indels by DEcomposition) to quantify the overall frequency and size distribution of insertions and deletions (indels) at the target site. Editing efficiency was reported as the total indel frequency determined by TIDE. Indels with lengths corresponding to multiples of three nucleotides were classified as in-frame indels, and their cumulative frequency was calculated from the TIDE output.

### Isolation and genotyping of GS-edited clones

At 48 h after electroporation with the CBE4max-SpRY and sgRNA plasmids, blasticidin S (BSD; Beyotime Biotechnology, Shanghai, China) was added to the culture at a final concentration of 1 µg/mL. After 3 days of BSD selection, cells were collected by centrifugation and resuspended in fresh CD CHO Fusion medium supplemented with 6 mM L-glutamine. Once cell viability had recovered to ≥ 90%, single-cell cloning was performed by limiting dilution. Cells were seeded into 96-well plates at an average density of one cell per well in 200 µL EX-CELL CHO Cloning Medium (Merck) supplemented with 6 mM L-glutamine and cultured at 37 °C with 8% CO₂.

After approximately 15–20 days, wells showing sufficient cell growth were selected and sequentially expanded in 24-well and 6-well plates using CD CHO Fusion medium supplemented with 6 mM L-glutamine. A fraction of cells from each expanded clone was lysed using the One Step Mouse Genotyping Kit (Vazyme, Nanjing, China). Genomic regions encompassing the corresponding *Glul* target sites were amplified by PCR using primers listed in Table S1, and the PCR products were subjected to Sanger sequencing (Biosune Inc., Shanghai, China). Clones harboring the desired homozygous PTC-generating substitutions were selected for subsequent experiments. The confirmed clones were further expanded and adapted to suspension culture in SF125 medium (Thermo Fisher Scientific) at 37 °C with 8% CO₂ and shaking at 120 rpm.

### RT-qPCR

Total RNA was extracted from approximately 1 × 10⁶ cells using TRIzol reagent (Thermo Fisher Scientific) according to the manufacturer’s instructions. Briefly, cells were collected by centrifugation, lysed in 1 mL TRIzol reagent, and subjected to phase separation with chloroform. The aqueous phase was transferred to a new tube, and RNA was precipitated with isopropanol, washed with 75% ethanol, air-dried, and dissolved in RNase-free water. Genomic DNA removal and reverse transcription were performed using TransScript^®^ One-Step gDNA Removal and cDNA Synthesis SuperMix (TransGen Biotech, Beijing, China) according to the manufacturer’s instructions. Quantitative PCR was performed using PerfectStart^®^ Green qPCR SuperMix (TransGen Biotech) on an ABI real-time PCR system (Applied Biosystems, USA). The amplification program consisted of an initial denaturation at 95 °C for 5 min, followed by 40 cycles of 95 °C for 30 s, 60 °C for 30 s, and 72 °C for 30 s. For analysis of residual GS transcripts in the different GS-knockout cell lines, three primer pairs targeting regions within exons 2, 4 and 7 of the *Glul* transcripts were used to assess transcript abundance at different positions along the mRNA. Additional primer pairs were used to quantify the expression of *GS1*, *Upf1*, *Atf4*, *Ddit3*, *Ppp1r15a*, *Gls*, *Glud1*, *Got1*, *Got2*, *Slc1a5*, *Slc38a2*, *Bcl2*, *Bax*, *Mcl1*, *Casp3*, *Myc*, *Ccnd1*, *Mki67*, *Asns*, *Sesn2*, *Hspa5 and Xbp1s*. *Gapdh* was used as the internal reference gene. Relative gene-expression levels were calculated using the 2^⁻ΔΔCt^ method. All primer sequences used for RT-qPCR are listed in Table S1. Unless otherwise indicated, RT-qPCR experiments were performed with three biological replicates, and data are presented as mean ± SD.

### shRNA-mediated knockdown of UPF1

To investigate the involvement of nonsense-mediated mRNA decay (NMD) in the regulation of residual GS transcripts, four short hairpin RNAs (shRNAs) targeting *Upf1* were initially evaluated for knockdown efficiency. Cells were transfected with individual shRNA expression plasmids, and *Upf1* transcript levels were measured by RT-qPCR as described in Sect. 2.6. The shRNA exhibiting the highest knockdown efficiency (shRNA4) was selected for subsequent experiments. EK1, AK1, ZN, and the exon-specific GS-edited cell lines E2-E7 were transfected with shRNA4 targeting *Upf1*. Cells transfected with a non-targeting shRNA were used as controls. After 72 h, cells were collected, and *Upf1* and GS transcript levels were quantified by RT-qPCR as described in Sect.  2.6. All shRNA target sequences are provided in Table S1.

### Western blotting

Approximately 1 × 10^6^ cells were collected and lysed on ice for 10 min in pre-cooled lysis buffer supplemented with phenylmethylsulfonyl fluoride (PMSF). Cell lysates were centrifuged at 12,000 × g for 15 min at 4 °C, and the supernatants were collected. Total protein concentrations were determined using a BCA Protein Assay Kit (Epizyme, Shanghai, China). Equal amounts of total protein (20 µg per lane) were mixed with 5× loading buffer and denatured by boiling for 10 min. Proteins were separated on 12% polyacrylamide gels by electrophoresis at 80 V for 30 min followed by 120 V for 90 min and subsequently transferred onto PVDF membranes (Merck, Darmstadt, Germany) using rapid transfer buffer (Servicebio, Wuhan, China) at 400 mA for 90 min. Membranes were blocked with rapid blocking buffer (Epizyme) for 1 h at room temperature and then incubated overnight at 4 °C with primary antibodies against GS (rabbit monoclonal antibody, 1:2,000; Abcam, UK) and GAPDH (rabbit monoclonal antibody, 1:5,000; Huabio, Hangzhou, China). After three washes with PBST, membranes were incubated with HRP-conjugated anti-rabbit secondary antibody (1:5,000; Huabio) for 1 h at room temperature. Chemiluminescent signals were developed using ECL substrate (Epizyme) and detected using a chemiluminescence imaging system. Band intensities were quantified using ImageJ, and GS protein levels were normalized to GAPDH.

### Serial-passage growth assay under different glutamine concentrations

To evaluate functional GS deficiency and glutamine dependence, EK1, ZN, and the exon-specific GS-edited cell lines E2-E7 were subjected to serial-passage cultivation under different glutamine concentrations. Cells were cultured in VegaCHO medium (OPM Biosciences, Shanghai, China) supplemented with 0, 1, 2, or 6 mM L-glutamine. At the beginning of each passage, cells were inoculated at a viable cell density of 5 × 10⁵ cells/mL in a total culture volume of 30 mL. Viable cell density (VCD) and viability were measured daily using the Countstar system as described above. Cells were passaged every 3 days by re-inoculation into fresh medium containing the same concentration of L-glutamine at 5 × 10⁵ viable cells/mL. Serial cultivation was continued for five consecutive passages. Growth and viability profiles were generated from the daily measurements to compare the ability of the different GS-edited cell lines to sustain proliferation under glutamine-limited and glutamine-free conditions.

### Stable antibody-producing pool generation and fed-batch evaluation

To evaluate recombinant antibody production, EK1, ZN, and the exon-specific GS-edited cell lines E2-E7 were used to generate antibody-producing pools. Cells were seeded at 5 × 10⁵ viable cells/mL 24 h before electroporation. For each transfection, 1 × 10⁷ cells were electroporated with 40 µg of an all-in-one plasmid encoding the pembrolizumab heavy chain (HC) and light chain (LC) and GS cDNA using a Bio-Rad electroporator at 300 V and 950 µF. After 24 h of recovery, cells were collected by centrifugation and resuspended in glutamine-free medium containing 0 or 5 µM MSX. Additional EK1 and ZN pools were generated using 15 µM MSX. Selection was continued for 16 days. For fed-batch culture, EK1 and its GS-edited derivatives were inoculated into VegaCHO medium, whereas ZN cells were cultured in StarCHO medium, at 5 × 10⁵ viable cells/mL in a working volume of 30 mL. Cultures were fed on days 3, 5, 7, 9, 11, 13, and 15 with 5%, 5%, 6%, 6%, 5%, 5%, and 3% of the initial culture volume, respectively, using the corresponding feed medium, together with CDFS36 at 10% of the feed volume. Cultures were maintained for up to 16 days and harvested earlier if viability decreased below 60%. VCD and viability were monitored throughout culture. Glucose and lactate were measured using an M100 analyzer, and antibody titer was determined using a ForteBio system (Sartorius, Göttingen, Germany). Integral viable cell density (IVCD) was calculated using the trapezoidal method, and cell-specific productivity (Qp) was calculated by normalizing antibody production to IVCD.

###  RNA sequencing and transcriptomic analysis

For transcriptomic analysis, untransfected EK1, AK1, ZN, and E7 cells without exogenous antibody expression were collected during the exponential growth phase. Total RNA was extracted and subjected to RNA sequencing. RNA quality assessment, library preparation, high-throughput sequencing, raw-read quality control, alignment to the Chinese hamster reference genome, and gene-level quantification were performed using standard RNA-sequencing procedures. Gene-expression matrices were subsequently analyzed in R (v4.1.0). Principal component analysis (PCA) was performed using DESeq2 to evaluate global transcriptional similarities among the cell lines. Differential expression analysis was performed using DESeq2, with EK1 used as the reference for comparisons with AK1, ZN, and E7. Differentially expressed genes (DEGs) were defined by an absolute log₂ fold change > 1 and a Benjamini–Hochberg-adjusted *P* value < 0.05. Data manipulation was performed using dplyr (v1.0.7) and tidyr (v1.1.3), and data visualization was performed using ggplot2 (v3.3.5) and ggrepel (v0.9.1). Overlap among DEG sets from the AK1-versus-EK1, ZN-versus-EK1, and E7-versus-EK1 comparisons was visualized using VennDiagram (v1.7.3). Hierarchical clustering was performed to identify genes exhibiting similar expression patterns across the four cell lines. Gene Ontology (GO) enrichment analysis was performed using topGO (v2.40.0) to identify biological processes associated with the corresponding DEG sets. For transcriptome-wide assessment of sequence variants, single-nucleotide polymorphisms (SNPs) and small insertions/deletions (indels) were identified from the RNA-seq data using VarScan2 (v2.4.4). Variants were filtered using a base-quality score > 20, read depth ≥ 8, at least two supporting reads, and *P* < 0.01. Variant distributions in exonic, intronic, and untranslated regions (UTRs) were compared among EK1, AK1, ZN, and E7 cells, with multiple-testing correction performed using the Benjamini–Hochberg procedure. The raw RNA-seq data generated in this study have been deposited in the NCBI Sequence Read Archive under BioProject accession PRJNA1516528.

### Off-target and residual-vector analyses

Potential sgRNA-dependent off-target sites for the E7-targeting sgRNA were predicted using CAS-OFFinder against the Chinese hamster genome. 13 candidate off-target loci were selected for experimental validation, and the GS5 on-target locus was included as a reference. Genomic regions encompassing each candidate site were amplified by PCR using genomic DNA from E7 cells, and the PCR products were subjected to Sanger sequencing. Sequencing chromatograms were compared with the corresponding reference sequences to identify potential nucleotide substitutions at the predicted off-target sites. Primer sequences used for off-target validation are provided in Table S1.

Because of the high sequence similarity between the canonical *Glul* locus on chromosome 5 (*GS5*) and its homologous *GS1* locus on chromosome 1, and because the E7 sgRNA target sequence is conserved between the two loci, *GS1* was analyzed separately. The *GS1* target region was amplified from genomic DNA of independent E7 clones and subjected to Sanger sequencing to determine its editing status.

In addition to targeted off-target analysis, transcriptome-wide SNP and indel profiles were evaluated using the RNA-seq data as described in Sect. 2.11 to assess whether CBE-mediated editing was associated with an increased overall sequence-variant burden in expressed genomic regions. To assess the persistence of plasmid-derived sequences after establishment of the edited cell lines, genomic DNA from E2-E7 cells was subjected to PCR using primer pairs targeting the CBE4max-SpRY and sgRNA-BSD plasmid sequences. The corresponding plasmids and untransfected cells were used as positive and negative controls, respectively. PCR products were analyzed by agarose gel electrophoresis to determine whether residual editor-derived sequences were detectable in the established cell lines. Primer sequences are provided in Table S1.

### Statistical analysis of experimental data and visualization

**Fig. 1:**
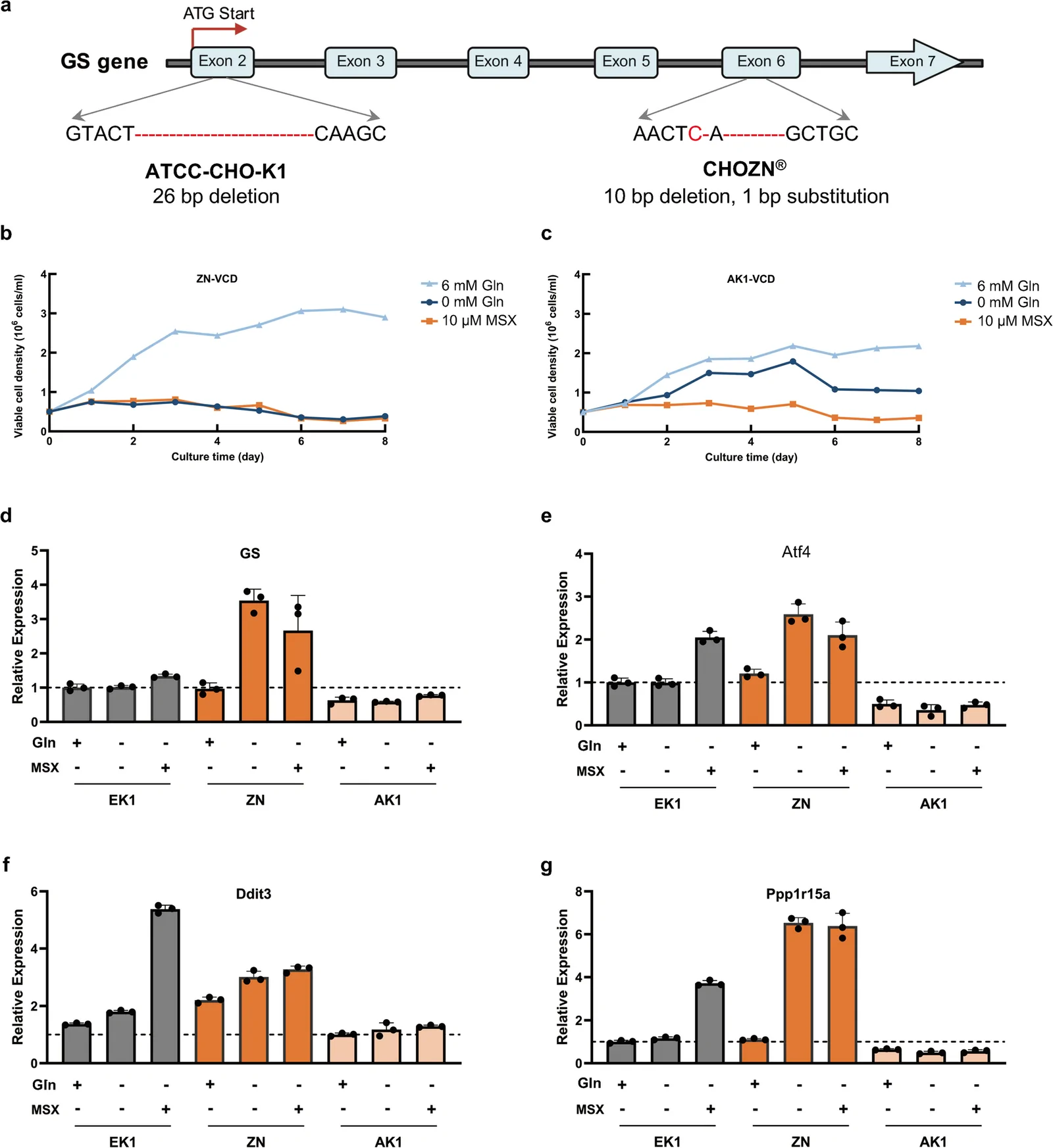
Validation of glutamine auxotrophy and stress-response signatures in commercial GS-knockout CHO cell lines. **a** Schematic of coding exons 2–7 of the Chinese hamster *Glul* gene. The ATG start codon is located in exon 2. Sequence alignments show the 26-bp deletion in exon 2 of ATCC CHO-K1 GS-knockout cells (AK1) and the 10-bp deletion plus 1-bp substitution in exon 6 of CHOZN^®^ cells (ZN). **b**,** c** Viable cell density profiles of ZN (**b**) and AK1 (**c**) cells cultured in medium containing 6 mM glutamine without MSX, glutamine-free medium without MSX, or glutamine-free medium supplemented with 10 µM MSX. Data in (**b**,** c**) were obtained from a single independent 30-mL culture for each cell line/condition using a standardized industrial CDMO platform. Analytical measurements were performed in technical triplicate, but these were not treated as biological replicates. (**d**-**g**) RT-qPCR analysis of *Glul* (GS) (**d**), *Atf4* (**e**), *Ddit3* (**f**), and *Ppp1r15a* (**g**) transcript levels in EK1, ZN, and AK1 cells under the same three culture conditions. *Gapdh* was used as the internal reference. Data in (d-g) are mean ± SD from three independent biological replicates (*n* = 3)

Statistical analyses were performed using GraphPad Prism 11 (GraphPad Software, San Diego, USA). Unless otherwise indicated, quantitative data are presented as mean ± SD from three independent biological replicates (*n* = 3). The culture experiments shown in Figs. 1b and c, 3 and 4, Fig. S1, Fig. S3, and Fig. S5 were performed as single-run experiments in a standardized industrial CDMO platform and therefore do not represent biological triplicates. For comparisons among multiple experimental groups, statistical significance was assessed using one-way analysis of variance (ANOVA) followed by Dunnett’s multiple-comparisons test, with the indicated control group used as the reference. Exact statistical tests and reference groups are specified in the corresponding figure legends. A *P* < 0.05 was considered statistically significant. For transcriptomic analyses, multiple-testing correction was performed using the Benjamini–Hochberg method as described in Sect.  2.11, and an adjusted *P* < 0.05 was considered statistically significant where applicable.

**Fig. 2:**
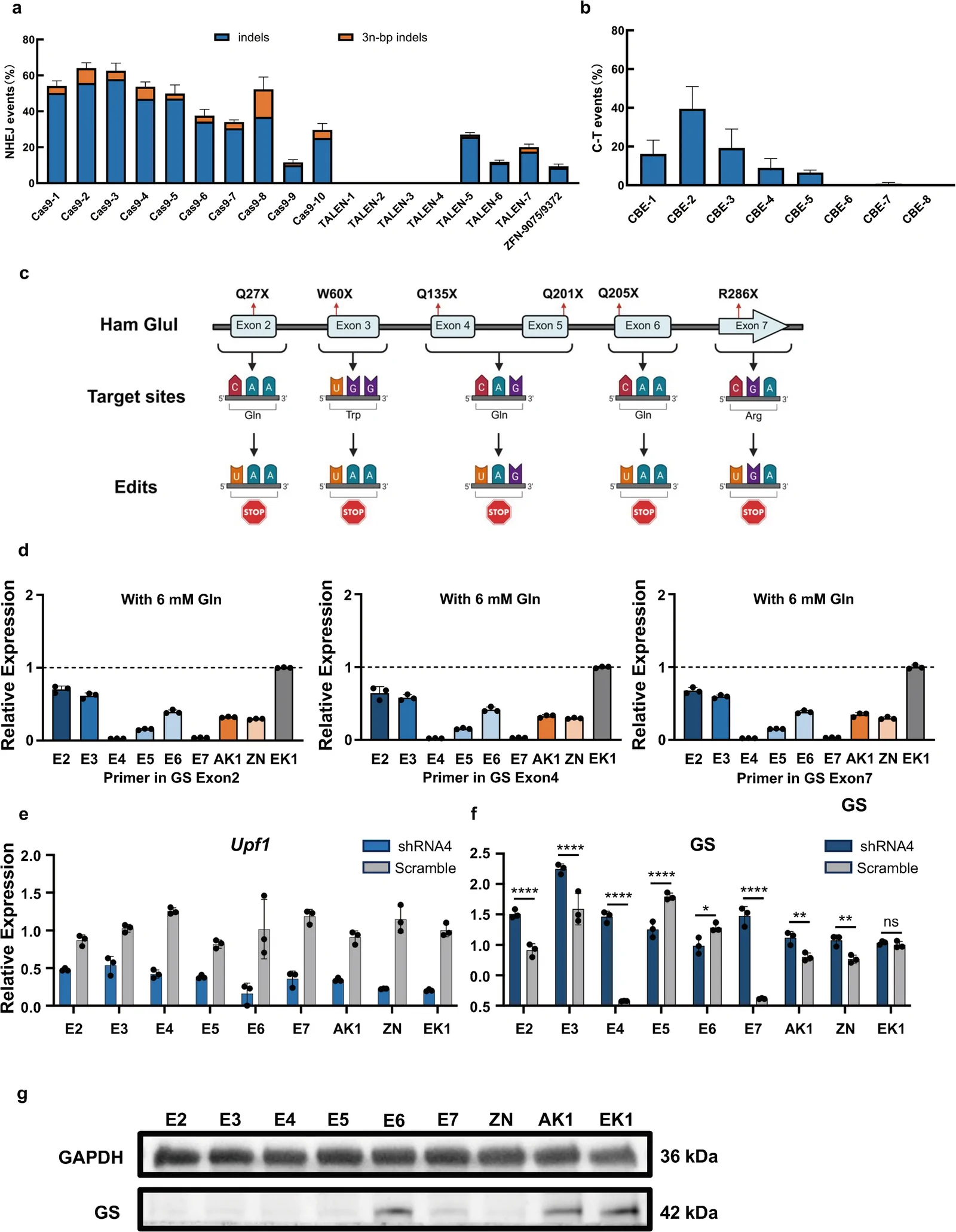
Exon-specific PTC placement determines residual GS expression and differential susceptibility to UPF1-dependent nonsense-mediated mRNA decay. **a** Frequencies of non-homologous end-joining (NHEJ) events generated at *Glul* exon 6 by 10 CRISPR/Cas9 sgRNAs, seven TALEN pairs, and one ZFN pair. Orange segments denote in-frame indels of 3n bp. **b** C-to-T editing efficiencies of eight CBE sgRNAs targeting *Glul* exon 6; CBE-2 produced the highest editing efficiency (39.7%). **c** Schematic of CBE-mediated PTC introduction into coding exons 2–7, generating Q27X, W60X, Q135X, Q201X, Q205X, and R286X. **d** Residual *Glul* transcript levels in E2-E7, AK1, ZN, and EK1 cells measured by RT-qPCR with primer pairs located in exons 2, 4, and 7. **e** *Upf1* transcript levels after transfection with UPF1-targeting shRNA4 or a non-targeting scramble control. **f** Residual *Glul* transcript levels after UPF1 depletion. Statistical significance in **f** was assessed by two-way ANOVA followed by Šídák’s multiple-comparisons test, comparing shRNA4 with the corresponding scramble control within each cell line. ns, not significant; *P < 0.05, **P < 0.01, ***P < 0.001, and ****P < 0.0001. Data are mean ± SD from three independent biological replicates (*n* = 3). **g** Western blot analysis of GS protein in E2-E7, ZN, AK1, and EK1 cells; GAPDH served as the loading control

**Fig. 3:**
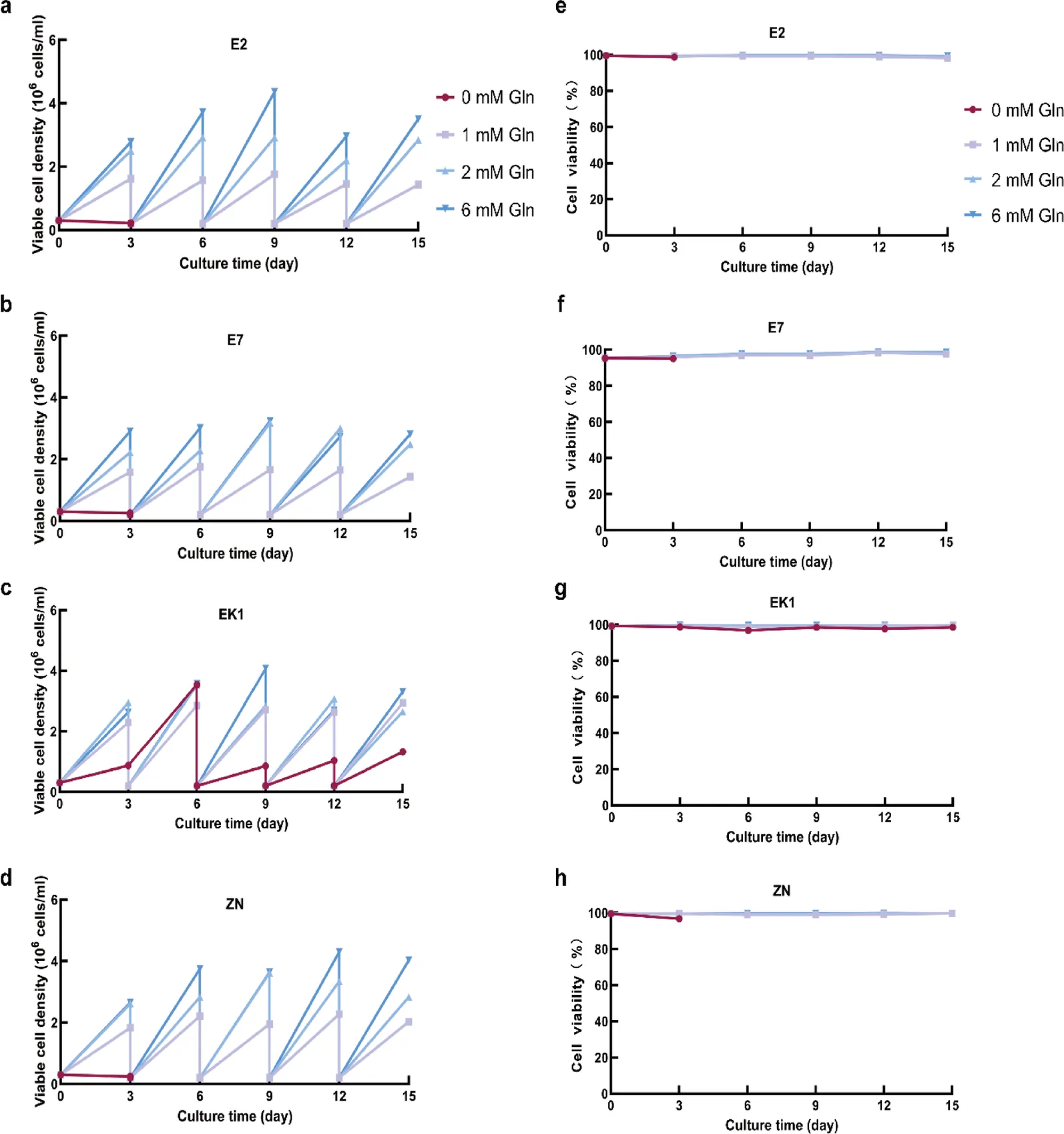
Serial-passage growth and viability under different glutamine concentrations. **a**-**d** Viable cell density profiles of E2 (**a**), E7 (**b**), EK1 (**c**), and ZN (**d**) cells cultured in medium containing 0, 1, 2, or 6 mM glutamine. Cells were inoculated at 5 × 10⁵ viable cells/mL and re-inoculated at the same density every 3 days for five consecutive passages. **e**-**h** Cell viability profiles corresponding to (**a**-**d**). Data were obtained from a single independent 30-mL culture for each cell line condition using a standardized industrial CDMO platform. Analytical measurements were performed in technical triplicate, but these were not treated as biological replicates

**Fig. 4:**
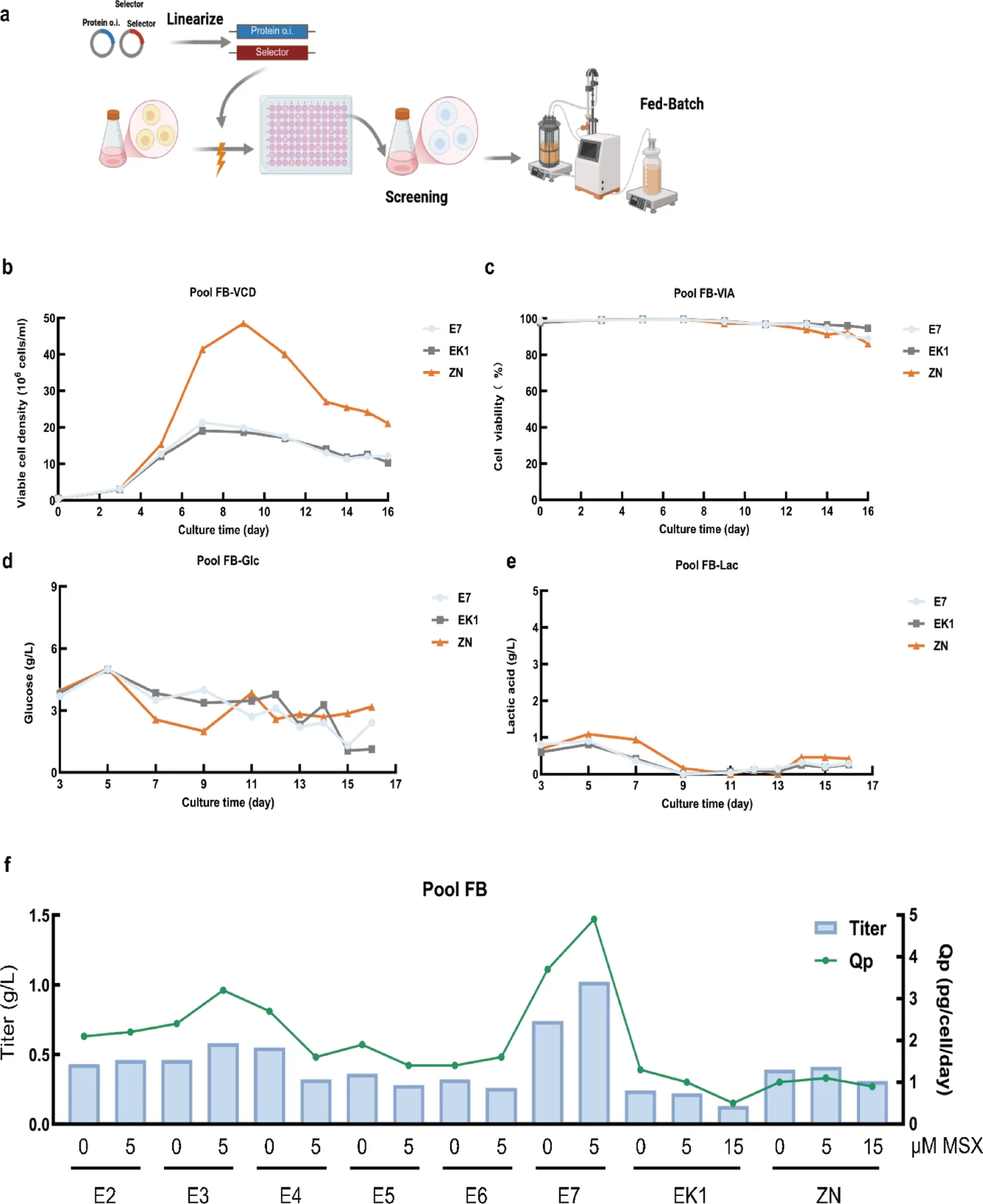
Fed‑batch production performance of exon‑specific GS‑knockout cell lines. **a** Workflow for generating antibody-producing cells, including linearization of an all-in-one vector encoding the antibody heavy chain, light chain, and GS selectable marker; electroporation; selection and screening; expansion; and fed-batch evaluation. **b**-**e** Viable cell density (**b**), cell viability (**c**), glucose concentration (**d**), and lactate concentration (**e**) of E7, EK1, and ZN stable pools established without MSX during the 16-day fed-batch culture. **f** Final antibody titer (bars, left y-axis) and cell-specific productivity (Qp; green line, right y-axis) of E2-E7, EK1, and ZN stable pools. The MSX concentration used during stable-pool selection is indicated below each bar. E7 achieved the highest titer and Qp, reaching 1.02 g/L and 4.9 pg/cell/day, respectively, after selection with 5 µM MSX. Data in **b**-**f** were obtained from a single independent 30-mL fed-batch culture for each cell line/MSX condition using a standardized industrial CDMO platform. Analytical measurements were performed in technical triplicate, but these were not treated as biological replicates

## Results

### Distinct GS-knockout genotypes drive divergent stress responses and glutamine dependence in commercial CHO cell lines

The GS-based selection system relies on two widely used GS-deficient host platforms: ATCC CHO-K1 (AK1) and Merck CHOZN^®^ (ZN) (Fig. 1a). Although both are nominally GS-deficient, the two lines responded differently to glutamine withdrawal: ZN cells were markedly more sensitive to glutamine deprivation (Fig. 1b; Fig. S1a), whereas AK1 cells sustained relatively active proliferation even under glutamine-free conditions (Fig. 1c; Fig. S1b, c). Under MSX selection, both lines proliferated more slowly (Fig. 1b, c). Sequencing of the *Glul* gene revealed that AK1 carried a 26-bp deletion in exon 2, whereas ZN harbored a compound mutation in exon 6 (a 10-bp deletion combined with a 1-bp substitution) (Fig. 1a); as expected, both mutations introduced frameshifts within the coding region. Consistent with these frameshifts, GS protein was undetectable in ZN and only faintly detectable in AK1 by Western blot (Fig. S1d).

Given that both lines nonetheless retain a frameshifted GS allele, we next asked whether the corresponding transcript is also eliminated, or merely rendered non-functional at the protein level. Surprisingly, qPCR revealed substantial residual GS mRNA in both AK1 and ZN (Fig. 1d), indicating that the frameshift mutations do not abolish transcription and that the apparent loss of GS protein reflects a post-transcriptional block rather than transcript elimination. The relationship between residual GS transcripts and GS protein levels also differed depending on glutamine availability. Under glutamine-free conditions, residual GS transcript levels in both AK1 and ZN remained comparable to or lower than those in EK1, the GS-competent wild-type control (Fig. 1d). Relative to their respective glutamine-replete conditions, however, glutamine withdrawal markedly increased residual GS transcript levels in ZN, whereas AK1 showed little change. This divergence suggests that glutamine deprivation triggers a compensatory transcriptional response specifically in ZN; yet because GS protein levels remained unchanged despite this transcriptional increase (Fig. S1d), the additional transcript evidently cannot be productively translated into functional enzyme. Thus, residual GS transcript abundance should not be interpreted as a direct surrogate for residual GS protein expression after PTC-generating mutations.

This raised the possibility that the accumulated non‑productive GS transcripts are not merely inert by‑products but may themselves impose a cellular burden, for example, through translational stress or direct RNA toxicity. Such a burden could contribute to the heightened glutamine sensitivity of ZN independently of GS protein deficiency. To test this, we profiled residual GS transcripts alongside markers of the integrated stress response (ISR), endoplasmic reticulum (ER) stress, and the unfolded protein response (UPR), including *Atf4*, *Ddit3*, and *Ppp1r15a* (Harding et al. 2003; Pakos-Zebrucka et al. 2016), in EK1, ZN, and AK1, with and without glutamine and MSX (Fig. 1e-g). In EK1, glutamine withdrawal modestly up-regulated endogenous GS, consistent with the expected compensatory response to glutamine shortage. ZN exhibited a similar but substantially amplified response, co-inducing residual GS together with *Atf4*, *Ddit3*, and *Ppp1r15a* under deprivation. AK1, by contrast, was essentially unresponsive: neither GS transcript nor any stress marker changed appreciably with glutamine status. Thus, although AK1 and ZN harbor comparable levels of residual GS transcripts, they differ markedly in their engagement of cellular stress pathways. This difference, rather than residual GS transcript abundance itself, may therefore contribute to their divergent responses to glutamine deprivation.

### PTC position determines residual GS expression

To resolve how distinct GS-knockout (GS-KO) patterns affect CHO production performance, and to establish robust GS-KO lines, we first benchmarked the editing efficiency of four genome-editing platforms—ZFN, TALEN, CRISPR/Cas9, and CBE—at a conserved locus in CHO cells (Fig. S2a). Because ZFN activity depends on the design and optimization of zinc-finger proteins (ZFPs), which can be labor-intensive, we used previously validated ZFN pairs developed for CHOZN^®^ cell line construction. In parallel, we designed 10 sgRNAs and 7 TALEN pairs targeting exon 6 of *Glul* (Fig. S2a). CRISPR/Cas9 achieved indel frequencies of up to 64%, three of seven TALEN pairs showed detectable activity (maximum 25.7%), and the ZFN pair reached 9.4% (Fig. 2a). Notably, all of these DSB-dependent strategies generated a fraction of in-frame indels (0.6–15.3%)—insertions or deletions in multiples of three base pairs at the cleavage site—that may fail to disrupt the GS reading frame (Fig. 2a). For comparison, we designed 8 sgRNAs targeting exon 6 for CBE-mediated editing (Fig. S2a). Five produced efficient C-to-T conversion, converting codons for glutamine (CAA), tryptophan (TGG), and arginine (CGA) into PTCs; the highest efficiency (39.7%) was achieved with sgRNA-2 (Fig. 2b). Because CBE installs PTCs without generating DSBs, the measured base-editing efficiency directly reflects the frequency of the intended PTC-generating edit. Thus, the ability of CBE to introduce site-specific PTCs offers a reliable strategy for the subsequent, exon-resolved analysis of how GS-knockout pattern relates to glutamine sensitivity in CHO cells.

We next designed sgRNAs to introduce PTCs across the six coding exons of the GS gene. After transient transfection and monoclonal screening, we obtained six PTC- edited monoclonal lines carrying premature termination mutations at distinct exons: exon 2 (Q27X), exon 3 (W60X), exon 4 (Q135X), exon 5 (Q201X), exon 6 (Q205X), and exon 7 (R286X) (Fig. 2c, Fig. S2b). Relative to EK1 under standard glutamine-replete culture, residual GS transcripts were approximately 0.65-fold in both ZN and AK1, and 0.7-, 0.6-, 0.03-, 0.15-, 0.39-, and 0.04-fold for the exon 2 through exon 7-edited lines, respectively (Fig. 2d). The same pattern was obtained with qPCR primers targeting the upstream, middle, and downstream regions of the GS transcript, confirming the robustness of the quantification (Fig. 2d).

To determine whether the differential abundance of residual GS transcripts was attributable to NMD, we depleted UPF1, a core component of the NMD pathway, using shRNA. Among the four candidate shRNAs, shRNA4 produced the greatest reduction in UPF1 at both the mRNA and protein levels and was therefore selected for subsequent experiments (Fig. S2c, d). RT-qPCR confirmed that *Upf1* transcript levels were reduced by approximately 45.0–84.1% across the nine cell lines relative to the corresponding scramble controls (Fig. 2e). UPF1 depletion markedly restored GS transcript abundance in E4 and E7, increasing GS mRNA levels by approximately 19.2- and 12.8-fold, respectively (Fig. 2f). More moderate increases were detected in E2 and E3 (1.65- and 1.42-fold, respectively). In contrast, GS transcript levels were not rescued in E5 or E6. Among the control cell lines, AK1 and ZN showed modest increases of approximately 1.40-fold, whereas no significant change was detected in EK1. Thus, the response to UPF1 depletion was not uniform across the cell lines. Notably, the exceptionally strong restoration of GS transcripts in E4 and E7, which substantially exceeded the changes observed in the control cell lines, provides functional evidence that UPF1-dependent NMD is a major contributor to the pronounced depletion of their PTC-containing GS transcripts.

At the protein level, GS was undetectable in all PTC-edited lines except E6 (Fig. 2g), despite the presence of substantial residual GS transcripts in several lines, particularly E2 and E3. This transcript–protein discordance indicates that residual PTC-containing mRNA does not necessarily result in detectable GS protein. Collectively, these results demonstrate that PTC position influences both transcript fate and functional GS expression, but transcript abundance alone is insufficient to predict knockout completeness.

### PTC position determines functional GS knockout and glutamine auxotrophy in CHO cells

To characterize the functional consequence of the exon-specific PTC insertion, we performed serial-passage cultivation across a glutamine gradient (0, 1, 2, and 6 mM), seeding cells at 5 × 10^5^ cells/mL in 30 mL of medium at each passage. Consistent with the immunoblot results (Fig. 2g), most edited lines showed complete functional loss of GS. Only the EK1 parental line and the E6 line proliferated in glutamine-free medium; all other GS-edited lines displayed a stringent GS-deficient phenotype with concentration-dependent growth. These knockout lines displayed progressively slower growth and declining viability in low-glutamine conditions (1 and 2 mM), consistent with progressive depletion of extracellular glutamine over serial passages, whereas 6 mM glutamine supported robust proliferation and high viability in all lines (Fig. 3; Fig. S3).

Given the high sequence identity between *GS5* and its paralog *GS1* (located on chromosome 1), *GS5*-targeting sgRNAs could potentially cross-edit *GS1*. This raised the possibility that the observed panel phenotypes, and particularly the superior performance of E7, might arise from *GS1* disruption rather than from the position of the PTC within *GS5*. We therefore interrogated *GS1* genotypes across the panel and assessed their functional contribution to the knockout phenotype. Sanger sequencing of the homologous *GS1* targeting region revealed that *GS1* remained intact in the commercial AK1 and ZN lines, as well as in our E2-E6 lines, with editing confined exclusively to *GS5*. The sole exception was E7: the exon 7 (R286X) sgRNA shares complete homology with the corresponding *GS1* sequence and consequently also edited *GS1* in this line (Fig. S4a). Among three independent E7 clones carrying the identical homozygous R286X mutation in *GS5*, *GS1* was homozygously edited in E7‑1, heterozygously edited in E7‑2, and unedited in E7‑3 (Fig. S4a). Despite their divergent *GS1* genotypes, the E7 line exhibited complete glutamine auxotrophy and maintained high viability during serial passage (Fig. 3b, f). Importantly, GS protein was undetectable in all three independently derived E7 clones, including E7-3, which retained an unedited *GS1* locus (Fig. S4e). Critically, clone E7-3, which retained an unedited *GS1* locus, exhibited growth and viability profiles comparable to those of E7-1 and E7-2 under both glutamine-replete and glutamine-free conditions (Fig. S4f). Consistent with these findings, *GS1* transcript abundance was examined across E2-E7, ZN, AK1 and EK1 cells and showed no significant difference from the EK1 control in most lines, indicating an absence of compensatory GS1 upregulation following GS5 disruption (Fig. S4b).

Finally, to assess the reproducibility of the editing outcomes across independently derived clones, we generated additional E2 and E7 clones. Sanger sequencing confirmed the intended Q27X and R286X substitutions in the independent clones (Fig. S4c, d), and GS protein was undetectable in all tested E2 and E7 clones (Fig. S4e). Together with the GS1 genotyping and expression results (Fig. S4a, b), these findings argue against clonal variation or GS1 genotype as the primary explanation for the GS-knockout phenotype.

### Growth characteristics and antibody production of GS-KO cell lines

To assess the recombinant-protein production capacity of the exon-specific PTC-edited lines, we performed fed-batch cultivation for production of an anti-PD-1 monoclonal antibody. Before the production run, growth (viable cell density and viability) was profiled under varying glutamine regimes (Fig. S5). Consistent with earlier characterization, the E6 line sustained proliferation across all glutamine concentrations, consistent with its detectable residual GS protein (Fig. S5e, m). Most other GS-edited lines shared a common growth pattern in 6 mM glutamine over the 9-day culture: rapid proliferation to a peak density, followed by a gradual decline to a lower steady state, with viability beginning to decrease around day 5 (Fig. S5). The E7 line displayed a distinct, manufacturing-relevant phenotype characterized by slower growth kinetics and a lower peak VCD (Fig. S5f, n), but superior viability retention, remaining above 90% throughout cultivation in 6 mM glutamine (Fig. S5n). This robust viability was consistent with enhanced tolerance to culture-associated stress, distinguishing E7 from the other GS-edited derivatives.

To evaluate production potential, we performed antibody production using a standardized workflow (Fig. 4a). Following electroporation with a single vector encoding both the antibody heavy/light chain cassette and the GS selection marker, stable pools were cultured in a 16-day fed-batch process. Consistent with untransfected parental phenotypes (Fig. S5), the ZN pool proliferated rapidly and peaked at approximately 4.8 × 10^7^ cells/mL on day 9 and subsequently declined (Fig. 4b), whereas EK1 and E7 pools grew more slowly, with the E7 pool reaching a final cell density of 1.2 × 10^7^ cells/mL (Fig. 4b). All three pools maintained high viability (86% − 93%) throughout cultivation (Fig. 4c).

Metabolic profiling revealed stable nutrient consumption and waste accumulation across all lines: glucose remained within 1.06–5.02 g/L (Fig. 4d) and lactate remained at basal levels (0.00–1.09 g/L) in all groups (Fig. 4e). Thus, all engineered lines, including the fully GS-KO E7 variant, exhibited balanced metabolic profiles and high viability under standard fed-batch conditions, confirming their suitability for recombinant-protein manufacturing.

Production performance differed markedly among the lines (Fig. 4f). The E7 line achieved the highest antibody titer and specific productivity (Qp). In the absence of MSX, the E7 pool yielded a final titer of 0.74 g/L at a Qp of 3.7 pg/cell/day, representing more than a three-fold increase in Qp relative to the commercial ZN control (0.39 g/L; Qp = 1.0 pg/cell/day) (Fig. 4f). The mechanism underlying this productivity advantage remains to be determined. Productivity was further enhanced by MSX selection: with 5 µM MSX, the E7 titer increased by 38% to 1.02 g/L with a Qp of 4.9 pg/cell/day, 4.45-fold higher than the MSX-treated ZN pool (Fig. 4f), whereas MSX produced no comparable improvement in the EK1 or ZN pools. The absence of a comparable MSX response in EK1 and ZN should not be interpreted as evidence of a productivity ceiling or a non-functional GS marker, but may instead reflect host-dependent differences in selection stringency and stable-pool enrichment dynamics. Collectively, the exon-7 GS-KO (E7) line exhibited the highest production performance among the tested cell lines and retained responsiveness to MSX selection.

### Transcriptomic profiling reveals distinct expression landscapes among engineered CHO cell lines

**Fig. 5:**
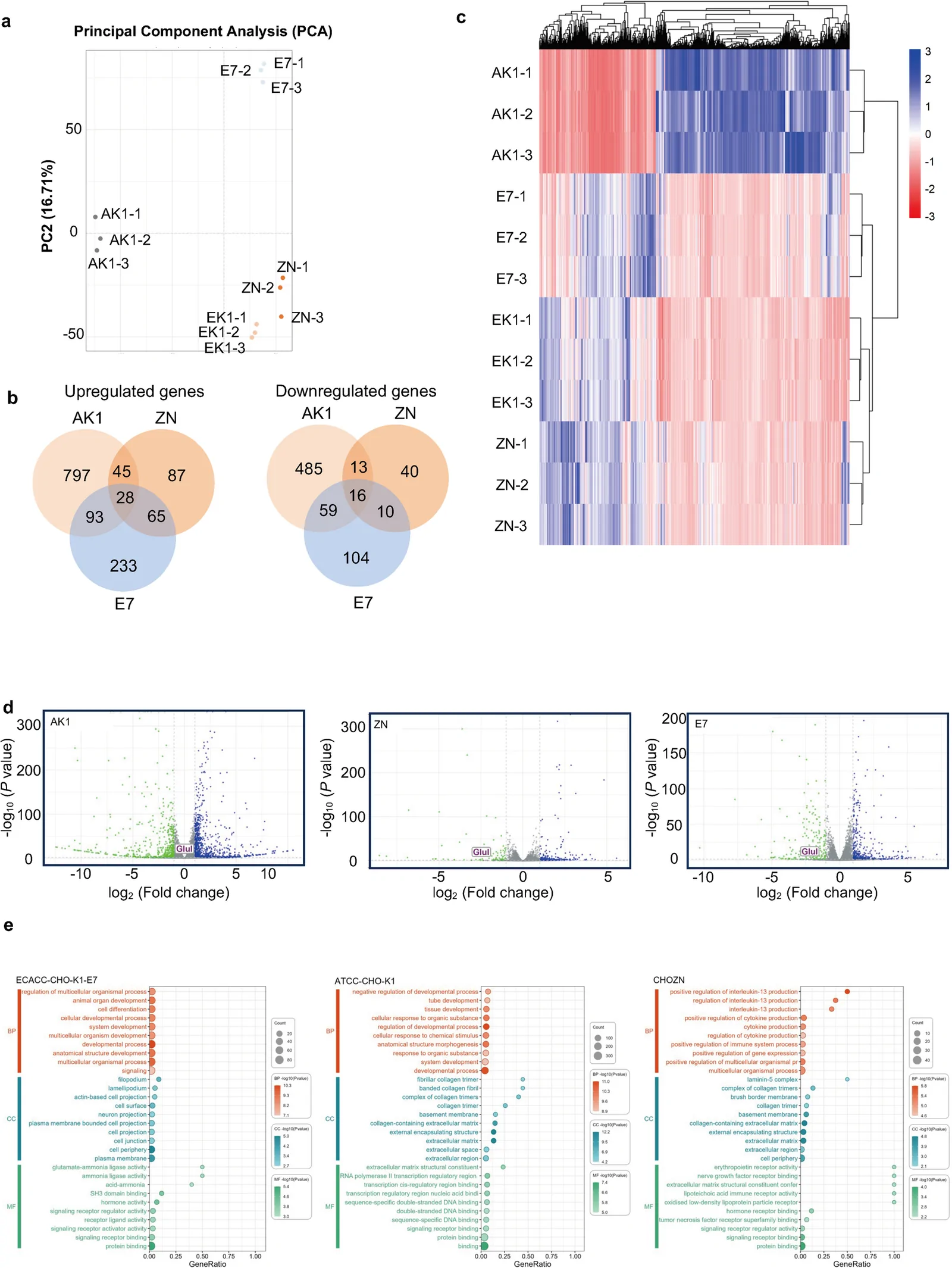
Transcriptomic profiling of GS-knockout CHO cell lines. **a** Principal component analysis of transcriptomes from AK1, ZN, E7, and EK1 cells, with three independent biological replicates per cell line. **b** Venn diagrams showing overlaps among significantly upregulated (left) and downregulated (right) genes in AK1, ZN, and E7 relative to EK1. **c** Hierarchical clustering heatmap of differentially expressed genes (DEGs). Rows represent genes and columns represent biological replicates; scaled expression is shown from relatively low (red) to relatively high (blue). **d** Volcano plots of differential expression in AK1, ZN, and E7 relative to EK1. Significantly upregulated genes are blue, significantly downregulated genes are green, and non-significant genes are gray. DEGs in (b-d) were defined by |log₂ fold change| > 1 and Benjamini-Hochberg-adjusted *P* < 0.05. **e** Gene Ontology enrichment analysis of DEGs in E7, AK1, and ZN relative to EK1. Terms are grouped as biological process (BP), cellular component (CC), and molecular function (MF); bubble size indicates gene count and color indicates enrichment significance

To define the intrinsic transcriptional differences driven by distinct GS-KO patterns, we performed RNA-seq on non-producing cell lines (no exogenous antibody expression), leveraging well-annotated CHO genomic datasets (Kretzmer et al. 2022; Lewis et al. 2013; Schmidt et al. 2024; Xu et al. 2011). Principal-component analysis (PCA) revealed clear transcriptional segregation among the four lines (AK1, ZN, EK1 and E7), with tight intragroup clustering indicating high reproducibility and distinct per-line signatures (Fig. 5a). Differential‑expression analysis using a false discovery rate (FDR) threshold of < 0.05 revealed the most extensive remodeling in AK1 (1,536 differentially expressed genes [DEGs]; 963 up‑regulated, 573 down‑regulated). ZN remained closest to EK1 (304 DEGs; 225 up, 79 down), and E7 was intermediate (608 DEGs; 419 up, 189 down) (Fig. 5b, d). Hierarchical clustering confirmed this gradient: AK1 was transcriptionally most distant from EK1, whereas ZN and E7 shared over 92% of co‑regulated signatures with EK1 (Fig. 5c).

Functional‑enrichment analysis of E7 DEGs showed significant enrichment in metabolic pathways; in particular, genes mediating glutamate-ammonia ligase activity (GO:0004356) were markedly down‑regulated (*P* < 0.001), reflecting the expected metabolic rewiring upon complete GS ablation. Consistently, down‑regulated E7 genes were enriched in nitrogen‑assimilation-related GO terms, including glutamate-ammonia, ammonia, and acid-ammonia ligase activities (Fig. 5e), a molecular hallmark of GS deficiency that disrupts the intrinsic nitrogen‑recycling system of CHO cells. This pattern was not universal: neither ZN nor AK1 showed significant suppression of these glutamine‑synthesis pathways, consistent with their higher residual *GS5* transcript levels, whereas near‑complete *GS5* ablation in E7 produced the most pronounced suppression. Because profiling used non‑producing parental cells, these signatures reflect intrinsic metabolic consequences of distinct GS‑editing patterns rather than productivity‑related differences. Collectively, exon‑specific PTC insertion induces graded transcriptional remodeling, with E7 showing the most pronounced suppression of nitrogen metabolism among the engineered lines.

### Off-target assessment of the base-edited E7 cell line

To evaluate the specificity of the CBE, we first performed targeted sequencing of sgRNA‑dependent candidate off‑target loci in E7. Targeted sequencing revealed that among 13 computationally predicted off-target sites, only the *GS1* locus showed complete C‑to‑T conversion; this locus is annotated as a noncoding pseudogene in the current CHO genome database (Fig. S7a). Transcriptome‑wide variant analysis across AK1, ZN, and E7 revealed no significant increase in exonic, intronic, or UTR SNPs or indels relative to EK1 (Benjamini-Hochberg‑adjusted *P* > 0.05; Fig. S7b, c), indicating a negligible mutational burden. PCR‑based detection further confirmed complete elimination of residual base‑editor cassettes in all clonal lines (E2-E7; Fig. S7d). Together, these analyses support a low detectable off-target burden within the scope of the assays performed; the minimal off‑target activity and thorough vector clearance support the reliability and biosafety of these lines as chassis for recombinant‑protein manufacturing.

## Discussion

In this study, we systematically introduced PTCs into each of the six coding exons of the Chinese hamster *Glul* gene and demonstrated that knockout outcomes depend strongly on PTC position. The resulting cell lines differed substantially in residual *Glul* transcript abundance, detectable GS protein, glutamine dependence, and recombinant antibody production. In particular, E4 and E7 showed near-complete depletion of GS transcripts through an UPF1-dependent mechanism, whereas E6 retained detectable GS protein and proliferated in glutamine-free medium. Among the edited lines, E7 combined complete functional GS deficiency with sustained viability and the highest antibody titer and cell-specific productivity. These findings indicate that GS knockout cannot be regarded as a uniform binary state and that the position of the introduced PTC should be considered when designing GS-deficient CHO production hosts.

The differential transcript abundance observed among the exon-specific mutants was partly attributable to NMD. UPF1 depletion markedly restored GS transcripts in E4 and E7, whereas substantially weaker or absent responses were observed in the other edited and control cell lines. This result provides functional evidence that the exceptionally low abundance of the Q135X- and R286X-containing transcripts is largely mediated by UPF1-dependent NMD. However, NMD susceptibility alone did not fully predict the functional outcome. Several edited lines, particularly E2 and E3, retained appreciable GS transcripts but showed no detectable GS protein, whereas E6 retained both residual transcripts and GS protein. Thus, residual PTC-containing transcripts should not be interpreted as direct evidence of residual protein expression or GS activity. The distinct positions of Q135X and R286X within the annotated GS domain structure may influence transcript processing, translation, or the properties of the resulting decay intermediates (Fig. S6a). Recent evidence has shown that cytoplasmic degradation products of translation-defective transcripts can participate in transcriptional adaptation through RNA‑binding proteins (El-Brolosy et al. 2026). Nevertheless, the present data do not establish whether exon-specific GS decay intermediates exert regulatory effects in cis or trans, and this possibility will require direct characterization of the relevant RNA species.

Although both E4 and E7 exhibited profound GS transcript depletion and undetectable GS protein, E7 showed substantially better production performance, indicating that the superior phenotype of E7 cannot be explained solely by the extent of GS knockout. Targeted RT-qPCR revealed that E4 and E7 occupied distinct metabolic and stress-response states (Fig. S6b-f). Compared with E4, E7 showed lower expression of multiple genes involved in glutamine transport and nitrogen metabolism, including *Gls*, *Glud1*, *Got1*, *Got2*, *Slc1a5*, and *Slc38a2*, together with reduced expression of the proliferation-associated genes *Myc* and *Mki67*. Basal ER-stress/UPR markers, including *Hspa5* and *Xbp1s*, were also lower in E7, whereas the canonical amino-acid stress-response genes *Atf4*, *Asns*, and *Sesn2* were not significantly induced relative to E4. Consistent with these targeted measurements, transcriptomic analysis showed pronounced remodeling of nitrogen-metabolism pathways in E7, including reduced enrichment of downregulated genes in glutamate-ammonia ligase-related functions (Fig. 5e). The coordinated reduction in nitrogen metabolism, proliferation, and basal ER-stress markers is consistent with the slower-growth, high-viability phenotype of E7 and may favor the allocation of cellular resources toward recombinant protein production. However, these associations do not demonstrate that the expression changes were directly caused by the R286X position or that they were responsible for the increased productivity. Direct perturbation of the affected pathways will be needed to establish causality.

The response of E7 to MSX selection provides an additional potential explanation for its production advantage. Because the antibody heavy chain, light chain, and GS selectable marker were encoded on the same vector, MSX selection may enrich cells with stronger expression of the linked antibody cassette. The near-complete endogenous GS deficiency of E7 may impose more stringent selection, thereby facilitating enrichment of high-producing subpopulations. In contrast, EK1 and ZN did not show a comparable improvement during the 16-day stable-pool selection period, possibly because of host-dependent differences in endogenous GS activity, selection stringency, or pool composition. The slower growth but higher Qp of E7 is also compatible with a growth-productivity trade-off, in which reduced proliferative demand allows greater allocation of biosynthetic capacity to antibody production (Quek et al. 2022). These explanations remain hypotheses because transgene copy number, integration pattern, clonal composition, and cellular resource allocation were not directly examined.

The potential contribution of the homologous *GS1* locus was also considered. The E7-targeting sgRNA showed complete sequence identity to the corresponding *GS1* site, resulting in different *GS1* editing states among independently derived E7 clones (Fig. S4a). Nevertheless, GS protein remained undetectable in E7 clones with homozygously edited, heterozygously edited, or unedited *GS1* loci (Fig. S4e). In addition, *GS1* transcript abundance remained comparable to that in EK1 in most edited lines, indicating an absence of substantial compensatory *GS1* upregulation following *GS5* disruption (Fig. S4b). These results, together with the current annotation of *GS1* as a noncoding pseudogene, suggest that *GS1* editing is unlikely to be the principal determinant of the E7 phenotype. However, because the biological functions of pseudogene-derived transcripts are not always fully understood, a subtle contribution of *GS1* cannot be completely excluded.

Cytosine base editing also offers methodological advantages for generating defined GS-knockout hosts. Conventional ZFN, TALEN, and CRISPR/Cas9 strategies rely on double-strand breaks and error-prone repair, producing heterogeneous mixtures of frameshift and in-frame alleles. In contrast, CBE enables direct conversion of selected codons into PTCs without double-strand cleavage, thereby generating more predictable genotypes and facilitating exon-resolved comparison. Avoiding double-strand breaks may also reduce the risk of large deletions, chromosomal rearrangements, and DNA-damage responses associated with nuclease-mediated editing (Fiumara et al. 2024; Hwang et al. 2024; Komor et al. 2016). In E7, targeted sequencing identified editing only at the homologous GS1 locus among the 13 predicted off-target sites examined (Fig. S7a). RNA-seq-based variant analysis showed no significant increase in the overall burden of expressed SNPs or indels relative to the parental control (Fig. S7b, c), and neither the editor nor sgRNA plasmid sequence was detected in the established monoclonal lines (Fig. S7d). These results support the genetic integrity of the edited lines, although whole-genome sequencing would be required to exclude rare variants or alterations in unexpressed genomic regions.

Several limitations should be acknowledged. Most exon-specific phenotypic comparisons were performed using representative monoclonal lines, although additional E2 and E7 clones were evaluated by sequencing and Western blotting. Moreover, several growth and fed-batch experiments were conducted as single standardized runs on an industrial CDMO platform rather than as independent biological replicates. The production results should therefore be validated through independent fed-batch experiments and clone-level production studies. The mechanisms responsible for the divergence between E4 and E7 also remain unresolved, and the current RT-qPCR and transcriptomic data establish associations rather than direct causal relationships. Finally, targeted off-target sequencing and RNA-seq-based variant analysis cannot replace comprehensive whole-genome assessment.

In conclusion, our findings identify PTC position as an important determinant of transcript fate, protein loss, and functional knockout outcome in CHO cells. Exon 7-targeted introduction of R286X generated an E7 host with complete GS deficiency, sustained viability, and high recombinant antibody productivity. More broadly, exon-resolved PTC engineering provides a rational strategy for generating predictable knockout phenotypes and may facilitate the development of improved metabolic selection systems for mammalian cell manufacturing (Raab et al. 2024).

## Supplementary Information

Below is the link to the electronic supplementary material.

Supplementary Material 1

Supplementary Material 2

Supplementary Material 3

Supplementary Material 4

Supplementary Material 5

Supplementary Material 6

Supplementary Material 7

Supplementary Material 8

Supplementary Material 9

Supplementary Material 10

## Data Availability

The raw RNA-seq data generated in this study have been submitted to the NCBI Sequence Read Archive (SRA) under BioProject accession number PRJNA1516528.

## Declarations

### Human and animal participants

This study does not involve human subjects, human data, or animal experiments.
