# Supplementary material for: Base editing-mediated exon-specific knockout of glutamine synthetase generates a high-productivity CHO cell line for antibody manufacturing
Source: Bioresour Bioprocess. 2026 Oct 23;13(1):156. doi: 10.1186/s40643-026-01150-0 (PMC13694664; doi:10.1186/s40643-026-01150-0)

**Supplementary Figure 1d**

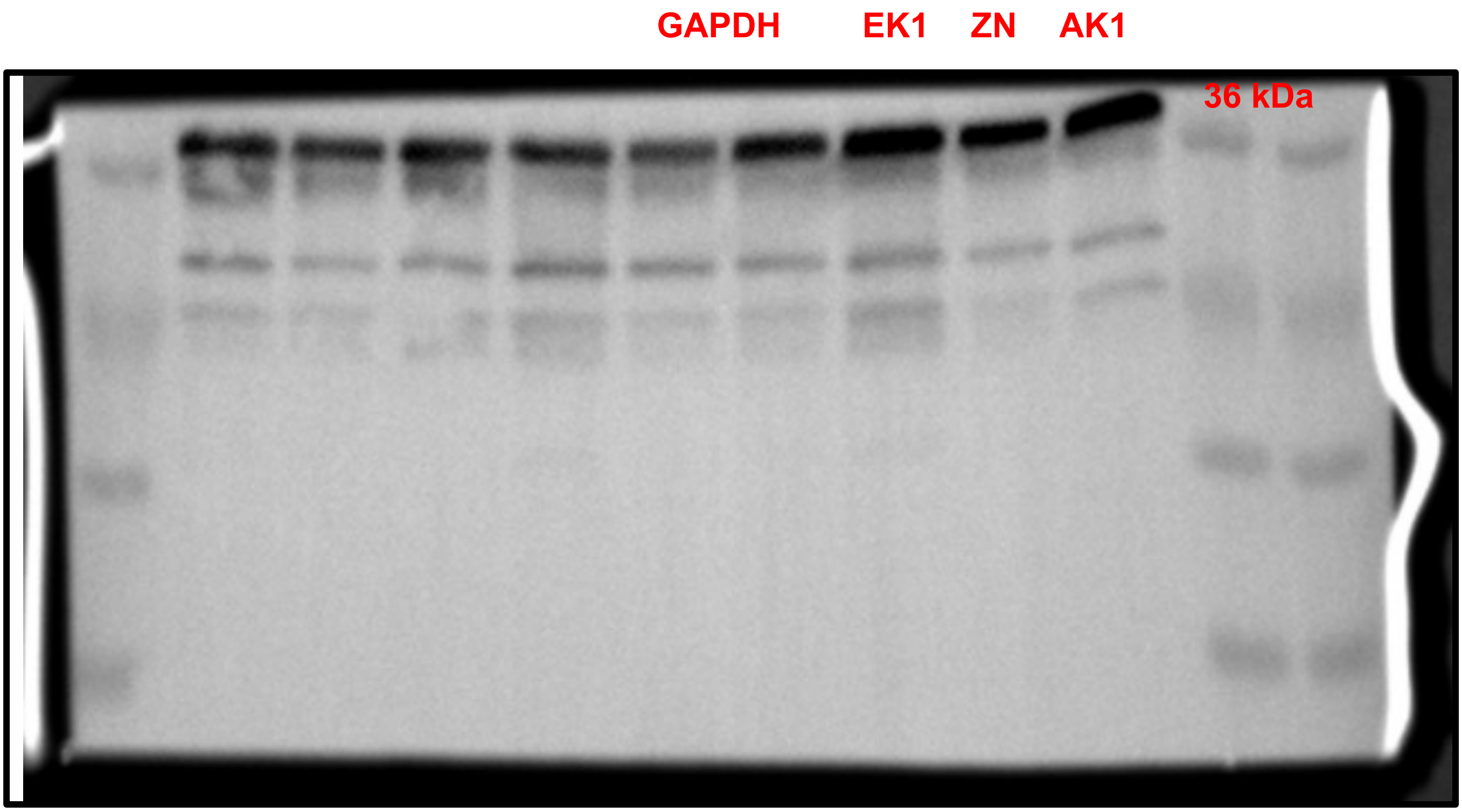


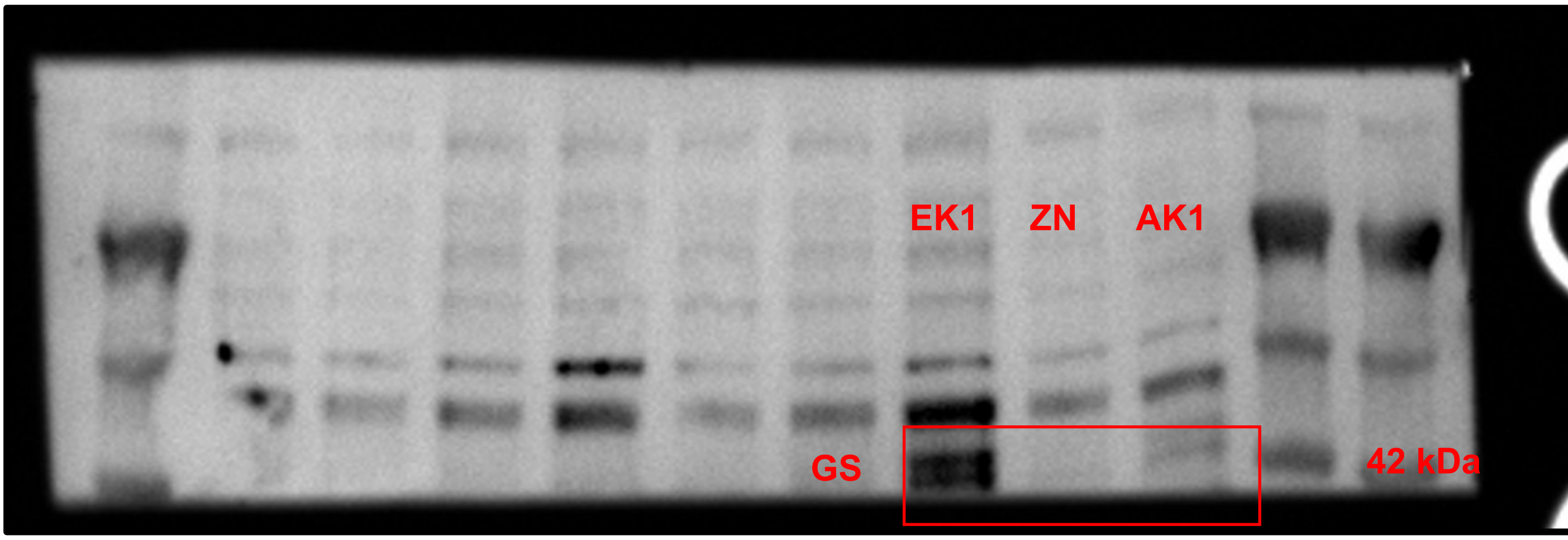

Figure 2e

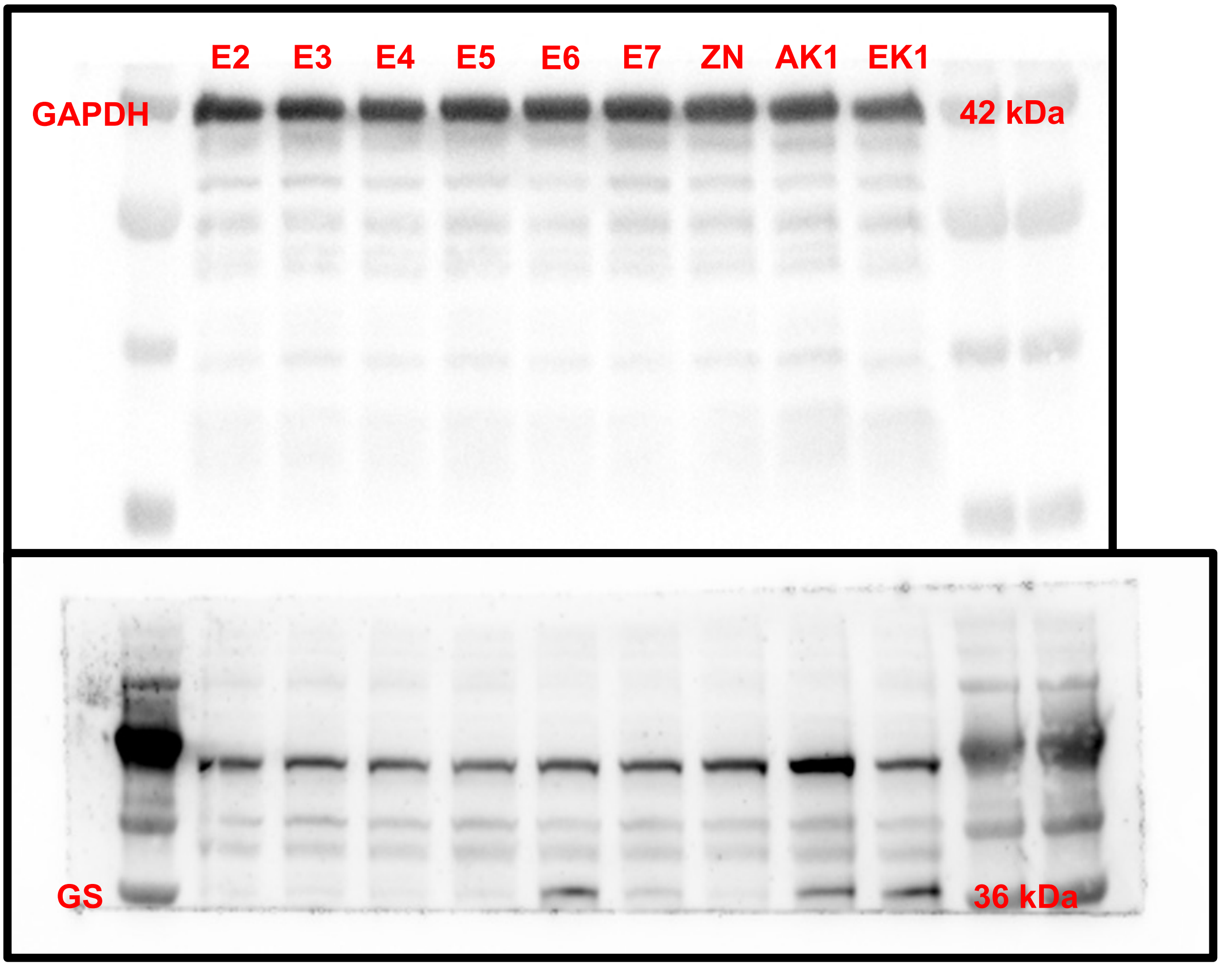

## Supplementary Figure 2d

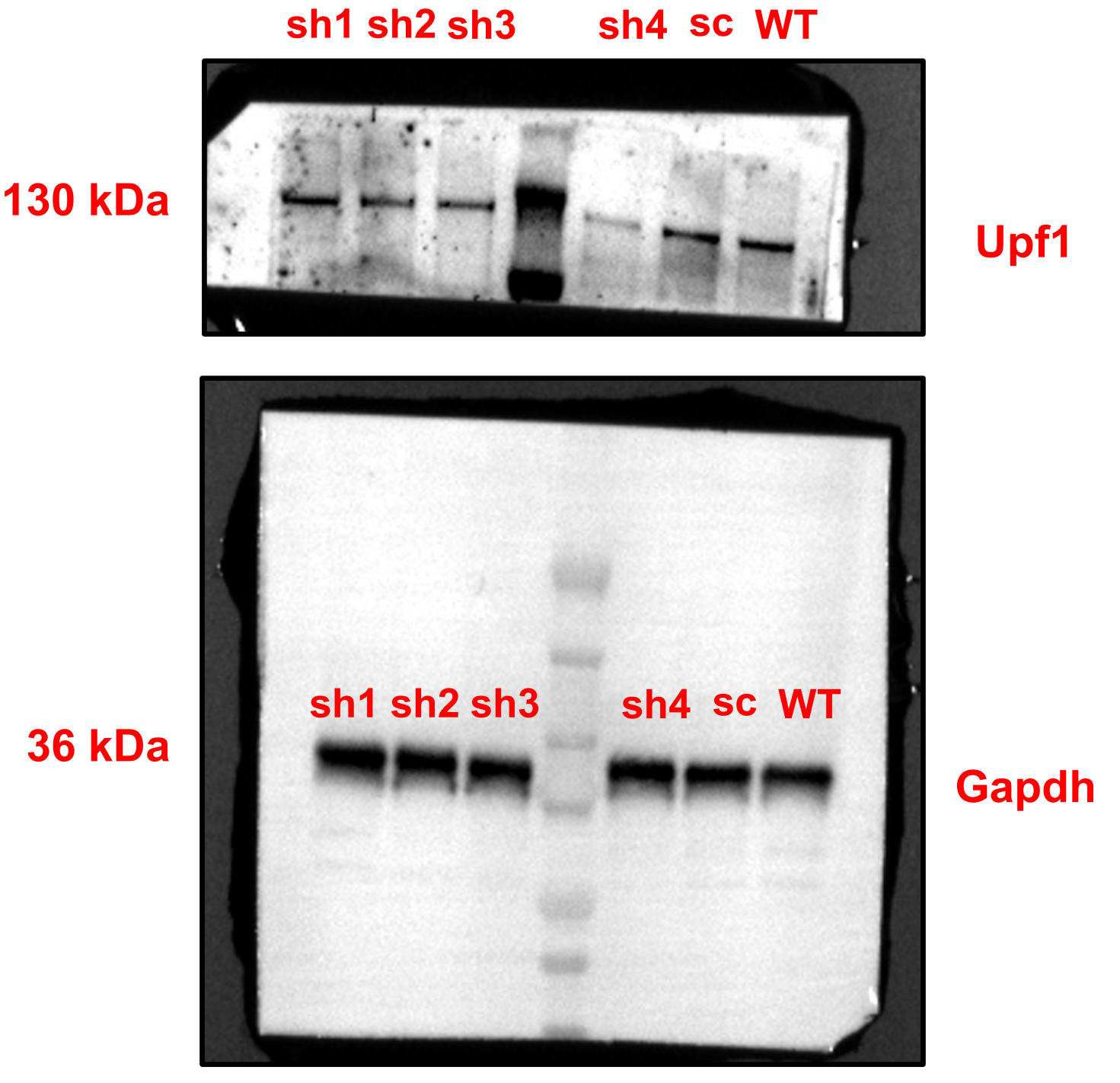

Supplementary Figure 4e

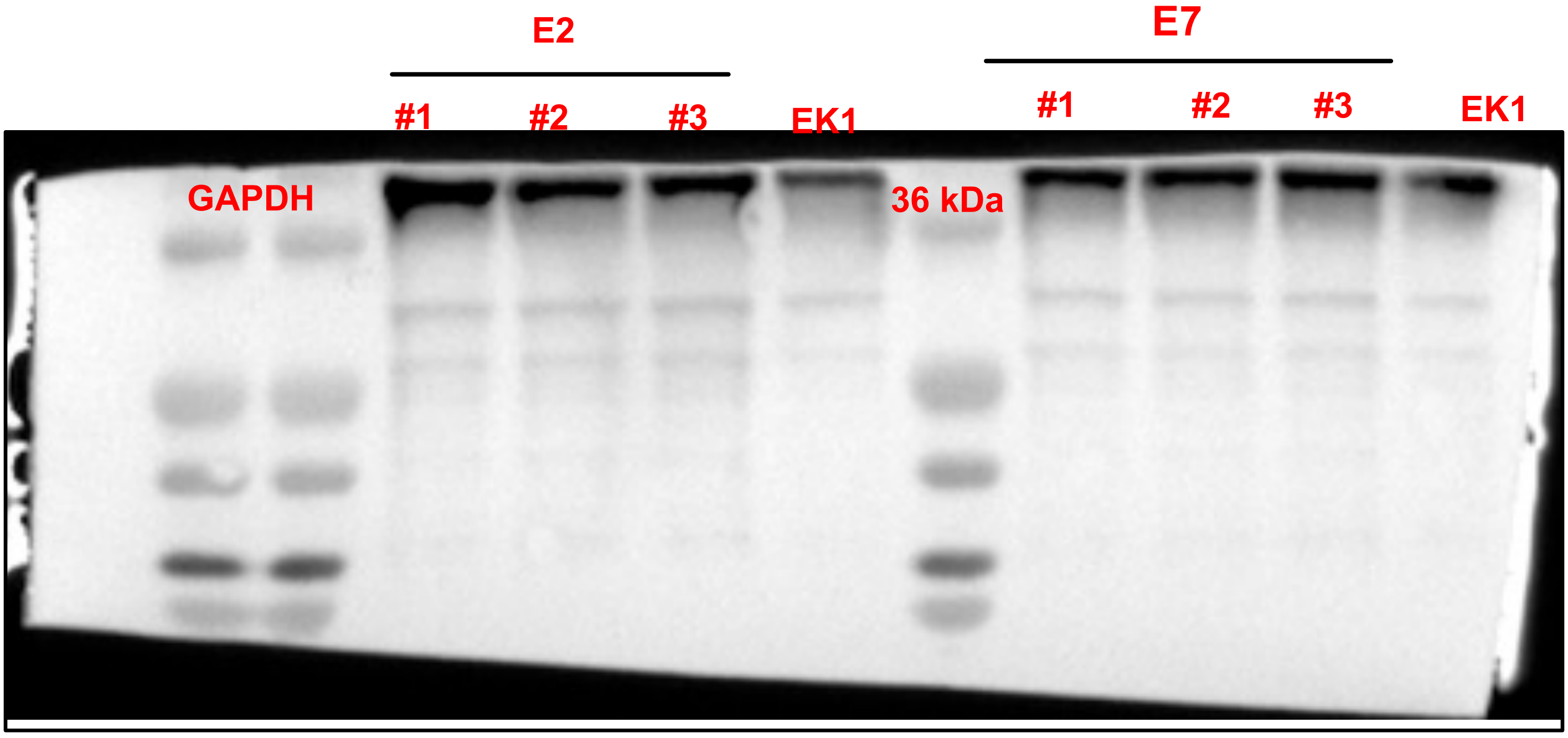


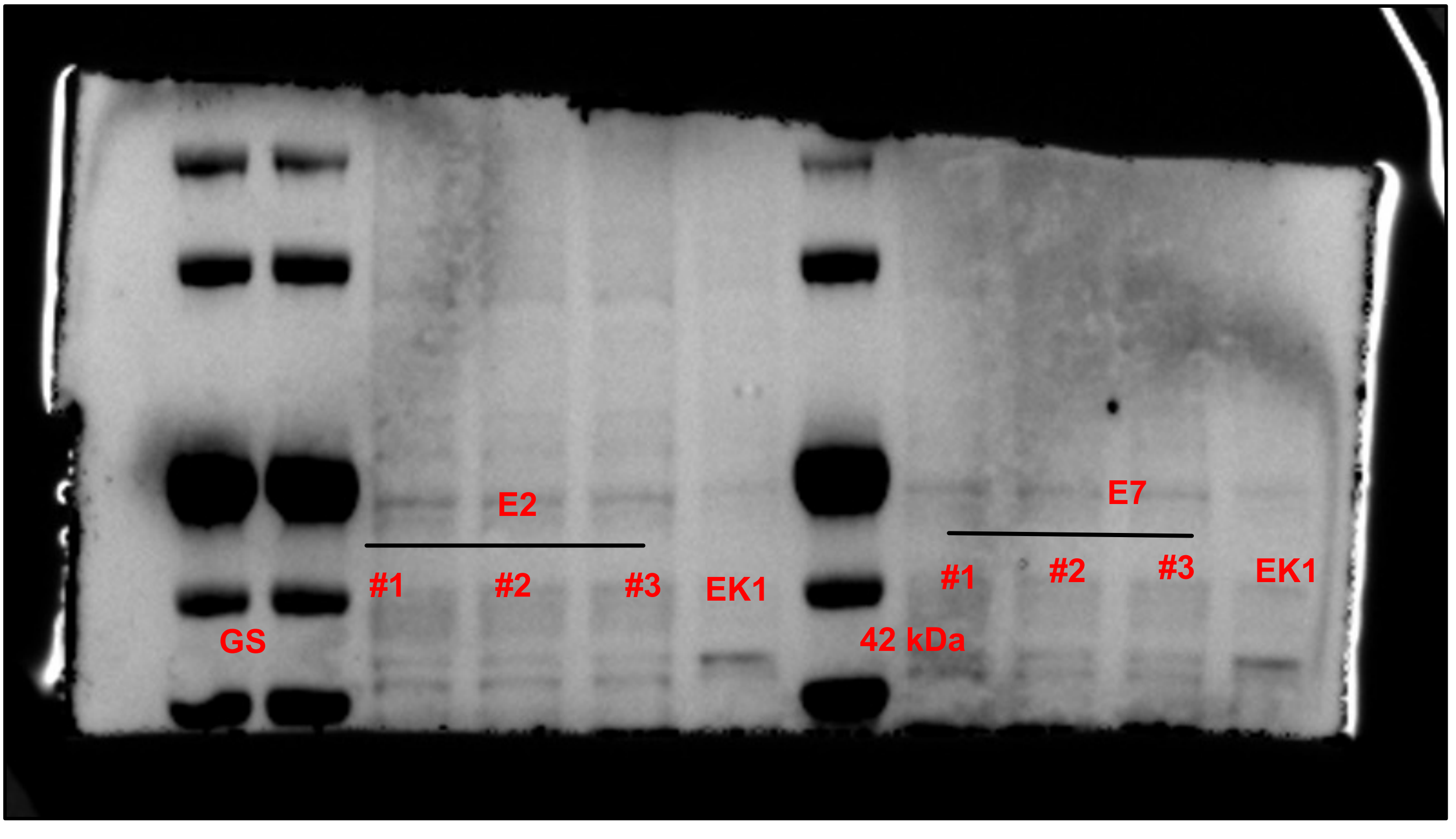

**Supplementary Figure 6d**

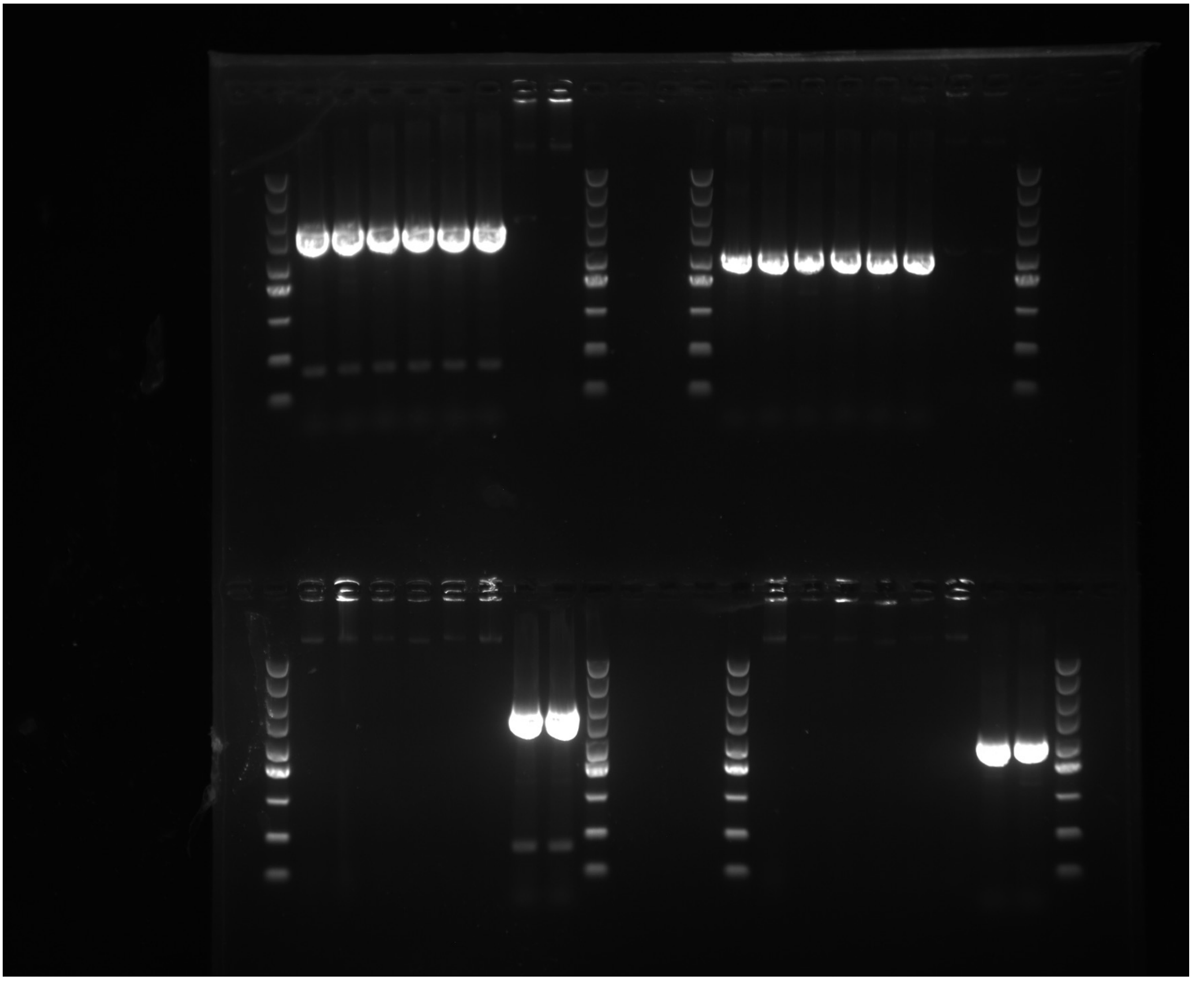

Supplement: Supplementary file 1 [file 40643_2026_1150_MOESM9_ESM.pdf]
