# Supplementary material for: Base editing-mediated exon-specific knockout of glutamine synthetase generates a high-productivity CHO cell line for antibody manufacturing
Source: Bioresour Bioprocess. 2026 Oct 23;13(1):156. doi: 10.1186/s40643-026-01150-0 (PMC13694664; doi:10.1186/s40643-026-01150-0)
Supplement: Supplementary file 2 [file 40643_2026_1150_MOESM10_ESM.docx]

Table S1

sgRNA sequence

| sgRNA | Sequence |
| --- | --- |
| BE-sgRNA-Exon 2 up | ACCGAGTCCAAGCCATGTATATCT |
| BE-sgRNA-Exon 2 down | AAACAGATATACATGGCTTGGACT |
| BE-sgRNA-Exon 3 up | ACCGAATTCCACTCAGGTAACTCT |
| BE-sgRNA-Exon 3 down | AAACAGAGTTACCTGAGTGGAATT |
| BE-sgRNA-Exon 4 up | ACCGGGAACAGGAGTATACTCTGA |
| BE-sgRNA-Exon 4 down | AAACTCAGAGTATACTCCTGTTCC |
| BE-sgRNA-Exon 5 up | ACCGCCTGCCCAGGTAAATGGCAC |
| BE-sgRNA-Exon 5 down | AAACGTGCCATTTACCTGGGCAGG |
| BE-sgRNA-Exon 6 up | ACCGTTCCAAATAGGACCCTGTGA |
| BE-sgRNA-Exon 6 down | AAACTCACAGGGTCCTATTTGGAA |
| BE-sgRNA-Exon 7 up | ACCGATTCGAGCCTACGATCCCAA |
| BE-sgRNA-Exon 7 down | AAACTTGGGATCGTAGGCTCGAAT |
| Cas9-sgRNA-1 up | ACCGTTCTAGTGGGAATTCCAAAT |
| Cas9-sgRNA-1 down | AAACATTTGGAATTCCCACTAGAA |
| Cas9-sgRNA-2 up | ACCGTTCCAAATAGGACCCTGTGA |
| Cas9-sgRNA-2 down | AAACTCACAGGGTCCTATTTGGAA |
| Cas9-sgRNA-3 up | ACCGACCCTGTGAAGGAATCCGCA |
| Cas9-sgRNA-3 down | AAACTGCGGATTCCTTCACAGGGT |
| Cas9-sgRNA-4 up | ACCGTCCGCATGGGAGATCATCTC |
| Cas9-sgRNA-4 down | AAACGAGATGATCTCCCATGCGGA |
| Cas9-sgRNA-5 up | ACCGCATCGAGTATGTGAAGACTT |
| Cas9-sgRNA-5 down | AAACAAGTCTTCACATACTCGATG |
| Cas9-sgRNA-6 up | ACCGTTTGACCCCAAGCCCATTCC |
| Cas9-sgRNA-6 down | AAACGGAATGGGCTTGGGGTCAAA |
| Cas9-sgRNA-7 up | ACCGCCAAGCCCATTCCTGGGAAC |
| Cas9-sgRNA-7 down | AAACGTTCCCAGGAATGGGCTTGG |
| Cas9-sgRNA-8 up | ACCGCCCATTCCTGGGAACTGGAA |
| Cas9-sgRNA-8 down | AAACTTCCAGTTCCCAGGAATGGG |
| Cas9-sgRNA-9 up | ACCGCCTGGGAACTGGAATGGTGC |
| Cas9-sgRNA-9 down | AAACGCACCATTCCAGTTCCCAGG |
| Cas9-sgRNA-10 up | ACCGCCATACCAACTTTAGCACCA |
| Cas9-sgRNA-10 down | AAACTGGTGCTAAAGTTGGTATGG |
|  |  |
|  |  |

Genotyping PCR primer

| Target region | Forward |
| --- | --- |
| *GS*-Exon 2 F | CCAACCTACAAGGGATGATC |
| *GS*-Exon 2 R | TCTATGCCTCCAGACTACTC |
| *GS*-Exon 3 F | TCCCAGGAACTGAAGTTAAC |
| *GS*-Exon 3 R | GTTTTTCAGCAGGCAAGTTC |
| *GS*-Exon 4 F | TTTGCTCTCACTCCATTCCC |
| *GS*-Exon 4 R | TCTCATCACCATCCTGTCAC |
| *GS*-Exon 5 F | GCAAGTCTTAGGACCTTGTC |
| *GS*-Exon 5 R | GGGTCCTATTTGGAATTCCC |
| *GS*-Exon 6 F | CAGGAACAAATGCTGAGGTC |
| *GS*-Exon 6 R | TAACACAAAGCCCTTCCACC |
| *GS*-Exon 7 F | TCTTACTGATGGGAAGAGGC |
| *GS*-Exon 7 R | CTCATTGAGAAGGCATGTGC |
| GS-ZFN F | GCAAGTCTTAGGACCTTGTC |
| GS-ZFN R | GGGTCCTATTTGGAATTCCC |
| GS-TALEN F | GCAAGTCTTAGGACCTTGTC |
| GS-TALEN R | GGGTCCTATTTGGAATTCCC |
| CBE4max-SpRY F | TGAAGTGTTCTTTGACCCCC |
| CBE4max-SpRY R | TGATCTCGGTGTTCACTCTC |
| sgRNA-BSD F | TGAAAAAGTGGCACCGAGTC |
| sgRNA-BSD R | TGAAGAAGTCGTGCTGCTTC |

RT-qPCR primer

| Primer | Sequence |
| --- | --- |
| GS qPCR Exon 2 F | ATGGCCACCTCAGCAAGTTC |
| GS qPCR Exon 2 R | CGCAGTCCTTCTCCAGTACC |
| GS qPCR Exon 4 F | TTAAGGCACTCGTGTAAACGG |
| GS qPCR Exon 4 R | GAAAGCCATTGGAAGGCCAAC |
| GS qPCR Exon 7 F | CGGCCAGGAGAAGAAAGGTT |
| GS qPCR Exon 7 R | ATTGAGAAGGCATGTGCGGA |
| *Gapdh* qPCR F | GAAAGCTGTGGCGTGATGG |
| *Gapdh* qPCR R | ACACGTTGGGGGTAGGAACA |
| GS1 qPCR F | GCACATCAAGGAGGCCATT |
| GS1 qPCR R | CTGCGATCGGCGACGCCAGCT |
| *Upf1* qPCR F | CAGACTTAACTCAGCGGCCA |
| *Upf1* qPCR R | TCAATGCACTCTGCATCCTATCA |
| *Atf4* qPCR F | GGCGTATTAGAGGCAGCAGT |
| *Atf4* qPCR R | GGATTCCGTGAAAAACGCCA |
| *Ddit3* qPCR F | TTCGTTATCCCCGGCTCTCC |
| *Ddit3* qPCR R | AGTTGGACTGGGTTCTGCTTT |
| *Ppp1r15a* qPCR F | TTTACGGGCCAGAAAGGTGC |
| *Ppp1r15a* qPCR R | TATCTCGTGCAAGCTGCTCC |
| *Gls* qPCR F | GATTTCTCAGGGCAGTTTGC |
| *Gls* qPCR R | TAACACTGTTGCCCATCTTG |
| *Glud1* qPCR F | CCAGGGTTTGGTGATAAGAC |
| *Glud1* qPCR R | CAGATTCACCAACACCAACAC |
| *Got1* qPCR F | GTCTATGTGTCGTCGCCAAC |
| *Got1* qPCR R | TCTTCTCTGCGTCCCAGTAC |
| *Got2* qPCR F | TCGAGATGACAACGGGAAGC |
| *Got2* qPCR R | CTTGCAGAATTCCGCCAGC |
| *Slc1a5* qPCR F | TCCGCCAGCTGTTCATCAGA |
| *Slc1a5* qPCR R | GAATCGATAGGGGTTTTGGC |
| *Slc38a2* qPCR F | GCTGTGTTGATGGCTGTCAC |
| *Slc38a2* qPCR R | TGACGCCACCAACTGAACTC |
| *Asns* qPCR F | TGGCATTTGGGCCCTCTTTG |
| *Asns* qPCR R | CAAAGCAGCAGTTGGTATATCC |
| *Sesn2* qPCR F | AGTGTTCTTACCTGGTGGGC |
| *Sesn2* qPCR R | TCTCACTGAGTTTGCGAAGC |
| *Myc* qPCR F | GATCAGCTGGAGATGGTGAC |
| *Myc* qPCR R | AGCCGCTCCACATACAATCC |
| *Ccnd1* qPCR F | AGAAGTGCGAAGAGGAGGTC |
| *Ccnd1* qPCR R | GTCTCCTTCATCTTGGAGGC |
| *Mki67* qPCR F | GAAGATCTTCAGTGCATGAC |
| *Mki67* qPCR R | AGTGGACTCTCCTAGGTTTC |
| *Bcl2* qPCR F | CCACTAAGGCCCATAGTCGC |
| *Bcl2* qPCR R | CTGGACATCTCCGCGAAGTC |
| *Bax* qPCR F | GGGGATACACCTGAGCTGAC |
| *Bax* qPCR R | CTGCAGCTCCATGTTGCTGTC |
| *Mcl1* qPCR F | ATGGCATAACAAACTGGGGC |
| *Mcl1* qPCR R | TTGTTTGACAAGCCAGTCCC |
| *Casp3* qPCR F | GAACAGATGGGCCTGTTGAC |
| *Casp3* qPCR R | CAATGCCACAGTCCAGTTCC |
| *Hspa5* qPCR F | GTGCAGAAACTTCGTCGTGA |
| *Hspa5* qPCR R | TTCTGGACTGGCTTCATGGT |
| *Xbp1s* qPCR F | CTCCAGAGACGGAGTCCAAG |
| *Xbp1s* qPCR R | CTGCACCTGCTGCGGACTC |
|  |  |
|  |  |

shRNA sequence

|  | Sequence |
| --- | --- |
| Upf1 shRNA 1 | GGAGTCACAGACTCAAGATAACTCGAGTTATCTTGAGTC  TGTGACTCC |
| Upf1 shRNA 2 | GCTCCTACCTGGTGCAATATACTCGAGTATATTGCACCA  GGTAGGAGC |
| Upf1 shRNA 3 | GCCGGCCATCAAACATGTACTCTCGAGAGTACATGTTTG  ATGGCCGGC |
| Upf1 shRNA 4 | GGAAGTACTTTGATCAGTTTGCTCGAGCAAACTGATCAA  AGTACTTCC |
| Scramble shRNA | CCTAAGGTTAAGTCGCCCTCGCTCGAGCGAGGGCGACTT  AACCTTAGG |

Off-target site PCR primer

|  | Sequence |
| --- | --- |
| on target F | TCGTCGGCAGCGTCCTGCTTGTATGCTGGGGTCA |
| on target R | GTCTCGTGGGCTCGGATTGAGAAGGCATGTGCGGA |
| off target site1 F | TCGTCGGCAGCGTCGCTTGTTTGCTGGGGTCAAG |
| off target site1 R | GTCTCGTGGGCTCGGAGGGCTTGTTGTCAGTCTCG |
| off target site2 F | TCGTCGGCAGCGTCCAGTGAGGACCAGGAGCAAG |
| off target site2 R | GTCTCGTGGGCTCGGTGTGGATGGGTCAGGAGACT |
| off target site3 F | TCGTCGGCAGCGTCTGATTCTGCAGGGCCATCTG |
| off target site3 R | GTCTCGTGGGCTCGGTCCTCTCGAGACTTCTGCTCA |
| off target site4 F | TCGTCGGCAGCGTCAGGGATTATGCCAGGCCCTA |
| off target site4 R | GTCTCGTGGGCTCGGACTGGCTCCTCATGTCTTGC |
| off target site5 F | ACAACACTGGGAAGGAAAACA |
| off target site5 R | TTTTCTCCCACTGAAGCAGCC |
| off target site6 F | TCGTCGGCAGCGTCGCTGAGCTCTGTACATGGGG |
| off target site6 R | GTCTCGTGGGCTCGGACGGGTGCCAGGTAGATAGT |
| off target site7 F | GACTGATGCTCACTCGACTCT |
| off target site7 R | GGTTAGTGATGCACAGGT |
| off target site8 F | TCGTCGGCAGCGTCACAACACTGGGAAGGAAAACA |
| off target site8 R | GTCTCGTGGGCTCGGGGCTGCTTCAGTGGGAGAAA |
| off target site9 F | TCGTCGGCAGCGTCTTCATCTGAGGATGGCGGTG |
| off target site9 R | GTCTCGTGGGCTCGGTGTAACAGCTCGGCAGTTGT |
| off target site10 F | TCGTCGGCAGCGTCGACTGATGCTCACTCGACTCT |
| off target site10 R | GTCTCGTGGGCTCGGACCTGTGCATCACTAACCAT  CA |
| off target site11 F | TCGTCGGCAGCGTCCTGTGTCACTGTATGCCGGA |
| off target site11 R | GTCTCGTGGGCTCGGTTGGCTAAGCTGTCTGGTCG |
| off target site12 F | TCGTCGGCAGCGTCGGCTTTTGTGGGACTAAATGT  GA |
| off target site12 R | GTCTCGTGGGCTCGGAGTGAAAACATACATCTGCA  CCA |
| off target site13 F | TCGTCGGCAGCGTCGGGGCTCAGAGTTAGTGCTC |
| off target site13 R | GTCTCGTGGGCTCGGGACTTCCAGACTGGTGTGGG |

Sequence of plasmid

ZFNGSB9372-R: GCAAAAACAGGAAGGCAAAATGCCGCAAAAAAGGGAATAAGGGCGACACGGAAATGTTGAATACTCATACTCTTCCTTTTTCAATATTATTGAAGCATTTATCAGGGTTATTGTCTCATGAGCGGATACATATTTGAATGTATTTAGAAAAATAAACAAATAGGGGTTCCGCGCACATTTCCCCGAAAAGTGCCACCTGACGTCGACGGATCGGGAGATCTCCCGATCCCCTATGGTGCACTCTCAGTACAATCTGCTCTGATGCCGCATAGTTAAGCCAGTATCTGCTCCCTGCTTGTGTGTTGGAGGTCGCTGAGTAGTGCGCGAGCAAAATTTAAGCTACAACAAGGCAAGGCTTGACCGACAATTGCATGAAGAATCTGCTTAGGGTTAGGCGTTTTGCGCTGCTTCGCGATGTACGGGCCAGATATACGCGTTGACATTGATTATTGACTAGTTATTAATAGTAATCAATTACGGGGTCATTAGTTCATAGCCCATATATGGAGTTCCGCGTTACATAACTTACGGTAAATGGCCCGCCTGGCTGACCGCCCAACGACCCCCGCCCATTGACGTCAATAATGACGTATGTTCCCATAGTAACGCCAATAGGGACTTTCCATTGACGTCAATGGGTGGAGTATTTACGGTAAACTGCCCACTTGGCAGTACATCAAGTGTATCATATGCCAAGTACGCCCCCTATTGACGTCAATGACGGTAAATGGCCCGCCTGGCATTATGCCCAGTACATGACCTTATGGGACTTTCCTACTTGGCAGTACATCTACGTATTAGTCATCGCTATTACCATGGTGATGCGGTTTTGGCAGTACATCAATGGGCGTGGATAGCGGTTTGACTCACGGGGATTTCCAAGTCTCCACCCCATTGACGTCAATGGGAGTTTGTTTTGGCACCAAAATCAACGGGACTTTCCAAAATGTCGTAACAACTCCGCCCCATTGACGCAAATGGGCGGTAGGCGTGTACGGTGGGAGGTCTATATAAGCAGAGCTCTCTGGCTAACTAGAGAACCCACTGCTTACTGGCTTATCGAAATTAATACGACTCACTATAGGGAGACCCAAGCTGGCTAGCGTTTAAACTTAAGCTTGCCACCATGGCGCCTAAGAAAAAGAGAAAGGTGGGCATCCACGGCGTGCCTGCCGCTATGGCCGAGCGCCCCTTTCAATGTAGAATCTGCATGCGCAATTTCAGCAGAAGCGACAACCTCTCCGAGCACATCAGAACACACACCGGCGAGAAGCCATTTGCCTGCGACATCTGTGGCAGAAAGTTCGCTAAACGGTGCAACCTGAGATGCCACACAAAGATTCACACCCACCCCAGAGCCCCTATCCCCAAGCCTTTTCAGTGCAGAATCTGCATGCGGAATTTCTCTCAGAGCTCTGATCTGAGCAGACATATCCGGACCCACACTGGAGAGAAACCTTTCGCCTGTGATATCTGCGGCAGAAAGTTCGCCCAGTGGAGCACCCGGAAGCGGCACACCAAGATTCACACCGGCGAGAAGCCTTTCCAGTGTAGAATCTGCATGCGGAAATTCGCCCAATCTGGAGATCTGACAAGACACACAAAGATCCACCTGCGGCAGAAGGACGCTGCCAGAGGCAGCGGAATGGACGCCAAGAGCCTGACCGCCTGGTCCATGCTAGAACCAGGTGAAAAGCCATATAAGTGTCCTGAGTGCGGCAAGTCCTTTTCTAGATCTGATCACCTGTCCACCCACCAGAGAACCCATCCTGGCGAGAAGCCTTACAAGTGTCCTGAATGCGGAAAGTCCTTCTCCCAGTCCTCTGACCTGAGAAGACACCAGCGGACCCACCCAGGCGAGAAGCCTTACAAGTGCCCTGAGTGCGGCAAAAGCTTCTCCCGGTCCGACCACCTGTCTCAGCACCAAAGAACACACCCTGGAGAAAAGCCCTACAAGTGCCCCGAGTGTGGCAAGTCTTTCAGCCAGTCTGCCAACCGGACCACCCACCAGCGCACCCATCCTGGCGAGAAACCTTATAAGTGCCCTGAGTGCGGCAAGAGCTTCTCCAGATCCGACAACCTGTCTCAGCATCAGCGGACACACCCCGGCGAAAAACCCTACAAGTGCCCAGAATGTGGCAAGTCCTTCTCTGCTTCCAACGACCGGAAGAAGCACCAGAGAACCCACACCGGCAAGAAGACCCAACTAGTGAAGAGTGAACTAGAAGAAAAGAAGTCCGAGCTGAGACACAAGCTGAAGTACGTGCCTCACGAGTACATCGAGCTGATCGAGATCGCCAGAAACTCCACACAGGACCGGATCCTGGAAATGAAAGTGATGGAATTCTTCATGAAAGTCTATGGCTACAGAGGAAAGCATCTGGGAGGCTCTCGGAAGCCTGATGGCGCCATCTACACCGTGGGCAGCCCCATCGACTACGGCGTGATCGTGGACACCAAGGCCTACTCTGGCGGCTACAACCTGCCTATTGGACAGGCCGACGAGATGCAGAGATACGTCAAAGAGAACCAGACCCGCAACAAGCACATCAATCCTAACGAGTGGTGGAAGGTGTACCCAAGCTCCGTGACCGAGTTCAAGTTTCTGTTCGTGTCCGGCCACTTCAAGGGCAACTACAAGGCTCAACTGACCAGACTGAACAGAAAGACCAACTGCAACGGCGCTGTGCTGTCTGTGGAAGAACTGCTGATCGGCGGCGAGATGATCAAGGCTGGCACCCTGACACTCGAGGAAGTGCGGCGGAAGTTCAACAATGGCGAAATCAACTTTGAACGGGAAATCCACCAGGAGACACATCCTGACAGCGAGACAGCCTTCGAGATCAAGTCCAGCGTCGGTTCTAAGCGCACCGCCGATGGATCCGAGTTCGAATCCCCTAAAAAGAAAAGAAAGGTGGGATCCGACTACAAAGACCATGACGGTGATTATAAAGATCATGATATCGATTACAAGGATGACGATGACAAGGGATCCAAAAGAACTGGAAGCGGAGCTACTAACTTCAGCCTGCTGAAGCAGGCTGGCGACGTGGAGGAGAACCCTGGACCTGTTAACATGGTGAGCAAGGGCGAGGAGGATAACATGGCCATCATCAAGGAGTTCATGCGCTTCAAGGTGCACATGGAGGGCTCCGTGAACGGCCACGAGTTCGAGATCGAGGGCGAGGGCGAGGGCCGCCCCTACGAGGGCACCCAGACCGCCAAGCTGAAGGTGACCAAGGGTGGCCCCCTGCCCTTCGCCTGGGACATCCTGTCCCCTCAGTTCATGTACGGCTCCAAGGCCTACGTGAAGCACCCCGCCGACATCCCCGACTACTTGAAGCTGTCCTTCCCCGAGGGCTTCAAGTGGGAGCGCGTGATGAACTTCGAGGACGGCGGCGTGGTGACCGTGACCCAGGACTCCTCCCTGCAGGACGGCGAGTTCATCTACAAGGTGAAGCTGCGCGGCACCAACTTCCCCTCCGACGGCCCCGTAATGCAGAAGAAGACCATGGGCTGGGAGGCCTCCTCCGAGCGGATGTACCCCGAGGACGGCGCCCTGAAGGGCGAGATCAAGCAGAGGCTGAAGCTGAAGGACGGCGGCCACTACGACGCTGAGGTCAAGACCACCTACAAGGCCAAGAAGCCCGTGCAGCTGCCCGGCGCCTACAACGTCAACATCAAGTTGGACATCACCTCCCACAACGAGGACTACACCATCGTGGAACAGTACGAACGCGCCGAGGGCCGCCACTCCACCGGCGGCATGGACGAGCTGTACAAGTAAGCTCGAGCCTAGAGGGCCCGTTTAAACCCGCTGATCAGCCTCGACTGTGCCTTCTAGTTGCCAGCCATCTGTTGTTTGCCCCTCCCCCGTGCCTTCCTTGACCCTGGAAGGTGCCACTCCCACTGTCCTTTCCTAATAAAATGAGGAAATTGCATCGCATTGTCTGAGTAGGTGTCATTCTATTCTGGGGGGTGGGGTGGGGCAGGACAGCAAGGGGGAGGATTGGGAAGACAATAGCAGGCATGCTGGGGATGCGGTGGGCTCTATGGTACCGTCGACCTCTAGCTAGAGCTTGGCGTAATCATGGTCATAGCTGTTTCCTGTGTGAAATTGTTATCCGCTCACAATTCCACACAACATACGAGCCGGAAGCATAAAGTGTAAAGCCTGGGGTGCCTAATGAGTGAGCTAACTCACATTAATTGCGTTGCGCTCACTGCCCGCTTTCCAGTCGGGAAACCTGTCGTGCCAGCTGCATTAATGAATCGGCCAACGCGCGGGGAGAGGCGGTTTGCGTATTGGGCGCTCTTCCGCTTCCTCGCTCACTGACTCGCTGCGCTCGGTCGTTCGGCTGCGGCGAGCGGTATCAGCTCACTCAAAGGCGGTAATACGGTTATCCACAGAATCAGGGGATAACGCAGGAAAGAACATGTGAGCAAAAGGCCAGCAAAAGGCCAGGAACCGTAAAAAGGCCGCGTTGCTGGCGTTTTTCCATAGGCTCCGCCCCCCTGACGAGCATCACAAAAATCGACGCTCAAGTCAGAGGTGGCGAAACCCGACAGGACTATAAAGATACCAGGCGTTTCCCCCTGGAAGCTCCCTCGTGCGCTCTCCTGTTCCGACCCTGCCGCTTACCGGATACCTGTCCGCCTTTCTCCCTTCGGGAAGCGTGGCGCTTTCTCATAGCTCACGCTGTAGGTATCTCAGTTCGGTGTAGGTCGTTCGCTCCAAGCTGGGCTGTGTGCACGAACCCCCCGTTCAGCCCGACCGCTGCGCCTTATCCGGTAACTATCGTCTTGAGTCCAACCCGGTAAGACACGACTTATCGCCACTGGCAGCAGCCACTGGTAACAGGATTAGCAGAGCGAGGTATGTAGGCGGTGCTACAGAGTTCTTGAAGTGGTGGCCTAACTACGGCTACACTAGAAGAACAGTATTTGGTATCTGCGCTCTGCTGAAGCCAGTTACCTTCGGAAAAAGAGTTGGTAGCTCTTGATCCGGCAAACAAACCACCGCTGGTAGCGGTTTTTTTGTTTGCAAGCAGCAGATTACGCGCAGAAAAAAAGGATCTCAAGAAGATCCTTTGATCTTTTCTACGGGGTCTGACGCTCAGTGGAACGAAAACTCACGTTAAGGGATTTTGGTCATGAGATTATCAAAAAGGATCTTCACCTAGATCCTTTTAAATTAAAAATGAAGTTTTAAATCAATCTAAAGTATATATGAGTAAACTTGGTCTGACAGTTACCAATGCTTAATCAGTGAGGCACCTATCTCAGCGATCTGTCTATTTCGTTCATCCATAGTTGCCTGACTCCCCGTCGTGTAGATAACTACGATACGGGAGGGCTTACCATCTGGCCCCAGTGCTGCAATGATACCGCGAGACCCACGCTCACCGGCTCCAGATTTATCAGCAATAAACCAGCCAGCCGGAAGGGCCGAGCGCAGAAGTGGTCCTGCAACTTTATCCGCCTCCATCCAGTCTATTAATTGTTGCCGGGAAGCTAGAGTAAGTAGTTCGCCAGTTAATAGTTTGCGCAACGTTGTTGCCATTGCTACAGGCATCGTGGTGTCACGCTCGTCGTTTGGTATGGCTTCATTCAGCTCCGGTTCCCAACGATCAAGGCGAGTTACATGATCCCCCATGTTGTGCAAAAAAGCGGTTAGCTCCTTCGGTCCTCCGATCGTTGTCAGAAGTAAGTTGGCCGCAGTGTTATCACTCATGGTTATGGCAGCACTGCATAATTCTCTTACTGTCATGCCATCCGTAAGATGCTTTTCTGTGACTGGTGAGTACTCAACCAAGTCATTCTGAGAATAGTGTATGCGGCGACCGAGTTGCTCTTGCCCGGCGTCAATACGGGATAATACCGCGCCACATAGCAGAACTTTAAAAGTGCTCATCATTGGAAAACGTTCTTCGGGGCGAAAACTCTCAAGGATCTTACCGCTGTTGAGATCCAGTTCGATGTAACCCACTCGTGCACCCAACTGATCTTCAGCATCTTTTACTTTCACCAGCGTTTCTGGGTGA

ZFNGSA9075-F: GCAAAAACAGGAAGGCAAAATGCCGCAAAAAAGGGAATAAGGGCGACACGGAAATGTTGAATACTCATACTCTTCCTTTTTCAATATTATTGAAGCATTTATCAGGGTTATTGTCTCATGAGCGGATACATATTTGAATGTATTTAGAAAAATAAACAAATAGGGGTTCCGCGCACATTTCCCCGAAAAGTGCCACCTGACGTCGACGGATCGGGAGATCTCCCGATCCCCTATGGTGCACTCTCAGTACAATCTGCTCTGATGCCGCATAGTTAAGCCAGTATCTGCTCCCTGCTTGTGTGTTGGAGGTCGCTGAGTAGTGCGCGAGCAAAATTTAAGCTACAACAAGGCAAGGCTTGACCGACAATTGCATGAAGAATCTGCTTAGGGTTAGGCGTTTTGCGCTGCTTCGCGATGTACGGGCCAGATATACGCGTTGACATTGATTATTGACTAGTTATTAATAGTAATCAATTACGGGGTCATTAGTTCATAGCCCATATATGGAGTTCCGCGTTACATAACTTACGGTAAATGGCCCGCCTGGCTGACCGCCCAACGACCCCCGCCCATTGACGTCAATAATGACGTATGTTCCCATAGTAACGCCAATAGGGACTTTCCATTGACGTCAATGGGTGGAGTATTTACGGTAAACTGCCCACTTGGCAGTACATCAAGTGTATCATATGCCAAGTACGCCCCCTATTGACGTCAATGACGGTAAATGGCCCGCCTGGCATTATGCCCAGTACATGACCTTATGGGACTTTCCTACTTGGCAGTACATCTACGTATTAGTCATCGCTATTACCATGGTGATGCGGTTTTGGCAGTACATCAATGGGCGTGGATAGCGGTTTGACTCACGGGGATTTCCAAGTCTCCACCCCATTGACGTCAATGGGAGTTTGTTTTGGCACCAAAATCAACGGGACTTTCCAAAATGTCGTAACAACTCCGCCCCATTGACGCAAATGGGCGGTAGGCGTGTACGGTGGGAGGTCTATATAAGCAGAGCTCTCTGGCTAACTAGAGAACCCACTGCTTACTGGCTTATCGAAATTAATACGACTCACTATAGGGAGACCCAAGCTGGCTAGCGTTTAAACTTAAGCTTGCCACCATGGCGCCTAAGAAAAAGAGAAAGGTGGGCATCCACGGCGTGCCTGCCGCTATGGCCGAGCGCCCCTTTCAATGTAGAATCTGCATGCGCAATTTCAGCAGAAGCGACAACCTCTCCGAGCACATCAGAACACACACCGGCGAGAAGCCATTTGCCTGCGACATCTGTGGCAGAAAGTTCGCTAAACGGTGCAACCTGAGATGCCACACAAAGATTCACACCCACCCCAGAGCCCCTATCCCCAAGCCTTTTCAGTGCAGAATCTGCATGCGGAATTTCTCTCAGAGCTCTGATCTGAGCAGACATATCCGGACCCACACTGGAGAGAAACCTTTCGCCTGTGATATCTGCGGCAGAAAGTTCGCCCAGTGGAGCACCCGGAAGCGGCACACCAAGATTCACACCGGCGAGAAGCCTTTCCAGTGTAGAATCTGCATGCGGAAATTCGCCCAATCTGGAGATCTGACAAGACACACAAAGATCCACCTGCGGCAGAAGGACGCTGCCAGAGGCAGCGGAATGGACGCCAAGAGCCTGACCGCCTGGTCCATGCTAGAACCAGGTGAAAAGCCATATAAGTGTCCTGAATGCGGAAAGTCCTTTTCTCGGAAGGACGTGCGGATCACCCATCAACGCACACACCCCGGCGAGAAGCCCTACAAGTGCCCAGAATGTGGCAAAAGCTTCTCCAGATCTGACACCCTGTCCAACCACCAGAGAACACATCCTGGCGAGAAGCCTTACAAATGCCCTGAGTGCGGCAAGTCCTTCTCCCGGTCCGACAACCTGAGAGAGCACCAGCGGACCCACCCTGGCGAAAAGCCTTACAAGTGCCCTGAGTGCGGCAAGTCTTTCAGCCAGTCCTCTGATCTGTCTAGACACCAGAGAACCCACACCGGCAAGAAGACCCAACTAGTGAAGAGTGAACTAGAAGAAAAGAAGTCCGAGCTGAGACACAAGCTGAAGTACGTGCCTCACGAGTACATCGAACTGATCGAGATCGCCCGGAACTCCACACAAGACAGAATCCTGGAAATGAAAGTGATGGAATTCTTCATGAAAGTCTACGGCTATAGAGGCAAGCACCTGGGAGGCTCCAGAAAGCCTGATGGCGCCATCTACACCGTGGGCTCTCCAATCGACTACGGAGTGATCGTGGACACCAAGGCCTACTCCGGCGGCTACAACCTGCCTATTGGCCAGGCCGACGAGATGCAGAGATACGTCAAAGAGAACCAGACCAGAAACAAGCACATCAATCCTAACGAGTGGTGGAAGGTGTACCCCAGCTCCGTGACCGAGTTTAAGTTCCTGTTCGTGTCTGGCCATTTTAAGGGCAACTACAAGGCTCAGCTGACCCGGCTGAATCGGAAGACCAACTGCAACGGCGCTGTGCTGTCTGTGGAAGAGCTGCTGATCGGCGGCGAGATGATCAAGGCTGGCACCCTGACACTCGAGGAAGTGCGGCGCAAGTTCAACAACGGAGAAATCAACTTCGAACGGGAAATCCACCAGGAGACACATCCTGACAGCGAGACAGCCTTCGAGATCAAGTCCAGCGTCGGTTCTAAGCGCACCGCCGATGGATCCGAGTTCGAATCCCCTAAAAAGAAAAGAAAGGTGGGATCCGACTACAAAGACCATGACGGTGATTATAAAGATCATGATATCGATTACAAGGATGACGATGACAAGGGATCCAAAAGAACTGGAAGCGGAGCTACTAACTTCAGCCTGCTGAAGCAGGCTGGCGACGTGGAGGAGAACCCTGGACCTGTTAACATGGTGAGCAAGGGCGAGGAGGATAACATGGCCATCATCAAGGAGTTCATGCGCTTCAAGGTGCACATGGAGGGCTCCGTGAACGGCCACGAGTTCGAGATCGAGGGCGAGGGCGAGGGCCGCCCCTACGAGGGCACCCAGACCGCCAAGCTGAAGGTGACCAAGGGTGGCCCCCTGCCCTTCGCCTGGGACATCCTGTCCCCTCAGTTCATGTACGGCTCCAAGGCCTACGTGAAGCACCCCGCCGACATCCCCGACTACTTGAAGCTGTCCTTCCCCGAGGGCTTCAAGTGGGAGCGCGTGATGAACTTCGAGGACGGCGGCGTGGTGACCGTGACCCAGGACTCCTCCCTGCAGGACGGCGAGTTCATCTACAAGGTGAAGCTGCGCGGCACCAACTTCCCCTCCGACGGCCCCGTAATGCAGAAGAAGACCATGGGCTGGGAGGCCTCCTCCGAGCGGATGTACCCCGAGGACGGCGCCCTGAAGGGCGAGATCAAGCAGAGGCTGAAGCTGAAGGACGGCGGCCACTACGACGCTGAGGTCAAGACCACCTACAAGGCCAAGAAGCCCGTGCAGCTGCCCGGCGCCTACAACGTCAACATCAAGTTGGACATCACCTCCCACAACGAGGACTACACCATCGTGGAACAGTACGAACGCGCCGAGGGCCGCCACTCCACCGGCGGCATGGACGAGCTGTACAAGTAAGCTCGAGCCTAGAGGGCCCGTTTAAACCCGCTGATCAGCCTCGACTGTGCCTTCTAGTTGCCAGCCATCTGTTGTTTGCCCCTCCCCCGTGCCTTCCTTGACCCTGGAAGGTGCCACTCCCACTGTCCTTTCCTAATAAAATGAGGAAATTGCATCGCATTGTCTGAGTAGGTGTCATTCTATTCTGGGGGGTGGGGTGGGGCAGGACAGCAAGGGGGAGGATTGGGAAGACAATAGCAGGCATGCTGGGGATGCGGTGGGCTCTATGGTACCGTCGACCTCTAGCTAGAGCTTGGCGTAATCATGGTCATAGCTGTTTCCTGTGTGAAATTGTTATCCGCTCACAATTCCACACAACATACGAGCCGGAAGCATAAAGTGTAAAGCCTGGGGTGCCTAATGAGTGAGCTAACTCACATTAATTGCGTTGCGCTCACTGCCCGCTTTCCAGTCGGGAAACCTGTCGTGCCAGCTGCATTAATGAATCGGCCAACGCGCGGGGAGAGGCGGTTTGCGTATTGGGCGCTCTTCCGCTTCCTCGCTCACTGACTCGCTGCGCTCGGTCGTTCGGCTGCGGCGAGCGGTATCAGCTCACTCAAAGGCGGTAATACGGTTATCCACAGAATCAGGGGATAACGCAGGAAAGAACATGTGAGCAAAAGGCCAGCAAAAGGCCAGGAACCGTAAAAAGGCCGCGTTGCTGGCGTTTTTCCATAGGCTCCGCCCCCCTGACGAGCATCACAAAAATCGACGCTCAAGTCAGAGGTGGCGAAACCCGACAGGACTATAAAGATACCAGGCGTTTCCCCCTGGAAGCTCCCTCGTGCGCTCTCCTGTTCCGACCCTGCCGCTTACCGGATACCTGTCCGCCTTTCTCCCTTCGGGAAGCGTGGCGCTTTCTCATAGCTCACGCTGTAGGTATCTCAGTTCGGTGTAGGTCGTTCGCTCCAAGCTGGGCTGTGTGCACGAACCCCCCGTTCAGCCCGACCGCTGCGCCTTATCCGGTAACTATCGTCTTGAGTCCAACCCGGTAAGACACGACTTATCGCCACTGGCAGCAGCCACTGGTAACAGGATTAGCAGAGCGAGGTATGTAGGCGGTGCTACAGAGTTCTTGAAGTGGTGGCCTAACTACGGCTACACTAGAAGAACAGTATTTGGTATCTGCGCTCTGCTGAAGCCAGTTACCTTCGGAAAAAGAGTTGGTAGCTCTTGATCCGGCAAACAAACCACCGCTGGTAGCGGTTTTTTTGTTTGCAAGCAGCAGATTACGCGCAGAAAAAAAGGATCTCAAGAAGATCCTTTGATCTTTTCTACGGGGTCTGACGCTCAGTGGAACGAAAACTCACGTTAAGGGATTTTGGTCATGAGATTATCAAAAAGGATCTTCACCTAGATCCTTTTAAATTAAAAATGAAGTTTTAAATCAATCTAAAGTATATATGAGTAAACTTGGTCTGACAGTTACCAATGCTTAATCAGTGAGGCACCTATCTCAGCGATCTGTCTATTTCGTTCATCCATAGTTGCCTGACTCCCCGTCGTGTAGATAACTACGATACGGGAGGGCTTACCATCTGGCCCCAGTGCTGCAATGATACCGCGAGACCCACGCTCACCGGCTCCAGATTTATCAGCAATAAACCAGCCAGCCGGAAGGGCCGAGCGCAGAAGTGGTCCTGCAACTTTATCCGCCTCCATCCAGTCTATTAATTGTTGCCGGGAAGCTAGAGTAAGTAGTTCGCCAGTTAATAGTTTGCGCAACGTTGTTGCCATTGCTACAGGCATCGTGGTGTCACGCTCGTCGTTTGGTATGGCTTCATTCAGCTCCGGTTCCCAACGATCAAGGCGAGTTACATGATCCCCCATGTTGTGCAAAAAAGCGGTTAGCTCCTTCGGTCCTCCGATCGTTGTCAGAAGTAAGTTGGCCGCAGTGTTATCACTCATGGTTATGGCAGCACTGCATAATTCTCTTACTGTCATGCCATCCGTAAGATGCTTTTCTGTGACTGGTGAGTACTCAACCAAGTCATTCTGAGAATAGTGTATGCGGCGACCGAGTTGCTCTTGCCCGGCGTCAATACGGGATAATACCGCGCCACATAGCAGAACTTTAAAAGTGCTCATCATTGGAAAACGTTCTTCGGGGCGAAAACTCTCAAGGATCTTACCGCTGTTGAGATCCAGTTCGATGTAACCCACTCGTGCACCCAACTGATCTTCAGCATCTTTTACTTTCACCAGCGTTTCTGGGTGA

CBE4max-SpRY base editor plasmid: ATATGCCAAGTACGCCCCCTATTGACGTCAATGACGGTAAATGGCCCGCCTGGCATTATGCCCAGTACATGACCTTATGGGACTTTCCTACTTGGCAGTACATCTACGTATTAGTCATCGCTATTACCATGGTGATGCGGTTTTGGCAGTACATCAATGGGCGTGGATAGCGGTTTGACTCACGGGGATTTCCAAGTCTCCACCCCATTGACGTCAATGGGAGTTTGTTTTGGCACCAAAATCAACGGGACTTTCCAAAATGTCGTAACAACTCCGCCCCATTGACGCAAATGGGCGGTAGGCGTGTACGGTGGGAGGTCTATATAAGCAGAGCTGGTTTAGTGAACCGTCAGATCCGCTAGAGATCCGCGGCCGCTAATACGACTCACTATAGGGAGAGCCGCCACCATGAAACGGACAGCCGACGGAAGCGAGTTCGAGTCACCAAAGAAGAAGCGGAAAGTCTCCTCAGAGACTGGGCCTGTCGCCGTCGATCCAACCCTGCGCCGCCGGATTGAACCTCACGAGTTTGAAGTGTTCTTTGACCCCCGGGAGCTGAGAAAGGAGACATGCCTGCTGTACGAGATCAACTGGGGAGGCAGGCACTCCATCTGGAGGCACACCTCTCAGAACACAAATAAGCACGTGGAGGTGAACTTCATCGAGAAGTTTACCACAGAGCGGTACTTCTGCCCCAATACCAGATGTAGCATCACATGGTTTCTGAGCTGGTCCCCTTGCGGAGAGTGTAGCAGGGCCATCACCGAGTTCCTGTCCAGATATCCACACGTGACACTGTTTATCTACATCGCCAGGCTGTATCACCACGCAGACCCAAGGAATAGGCAGGGCCTGCGCGATCTGATCAGCTCCGGCGTGACCATCCAGATCATGACAGAGCAGGAGTCCGGCTACTGCTGGCGGAACTTCGTGAATTATTCTCCTAGCAACGAGGCCCACTGGCCTAGGTACCCACACCTGTGGGTGCGCCTGTACGTGCTGGAGCTGTATTGCATCATCCTGGGCCTGCCCCCTTGTCTGAATATCCTGCGGAGAAAGCAGCCCCAGCTGACCTTCTTTACAATCGCCCTGCAGTCTTGTCACTATCAGAGGCTGCCACCCCACATCCTGTGGGCCACAGGCCTGAAGTCTGGAGGATCTAGCGGAGGATCCTCTGGCAGCGAGACACCAGGAACAAGCGAGTCAGCAACACCAGAGAGCAGTGGCGGCAGCAGCGGCGGCAGCGACAAGAAGTACAGCATCGGCCTGGCCATCGGCACCAACTCTGTGGGCTGGGCCGTGATCACCGACGAGTACAAGGTGCCCAGCAAGAAATTCAAGGTGCTGGGCAACACCGACCGGCACAGCATCAAGAAGAACCTGATCGGAGCCCTGCTGTTCGACAGCGGCGAAACAGCCGAGAGAACCCGGCTGAAGAGAACCGCCAGAAGAAGATACACCAGACGGAAGAACCGGATCTGCTATCTGCAAGAGATCTTCAGCAACGAGATGGCCAAGGTGGACGACAGCTTCTTCCACAGACTGGAAGAGTCCTTCCTGGTGGAAGAGGATAAGAAGCACGAGCGGCACCCCATCTTCGGCAACATCGTGGACGAGGTGGCCTACCACGAGAAGTACCCCACCATCTACCACCTGAGAAAGAAACTGGTGGACAGCACCGACAAGGCCGACCTGCGGCTGATCTATCTGGCCCTGGCCCACATGATCAAGTTCCGGGGCCACTTCCTGATCGAGGGCGACCTGAACCCCGACAACAGCGACGTGGACAAGCTGTTCATCCAGCTGGTGCAGACCTACAACCAGCTGTTCGAGGAAAACCCCATCAACGCCAGCGGCGTGGACGCCAAGGCCATCCTGTCTGCCAGACTGAGCAAGAGCAGACGGCTGGAAAATCTGATCGCCCAGCTGCCCGGCGAGAAGAAGAATGGCCTGTTCGGAAACCTGATTGCCCTGAGCCTGGGCCTGACCCCCAACTTCAAGAGCAACTTCGACCTGGCCGAGGATGCCAAACTGCAGCTGAGCAAGGACACCTACGACGACGACCTGGACAACCTGCTGGCCCAGATCGGCGACCAGTACGCCGACCTGTTTCTGGCCGCCAAGAACCTGTCCGACGCCATCCTGCTGAGCGACATCCTGAGAGTGAACACCGAGATCACCAAGGCCCCCCTGAGCGCCTCTATGATCAAGAGATACGACGAGCACCACCAGGACCTGACCCTGCTGAAAGCTCTCGTGCGGCAGCAGCTGCCTGAGAAGTACAAAGAGATTTTCTTCGACCAGAGCAAGAACGGCTACGCCGGCTACATTGACGGCGGAGCCAGCCAGGAAGAGTTCTACAAGTTCATCAAGCCCATCCTGGAAAAGATGGACGGCACCGAGGAACTGCTCGTGAAGCTGAACAGAGAGGACCTGCTGCGGAAGCAGCGGACCTTCGACAACGGCAGCATCCCCCACCAGATCCACCTGGGAGAGCTGCACGCCATTCTGCGGCGGCAGGAAGATTTTTACCCATTCCTGAAGGACAACCGGGAAAAGATCGAGAAGATCCTGACCTTCCGCATCCCCTACTACGTGGGCCCTCTGGCCAGGGGAAACAGCAGATTCGCCTGGATGACCAGAAAGAGCGAGGAAACCATCACCCCCTGGAACTTCGAGGAAGTGGTGGACAAGGGCGCTTCCGCCCAGAGCTTCATCGAGCGGATGACCAACTTCGATAAGAACCTGCCCAACGAGAAGGTGCTGCCCAAGCACAGCCTGCTGTACGAGTACTTCACCGTGTATAACGAGCTGACCAAAGTGAAATACGTGACCGAGGGAATGAGAAAGCCCGCCTTCCTGAGCGGCGAGCAGAAAAAGGCCATCGTGGACCTGCTGTTCAAGACCAACCGGAAAGTGACCGTGAAGCAGCTGAAAGAGGACTACTTCAAGAAAATCGAGTGCTTCGACTCCGTGGAAATCTCCGGCGTGGAAGATCGGTTCAACGCCTCCCTGGGCACATACCACGATCTGCTGAAAATTATCAAGGACAAGGACTTCCTGGACAATGAGGAAAACGAGGACATTCTGGAAGATATCGTGCTGACCCTGACACTGTTTGAGGACAGAGAGATGATCGAGGAACGGCTGAAAACCTATGCCCACCTGTTCGACGACAAAGTGATGAAGCAGCTGAAGCGGCGGAGATACACCGGCTGGGGCAGGCTGAGCCGGAAGCTGATCAACGGCATCCGGGACAAGCAGTCCGGCAAGACAATCCTGGATTTCCTGAAGTCCGACGGCTTCGCCAACAGAAACTTCATGCAGCTGATCCACGACGACAGCCTGACCTTTAAAGAGGACATCCAGAAAGCCCAGGTGTCCGGCCAGGGCGATAGCCTGCACGAGCACATTGCCAATCTGGCCGGCAGCCCCGCCATTAAGAAGGGCATCCTGCAGACAGTGAAGGTGGTGGACGAGCTCGTGAAAGTGATGGGCCGGCACAAGCCCGAGAACATCGTGATCGAAATGGCCAGAGAGAACCAGACCACCCAGAAGGGACAGAAGAACAGCCGCGAGAGAATGAAGCGGATCGAAGAGGGCATCAAAGAGCTGGGCAGCCAGATCCTGAAAGAACACCCCGTGGAAAACACCCAGCTGCAGAACGAGAAGCTGTACCTGTACTACCTGCAGAATGGGCGGGATATGTACGTGGACCAGGAACTGGACATCAACCGGCTGTCCGACTACGATGTGGACCATATCGTGCCTCAGAGCTTTCTGAAGGACGACTCCATCGACAACAAGGTGCTGACCAGAAGCGACAAGAACCGGGGCAAGAGCGACAACGTGCCCTCCGAAGAGGTCGTGAAGAAGATGAAGAACTACTGGCGGCAGCTGCTGAACGCCAAGCTGATTACCCAGAGAAAGTTCGACAATCTGACCAAGGCCGAGAGAGGCGGCCTGAGCGAACTGGATAAGGCCGGCTTCATCAAGAGACAGCTGGTGGAAACCCGGCAGATCACAAAGCACGTGGCACAGATCCTGGACTCCCGGATGAACACTAAGTACGACGAGAATGACAAGCTGATCCGGGAAGTGAAAGTGATCACCCTGAAGTCCAAGCTGGTGTCCGATTTCCGGAAGGATTTCCAGTTTTACAAAGTGCGCGAGATCAACAACTACCACCACGCCCACGACGCCTACCTGAACGCCGTCGTGGGAACCGCCCTGATCAAAAAGTACCCTAAGCTGGAAAGCGAGTTCGTGTACGGCGACTACAAGGTGTACGACGTGCGGAAGATGATCGCCAAGAGCGAGCAGGAAATCGGCAAGGCTACCGCCAAGTACTTCTTCTACAGCAACATCATGAACTTTTTCAAGACCGAGATTACCCTGGCCAACGGCGAGATCCGGAAGCGGCCTCTGATCGAGACAAACGGCGAAACCGGGGAGATCGTGTGGGATAAGGGCCGGGATTTTGCCACCGTGCGGAAAGTGCTGAGCATGCCCCAAGTGAATATCGTGAAAAAGACCGAGGTGCAGACAGGCGGCTTCAGCAAAGAGTCTATCAGACCCAAGAGGAACAGCGATAAGCTGATCGCCAGAAAGAAGGACTGGGACCCTAAGAAGTACGGCGGCTTCCTGTGGCCCACCGTGGCCTATTCTGTGCTGGTGGTGGCCAAAGTGGAAAAGGGCAAGTCCAAGAAACTGAAGAGTGTGAAAGAGCTGCTGGGGATCACCATCATGGAAAGAAGCAGCTTCGAGAAGAATCCCATCGACTTTCTGGAAGCCAAGGGCTACAAAGAAGTGAAAAAGGACCTGATCATCAAGCTGCCTAAGTACTCCCTGTTCGAGCTGGAAAACGGCCGGAAGAGAATGCTGGCCTCTGCCAAGCAGCTGCAGAAGGGAAACGAACTGGCCCTGCCCTCCAAATATGTGAACTTCCTGTACCTGGCCAGCCACTATGAGAAGCTGAAGGGCTCCCCCGAGGATAATGAGCAGAAACAGCTGTTTGTGGAACAGCACAAGCACTACCTGGACGAGATCATCGAGCAGATCAGCGAGTTCTCCAAGAGAGTGATCCTGGCCGACGCTAATCTGGACAAAGTGCTGTCCGCCTACAACAAGCACCGGGATAAGCCCATCAGAGAGCAGGCCGAGAATATCATCCACCTGTTTACCCTGACCAGACTGGGAGCCCCTAGAGCCTTCAAGTACTTTGACACCACCATCGACCCCAAGCAGTACAGAAGCACCAAAGAGGTGCTGGACGCCACCCTGATCCACCAGAGCATCACCGGCCTGTACGAGACACGGATCGACCTGTCTCAGCTGGGAGGCGATTCAGGCGGATCTACTAATCTGAGCGACATCATTGAGAAGGAGACTGGGAAACAGCTGGTCATTCAGGAGTCCATCCTGATGCTGCCTGAGGAGGTGGAGGAAGTGATCGGCAACAAGCCAGAGTCTGACATCCTGGTGCACACCGCCTACGACGAGTCCACAGATGAGAATGTGATGCTGCTGACCTCTGACGCCCCCGAGTATAAGCCTTGGGCCCTGGTCATCCAGGATTCTAACGGCGAGAATAAGATCAAGATGCTGAGCGGAGGATCCGGAGGATCTGGAGGCAGCACCAACCTGTCTGACATCATCGAGAAGGAGACAGGCAAGCAGCTGGTCATCCAGGAGAGCATCCTGATGCTGCCCGAAGAAGTCGAAGAAGTGATCGGAAACAAGCCTGAGAGCGATATCCTGGTCCATACCGCCTACGACGAGAGTACCGACGAAAATGTGATGCTGCTGACATCCGACGCCCCAGAGTATAAGCCCTGGGCTCTGGTCATCCAGGATTCCAACGGAGAGAACAAAATCAAAATGCTGTCTGGCGGCTCAAAAAGAACCGCCGACGGCAGCGAATTCGAGCCCAAGAAGAAGAGGAAAGTCTAAGCTAGCGGCAGCGGCTAAAAACCCGCTGATCAGCCTCGACTGTGCCTTCTAGTTGCCAGCCATCTGTTGTTTGCCCCTCCCCCGTGCCTTCCTTGACCCTGGAAGGTGCCACTCCCACTGTCCTTTCCTAATAAAATGAGGAAATTGCATCGCATTGTCTGAGTAGGTGTCATTCTATTCTGGGGGGTGGGGTGGGGCAGGACAGCAAGGGGGAGGATTGGGAAGACAATAGCAGGCATGCTGGGGATGCGGTGGGCTCTATGGCTTCTGAGGCGGAAAGAACCAGCTGGGGCTCGATACCGTCGACCTCTAGCTAGAGCTTGGCGTAATCATGGTCATAGCTGTTTCCTGTGTGAAATTGTTATCCGCTCACAATTCCACACAACATACGAGCCGGAAGCATAAAGTGTAAAGCCTAGGGTGCCTAATGAGTGAGCTAACTCACATTAATTGCGTTGCGCTCACTGCCCGCTTTCCAGTCGGGAAACCTGTCGTGCCAGCTGCATTAATGAATCGGCCAACGCGCGGGGAGAGGCGGTTTGCGTATTGGGCGCTCTTCCGCTTCCTCGCTCACTGACTCGCTGCGCTCGGTCGTTCGGCTGCGGCGAGCGGTATCAGCTCACTCAAAGGCGGTAATACGGTTATCCACAGAATCAGGGGATAACGCAGGAAAGAACATGTGAGCAAAAGGCCAGCAAAAGGCCAGGAACCGTAAAAAGGCCGCGTTGCTGGCGTTTTTCCATAGGCTCCGCCCCCCTGACGAGCATCACAAAAATCGACGCTCAAGTCAGAGGTGGCGAAACCCGACAGGACTATAAAGATACCAGGCGTTTCCCCCTGGAAGCTCCCTCGTGCGCTCTCCTGTTCCGACCCTGCCGCTTACCGGATACCTGTCCGCCTTTCTCCCTTCGGGAAGCGTGGCGCTTTCTCATAGCTCACGCTGTAGGTATCTCAGTTCGGTGTAGGTCGTTCGCTCCAAGCTGGGCTGTGTGCACGAACCCCCCGTTCAGCCCGACCGCTGCGCCTTATCCGGTAACTATCGTCTTGAGTCCAACCCGGTAAGACACGACTTATCGCCACTGGCAGCAGCCACTGGTAACAGGATTAGCAGAGCGAGGTATGTAGGCGGTGCTACAGAGTTCTTGAAGTGGTGGCCTAACTACGGCTACACTAGAAGAACAGTATTTGGTATCTGCGCTCTGCTGAAGCCAGTTACCTTCGGAAAAAGAGTTGGTAGCTCTTGATCCGGCAAACAAACCACCGCTGGTAGCGGTGGTTTTTTTGTTTGCAAGCAGCAGATTACGCGCAGAAAAAAAGGATCTCAAGAAGATCCTTTGATCTTTTCTACGGGGTCTGACGCTCAGTGGAACGAAAACTCACGTTAAGGGATTTTGGTCATGAGATTATCAAAAAGGATCTTCACCTAGATCCTTTTAAATTAAAAATGAAGTTTTAAATCAATCTAAAGTATATATGAGTAAACTTGGTCTGACAGTTACCAATGCTTAATCAGTGAGGCACCTATCTCAGCGATCTGTCTATTTCGTTCATCCATAGTTGCCTGACTCCCCGTCGTGTAGATAACTACGATACGGGAGGGCTTACCATCTGGCCCCAGTGCTGCAATGATACCGCGAGACCCACGCTCACCGGCTCCAGATTTATCAGCAATAAACCAGCCAGCCGGAAGGGCCGAGCGCAGAAGTGGTCCTGCAACTTTATCCGCCTCCATCCAGTCTATTAATTGTTGCCGGGAAGCTAGAGTAAGTAGTTCGCCAGTTAATAGTTTGCGCAACGTTGTTGCCATTGCTACAGGCATCGTGGTGTCACGCTCGTCGTTTGGTATGGCTTCATTCAGCTCCGGTTCCCAACGATCAAGGCGAGTTACATGATCCCCCATGTTGTGCAAAAAAGCGGTTAGCTCCTTCGGTCCTCCGATCGTTGTCAGAAGTAAGTTGGCCGCAGTGTTATCACTCATGGTTATGGCAGCACTGCATAATTCTCTTACTGTCATGCCATCCGTAAGATGCTTTTCTGTGACTGGTGAGTACTCAACCAAGTCATTCTGAGAATAGTGTATGCGGCGACCGAGTTGCTCTTGCCCGGCGTCAATACGGGATAATACCGCGCCACATAGCAGAACTTTAAAAGTGCTCATCATTGGAAAACGTTCTTCGGGGCGAAAACTCTCAAGGATCTTACCGCTGTTGAGATCCAGTTCGATGTAACCCACTCGTGCACCCAACTGATCTTCAGCATCTTTTACTTTCACCAGCGTTTCTGGGTGAGCAAAAACAGGAAGGCAAAATGCCGCAAAAAAGGGAATAAGGGCGACACGGAAATGTTGAATACTCATACTCTTCCTTTTTCAATATTATTGAAGCATTTATCAGGGTTATTGTCTCATGAGCGGATACATATTTGAATGTATTTAGAAAAATAAACAAATAGGGGTTCCGCGCACATTTCCCCGAAAAGTGCCACCTGACGTCGACGGATCGGGAGATCGATCTCCCGATCCCCTAGGGTCGACTCTCAGTACAATCTGCTCTGATGCCGCATAGTTAAGCCAGTATCTGCTCCCTGCTTGTGTGTTGGAGGTCGCTGAGTAGTGCGCGAGCAAAATTTAAGCTACAACAAGGCAAGGCTTGACCGACAATTGCATGAAGAATCTGCTTAGGGTTAGGCGTTTTGCGCTGCTTCGCGATGTACGGGCCAGATATAGGTACCCATTAATTAAATAGTAATCAATTACGGGGTCATTAGTTCATAGCCCATATATGGAGTTCCGCGTTACATAACTTACGGTAAATGGCCCGCCTGGCTGACCGCCCAACGACCCCCGCCCATTGACGTCAATAATGACGTATGTTCCCATAGTAACGCCAATAGGGACTTTCCATTGACGTCAATGGGTGGAGTATTTACGGTAAACTGCCCACTTGGCAGTACATCAAGTGTATCATATGCCAAGTCCGCCCCCTATTGACGTCAATGACGGTAAATGGCCCGCCTGGCATTATGCCCAGTACATGACCTTACGGGACTTTCCTACTTGGCAGTACATCTACGTATTAGTCATCGCTATTACCATGGTGATGCGGTTTTGGCAGTACACCAATGGGCGTGGATAGCGGTTTGACTCACGGGGATTTCCAAGTCTCCACCCCATTGACGTCAATGGGAGTTTGTTTTGGCACCAAAATCAACGGGACTTTCCAAAATGTCGTAATAACCCCGCCCCGTTGACGCAAATGGGCGGTAGGCGTGTACGGTGGGAGGTCTATATAAGCAGAGGTCGTTTAGTGAACCGTCAGATCACGCGTGCCACCATGGTGAGCAAGGGCGAGGAGGATAACATGGCCATCATCAAGGAGTTCATGCGCTTCAAGGTGCACATGGAGGGCTCCGTGAACGGCCACGAGTTCGAGATCGAGGGCGAGGGCGAGGGCCGCCCCTACGAGGGCACCCAGACCGCCAAGCTGAAGGTGACCAAGGGTGGCCCCCTGCCCTTCGCCTGGGACATCCTGTCCCCTCAGTTCATGTACGGCTCCAAGGCCTACGTGAAGCACCCCGCCGACATCCCCGACTACTTGAAGCTGTCCTTCCCCGAGGGCTTCAAGTGGGAGCGCGTGATGAACTTCGAGGACGGCGGCGTGGTGACCGTGACCCAGGACTCCTCCCTGCAGGACGGCGAGTTCATCTACAAGGTGAAGCTGCGCGGCACCAACTTCCCCTCCGACGGCCCCGTAATGCAGAAGAAGACCATGGGCTGGGAGGCCTCCTCCGAGCGGATGTACCCCGAGGACGGCGCCCTGAAGGGCGAGATCAAGCAGAGGCTGAAGCTGAAGGACGGCGGCCACTACGACGCTGAGGTCAAGACCACCTACAAGGCCAAGAAGCCCGTGCAGCTGCCCGGCGCCTACAACGTCAACATCAAGTTGGACATCACCTCCCACAACGAGGACTACACCATCGTGGAACAGTACGAACGCGCCGAGGGCCGCCACTCCACCGGCGGCATGGACGAGCTGTACAAGTAAAATCAACCTCTGGATTACAAAATTTGTGAAAGATTGACTGGTATTCTTAACTATGTTGCTCCTTTTACGCTATGTGGATACGCTGCTTTAATGCCTTTGTATCATGCTATTGCTTCCCGTATGGCTTTCATTTTCTCCTCCTTGTATAAATCCTGGTTGCTGTCTCTTTATGAGGAGTTGTGGCCCGTTGTCAGGCAACGTGGCGTGGTGTGCACTGTGTTTGCTGACGCAACCCCCACTGGTTGGGGCATTGCCACCACCTGTCAGCTCCTTTCCGGGACTTTCGCTTTCCCCCTCCCTATTGCCACGGCGGAACTCATCGCCGCCTGCCTTGCCCGCTGCTGGACAGGGGCTCGGCTGTTGGGCACTGACAATTCCGTGGTGTTGTCGGGGAAGCTGACGTCCTTTCCATGGCTGCTCGCCTGTGTTGCCACCTGGATTCTGCGCGGGACGTCCTTCTGCTACGTCCCTTCGGCCCTCAATCCAGCGGACCTTCCTTCCCGCGGCCTGCTGCCGGCTCTGCGGCCTCTTCCGCGTCTCCGCCTTCGCCCTCAGACGAGTCGGATCTCCCTTTGGCCGCCTCCCCGCCACCGGTTAGGGGCCCGTTTAAACCCGCTGATCAGCCTCGACTGTGCCTTCTAGTTGCCAGCCATCTGTTGTTTGCCCCTCCCCCGTGCCTTCCTTGACCCTGGAAGGTGCCACTCCCACTGTCCTTTCCTAATAAAATGAGGAAATTGCATCGCATTGTCTGAGTAGGTGTCATTCTATTCTGGGGGGTGGGGTGGGGCAGGACAGCAAGGGGGAGGATTGGGAAGACAATAGCAGGCATGCTGGGGATGCGGTGGGCTCTATGGCGGCGCGCCTAAAAGTTTTGTTACTGACATTGATTATTGACTAGTTATTAATAGTAATCAATTACGGGGTCATTAGTTCATAGCCCATATATGGAGTTCCGCGTTACATAACTTACGGTAAATGGCCCGCCTGGCTGACCGCCCAACGACCCCCGCCCATTGACGTCAATAATGACGTATGTTCCCATAGTAACGCCAATAGGGACTTTCCATTGACGTCAATGGGTGGAGTATTTACGGTAAACTGCCCACTTGGCAGTACATCAAGTGTATC

Cas9 plasmid: GACGGATCGGGAGATCTCCCGATCCCCTATGGTCGACTCTCAGTACAATCTGCTCTGATGCCGCATAGTTAAGCCAGTATCTGCTCCCTGCTTGTGTGTTGGAGGTCGCTGAGTAGTGCGCGAGCAAAATTTAAGCTACAACAAGGCAAGGCTTGACCGACAATTGCATGAAGAATCTGCTTAGGGTTAGGCGTTTTGCGCTGCTTCGCGATGTACGGGCCAGATATACGCGTTGACATTGATTATTGACTAGTTATTAATAGTAATCAATTACGGGGTCATTAGTTCATAGCCCATATATGGAGTTCCGCGTTACATAACTTACGGTAAATGGCCCGCCTGGCTGACCGCCCAACGACCCCCGCCCATTGACGTCAATAATGACGTATGTTCCCATAGTAACGCCAATAGGGACTTTCCATTGACGTCAATGGGTGGAGTATTTACGGTAAACTGCCCACTTGGCAGTACATCAAGTGTATCATATGCCAAGTACGCCCCCTATTGACGTCAATGACGGTAAATGGCCCGCCTGGCATTATGCCCAGTACATGACCTTATGGGACTTTCCTACTTGGCAGTACATCTACGTATTAGTCATCGCTATTACCATGGTGATGCGGTTTTGGCAGTACATCAATGGGCGTGGATAGCGGTTTGACTCACGGGGATTTCCAAGTCTCCACCCCATTGACGTCAATGGGAGTTTGTTTTGGCACCAAAATCAACGGGACTTTCCAAAATGTCGTAACAACTCCGCCCCATTGACGCAAATGGGCGGTAGGCGTGTACGGTGGGAGGTCTATATAAGCAGAGCTCTCTGGCTAACTAGAGAACCCACTGCTTACTGGCTTATCGAAATTAATACGACTCACTATAGGGAGACCCAAGCTGGCTAGCACCATGGacaagaaatactctattggactggatatcgggacaaactccgttggctgggccgtcataaccgacgagtataaggtgccaagcaagaaattcaaggtgctgggtaatactgaccgccattcaatcaagaagaacctgatcggagcactcctcttcgactccggtgaaaccgctgaagctactcggctgaagcggaccgcaaggcggagatacacccgccgcaagaatcggatatgttatctgcaagagatctttagcaacgaaatggctaaggtggacgactccttctttcaccgcctggaagagagctttctggtggaggaggataagaaacacgagaggcaccctatattcggaaatatcgtggatgaggtggcttaccatgaaaagtatcctacaatctaccatctgaggaagaagctggtggacagcaccgataaagcagacctgaggctcatctatctggccctggctcatatgataaagtttagaggacactttctgatcgagggcgacctgaatcccgataattccgatgtggataaactcttcattcaactggtgcagacatataaccaactgttcgaggagaatcccataaacgcttctggtgtggatgccaaggctattctgtccgctcggctgtccaagtcacgcagactggagaatctgattgcccaactgccaggagaaaagaagaacggcctgtttgggaacctcatcgccctgagcctgggcctgacacctaacttcaagtccaattttgatctggccgaagatgctaaactccagctctccaaggacacctatgacgatgatctggacaacctgctcgcacagataggcgaccagtacgccgatctctttctggctgctaagaatctctccgacgccattctgctgagcgacatactccgggtcaacactgagatcaccaaagcacctctgagcgcctccatgataaaacgctatgatgaacaccatcaagacctgactctgctcaaagccctcgtgaggcaacagctgccagagaagtacaaagagatattcttcgaccagagcaagaatggatatgccggatacatcgatggcggagcatcacaggaagaattttacaagttcatcaaaccaatcctcgagaagatggacggtactgaagagctgctggtgaagctgaacagggaggacctgctgaggaagcagaggacctttgataatggctccattccacatcagatacacctgggagagctgcatgcaatcctccgcaggcaggaggatttctatcctttcctgaaggataaccgggagaagatagagaagatcctgaccttcaggatcccttattacgtcggccctctggctagaggcaactcccgcttcgcttggatgaccaggaaatctgaggagacaattactccttggaacttcgaagaggtcgtggataagggcgcaagcgcccagtcattcatcgaacggatgaccaatttcgataagaacctgcccaacgagaaggtcctgcccaaacattcactcctgtacgagtatttcaccgtctataacgagctgactaaagtgaagtacgtgaccgagggcatgaggaagcctgccttcctgtccggagagcagaagaaggctatcgttgatctgctcttcaagactaatagaaaggtgacagtgaagcagctcaaggaggattactttaagaagatcgaatgctttgactcagtggaaatctctggcgtggaggaccgctttaatgccagcctgggcacttaccatgatctgctgaagataatcaaagacaaagatttcctcgataatgaggagaacgaggacatcctggaagatatcgtgctgaccctgactctgttcgaggatagagagatgatcgaagagcgcctgaagacctatgcccatctgtttgacgataaagtcatgaaacagctcaagcggcggcgctacactgggtggggtagactctccaggaaactcataaacggcatccgcgacaaacagagcggaaagaccatcctggatttcctgaaatccgacggattcgctaacaggaacttcatgcaactgattcacgatgactctctgacatttaaagaggacatccagaaggcacaggtgagcggtcaaggcgacagcctgcacgagcacatcgccaacctcgctggatcacccgccataaagaagggaatactgcagacagtcaaggtcgtggacgaactcgtcaaagtgatgggtcggcacaagccagagaatatcgttatcgaaatggcaagggagaaccaaaccacccagaagggccagaagaactctcgggaacggatgaaaagaatcgaagagggaattaaggagctgggatctcagatactgaaggagcaccctgtggagaatacacagctccagaacgagaaactctacctgtactacctccagaacgggcgggacatgtacgttgaccaggaactcgacatcaaccggctgtccgattatgacgtggaccatattgttccacagtccttcctcaaagatgactccattgacaacaaggtgctgaccagatccgataagaatcgcggtaagtctgacaatgttccatcagaagaggtggtcaagaagatgaagaattactggcggcagctcctcaacgccaaactgatcacccagcggaagtttgacaatctgactaaggcagaaagaggaggtctgagcgaactcgacaaggccggctttattaagaggcaactggtcgaaacacgccagattaccaaacacgtggcacaaatcctcgactctaggatgaacactaagtacgatgagaacgataagctgatcagggaagtgaaagtgataactctgaagagcaagctggtgtctgacttccggaaggactttcaattctacaaagttcgcgaaataaacaattaccatcatgctcacgatgcctatctcaatgctgtcgttggcaccgccctgatcaagaaataccctaaactggagtctgagttcgtgtacggtgactataaagtctacgatgtgaggaagatgatagcaaagtctgagcaagagattggcaaagccaccgccaagtacttcttctactctaatatcatgaatttctttaagactgagataaccctggctaacggcgaaatccggaagcgcccactgatcgaaacaaacggagaaacaggagaaatcgtgtgggataaaggcagggacttcgcaactgtgcggaaggtgctgtccatgccacaagtcaatatcgtgaagaagaccgaagtgcagaccggcggattctcaaaggagagcatcctgccaaagcggaactctgacaagctgatcgccaggaagaaagattgggacccaaagaagtatggcggtttcgattcccctacagtggcttattccgttctggtcgtggcaaaagtggagaaaggcaagtccaagaaactcaagtctgttaaggagctgctcggaattactattatggagagatccagcttcgagaagaatccaatcgatttcctggaagctaagggctataaagaagtgaagaaagatctcatcatcaaactgcccaagtactctctctttgagctggagaatggtaggaagcggatgctggcctccgccggagagctgcagaaaggaaacgagctggctctgccctccaaatacgtgaacttcctgtatctggcctcccactacgagaaactcaaaggtagccctgaagacaatgagcagaagcaactctttgttgagcaacataaacactacctggacgaaatcattgaacagattagcgagttcagcaagcgggttattctggccgatgcaaacctcgataaagtgctgagcgcatataataagcacagggacaagccaattcgcgaacaagcagagaatattatccacctctttactctgactaatctgggcgctcctgctgccttcaagtatttcgatacaactattgacaggaagcggtacacctctaccaaagaagttctcgatgccaccctgatacaccagtcaattaccggactgtacgagactcgcatcgacctgtctcagctcggcggcgacGGTACCcccaagaagaagaggaaagtctcgagcggtggagctgcaggataggggcccttcgaaggtaagcctatccctaaccctctcctcggtctcgattctacgcgtaccggtcatcatcaccatcaccattgagtttAAACCCGCTGATCAGCCTCGACTGTGCCTTCTAGTTGCCAGCCATCTGTTGTTTGCCCCTCCCCCGTGCCTTCCTTGACCCTGGAAGGTGCCACTCCCACTGTCCTTTCCTAATAAAATGAGGAAATTGCATCGCATTGTCTGAGTAGGTGTCATTCTATTCTGGGGGGTGGGGTGGGGCAGGACAGCAAGGGGGAGGATTGGGAAGACAATAGCAGGCATGCTGGGGATGCGGTGGGCTCTATGGCTTCTGAGGCGGAAAGAACCAGCTGGGGCTCTAGGGGGTATCCCCACGCGCCCTGTAGCGGCGCATTAAGCGCGGCGGGTGTGGTGGTTACGCGCAGCGTGACCGCTACACTTGCCAGCGCCCTAGCGCCCGCTCCTTTCGCTTTCTTCCCTTCCTTTCTCGCCACGTTCGCCGGCTTTCCCCGTCAAGCTCTAAATCGGGGCATCCCTTTAGGGTTCCGATTTAGTGCTTTACGGCACCTCGACCCCAAAAAACTTGATTAGGGTGATGGTTCACGTAGTGGGCCATCGCCCTGATAGACGGTTTTTCGCCCTTTGACGTTGGAGTCCACGTTCTTTAATAGTGGACTCTTGTTCCAAACTGGAACAACACTCAACCCTATCTCGGTCTATTCTTTTGATTTATAAGGGATTTTGGGGATTTCGGCCTATTGGTTAAAAAATGAGCTGATTTAACAAAAATTTAACGCGAATTAATTCTGTGGAATGTGTGTCAGTTAGGGTGTGGAAAGTCCCCAGGCTCCCCAGGCAGGCAGAAGTATGCAAAGCATGCATCTCAATTAGTCAGCAACCAGGTGTGGAAAGTCCCCAGGCTCCCCAGCAGGCAGAAGTATGCAAAGCATGCATCTCAATTAGTCAGCAACCATAGTCCCGCCCCTAACTCCGCCCATCCCGCCCCTAACTCCGCCCAGTTCCGCCCATTCTCCGCCCCATGGCTGACTAATTTTTTTTATTTATGCAGAGGCCGAGGCCGCCTCTGCCTCTGAGCTATTCCAGAAGTAGTGAGGAGGCTTTTTTGGAGGCCTAGGCTTTTGCAAAAAGCTCCCGGGAGCTTGTATATCCATTTTCGGATCTGATCAGCACGTGTTGACAATTAATCATCGGCATAGTATATCGGCATAGTATAATACGACAAGGTGAGGAACTAAACCATGGCCAAGCCTTTGTCTCAAGAAGAATCCACCCTCATTGAAAGAGCAACGGCTACAATCAACAGCATCCCCATCTCTGAAGACTACAGCGTCGCCAGCGCAGCTCTCTCTAGCGACGGCCGCATCTTCACTGGTGTCAATGTATATCATTTTACTGGGGGACCTTGTGCAGAACTCGTGGTGCTGGGCACTGCTGCTGCTGCGGCAGCTGGCAACCTGACTTGTATCGTCGCGATCGGAAATGAGAACAGGGGCATCTTGAGCCCCTGCGGACGGTGTCGACAGGTGCTTCTCGATCTGCATCCTGGGATCAAAGCGATAGTGAAGGACAGTGATGGACAGCCGACGGCAGTTGGGATTCGTGAATTGCTGCCCTCTGGTTATGTGTGGGAGGGCTAAGCACTTCGTGGCCGAGGAGCAGGACTGACACGTGCTACGAGATTTCGATTCCACCGCCGCCTTCTATGAAAGGTTGGGCTTCGGAATCGTTTTCCGGGACGCCGGCTGGATGATCCTCCAGCGCGGGGATCTCATGCTGGAGTTCTTCGCCCACCCCAACTTGTTTATTGCAGCTTATAATGGTTACAAATAAAGCAATAGCATCACAAATTTCACAAATAAAGCATTTTTTTCACTGCATTCTAGTTGTGGTTTGTCCAAACTCATCAATGTATCTTATCATGTCTGTATACCGTCGACCTCTAGCTAGAGCTTGGCGTAATCATGGTCATAGCTGTTTCCTGTGTGAAATTGTTATCCGCTCACAATTCCACACAACATACGAGCCGGAAGCATAAAGTGTAAAGCCTGGGGTGCCTAATGAGTGAGCTAACTCACATTAATTGCGTTGCGCTCACTGCCCGCTTTCCAGTCGGGAAACCTGTCGTGCCAGCTGCATTAATGAATCGGCCAACGCGCGGGGAGAGGCGGTTTGCGTATTGGGCGCTCTTCCGCTTCCTCGCTCACTGACTCGCTGCGCTCGGTCGTTCGGCTGCGGCGAGCGGTATCAGCTCACTCAAAGGCGGTAATACGGTTATCCACAGAATCAGGGGATAACGCAGGAAAGAACATGTGAGCAAAAGGCCAGCAAAAGGCCAGGAACCGTAAAAAGGCCGCGTTGCTGGCGTTTTTCCATAGGCTCCGCCCCCCTGACGAGCATCACAAAAATCGACGCTCAAGTCAGAGGTGGCGAAACCCGACAGGACTATAAAGATACCAGGCGTTTCCCCCTGGAAGCTCCCTCGTGCGCTCTCCTGTTCCGACCCTGCCGCTTACCGGATACCTGTCCGCCTTTCTCCCTTCGGGAAGCGTGGCGCTTTCTCAATGCTCACGCTGTAGGTATCTCAGTTCGGTGTAGGTCGTTCGCTCCAAGCTGGGCTGTGTGCACGAACCCCCCGTTCAGCCCGACCGCTGCGCCTTATCCGGTAACTATCGTCTTGAGTCCAACCCGGTAAGACACGACTTATCGCCACTGGCAGCAGCCACTGGTAACAGGATTAGCAGAGCGAGGTATGTAGGCGGTGCTACAGAGTTCTTGAAGTGGTGGCCTAACTACGGCTACACTAGAAGGACAGTATTTGGTATCTGCGCTCTGCTGAAGCCAGTTACCTTCGGAAAAAGAGTTGGTAGCTCTTGATCCGGCAAACAAACCACCGCTGGTAGCGGTGGTTTTTTTGTTTGCAAGCAGCAGATTACGCGCAGAAAAAAAGGATCTCAAGAAGATCCTTTGATCTTTTCTACGGGGTCTGACGCTCAGTGGAACGAAAACTCACGTTAAGGGATTTTGGTCATGAGATTATCAAAAAGGATCTTCACCTAGATCCTTTTAAATTAAAAATGAAGTTTTAAATCAATCTAAAGTATATATGAGTAAACTTGGTCTGACAGTTACCAATGCTTAATCAGTGAGGCACCTATCTCAGCGATCTGTCTATTTCGTTCATCCATAGTTGCCTGACTCCCCGTCGTGTAGATAACTACGATACGGGAGGGCTTACCATCTGGCCCCAGTGCTGCAATGATACCGCGAGACCCACGCTCACCGGCTCCAGATTTATCAGCAATAAACCAGCCAGCCGGAAGGGCCGAGCGCAGAAGTGGTCCTGCAACTTTATCCGCCTCCATCCAGTCTATTAATTGTTGCCGGGAAGCTAGAGTAAGTAGTTCGCCAGTTAATAGTTTGCGCAACGTTGTTGCCATTGCTACAGGCATCGTGGTGTCACGCTCGTCGTTTGGTATGGCTTCATTCAGCTCCGGTTCCCAACGATCAAGGCGAGTTACATGATCCCCCATGTTGTGCAAAAAAGCGGTTAGCTCCTTCGGTCCTCCGATCGTTGTCAGAAGTAAGTTGGCCGCAGTGTTATCACTCATGGTTATGGCAGCACTGCATAATTCTCTTACTGTCATGCCATCCGTAAGATGCTTTTCTGTGACTGGTGAGTACTCAACCAAGTCATTCTGAGAATAGTGTATGCGGCGACCGAGTTGCTCTTGCCCGGCGTCAATACGGGATAATACCGCGCCACATAGCAGAACTTTAAAAGTGCTCATCATTGGAAAACGTTCTTCGGGGCGAAAACTCTCAAGGATCTTACCGCTGTTGAGATCCAGTTCGATGTAACCCACTCGTGCACCCAACTGATCTTCAGCATCTTTTACTTTCACCAGCGTTTCTGGGTGAGCAAAAACAGGAAGGCAAAATGCCGCAAAAAAGGGAATAAGGGCGACACGGAAATGTTGAATACTCATACTCTTCCTTTTTCAATATTATTGAAGCATTTATCAGGGTTATTGTCTCATGAGCGGATACATATTTGAATGTATTTAGAAAAATAAACAAATAGGGGTTCCGCGCACATTTCCCCGAAAAGTGCCACCTGACGTC

CMV-pembrolizumab-HC-CMV-pembrolizumab-LC-SV40-GS plasmid: ATATAGCATAAATCAATATTGGCTATTGGCCATTGCATACGTTGTATCCATATCATAATATGTACATTTATATTGGCTCATGTCCAACATTACCGCCATGTTGACATTGATTATTGACTAGTTATTAATAGTAATCAATTACGGGGTCATTAGTTCATAGCCCATATATGGAGTTCCGCGTTACATAACTTACGGTAAATGGCCCGCCTGGCTGACCGCCCAACGACCCCCGCCCATTGACGTCAATAATGACGTATGTTCCCATAGTAACGCCAATAGGGACTTTCCATTGACGTCAATGGGTGGAGTATTTACGGTAAACTGCCCACTTGGCAGTACATCAAGTGTATCATATGCCAAGTACGCCCCCTATTGACGTCAATGACGGTAAATGGCCCGCCTGGCATTATGCCCAGTACATGACCTTATGGGACTTTCCTACTTGGCAGTACATCTACGTATTAGTCATCGCTATTACCATGGTGATGCGGTTTTGGCAGTACATCAATGGGCGTGGATAGCGGTTTGACTCACGGGGATTTCCAAGTCTCCACCCCATTGACGTCAATGGGAGTTTGTTTTGGCACCAAAATCAACGGGACTTTCCAAAATGTCGTAACAACTCCGCCCCATTGACGCAAATGGGCGGTAGGCGTGTACGGTGGGAGGTCTATATAAGCAGAGCTCGTTTAGTGAACCGTCAGATCGCCTGGAGACGCCATCCACGCTGTTTTGACCTCCATAGAAGACACCGGGACCGATCCAGCCTCCGCGGCCGGGAACGGTGCATTGGAACGCGGATTCCCCGTGCCAAGAGTGACGTAAGTACCGCCTATAGAGTCTATAGGCCCACCCCCTTGGCTTCTTATGCATGCTATACTGTTTTTGGCTTGGGGTCTATACACCCCCGCTTCCTCATGTTATAGGTGATGGTATAGCTTAGCCTATAGGTGTGGGTTATTGACCATTATTGACCACTCCCCTATTGGTGACGATACTTTCCATTACTAATCCATAACATGGCTCTTTGCCACAACTCTCTTTATTGGCTATATGCCAATACACTGTCCTTCAGAGACTGACACGGACTCTGTATTTTTACAGGATGGGGTCTCATTTATTATTTACAAATTCACATATACAACACCACCGTCCCCAGTGCCCGCAGTTTTTATTAAACATAACGTGGGATCTCCACGCGAATCTCGGGTACGTGTTCCGGACATGGCCACTTCTCCGGTAGCGGCGGAGCTTCTACATCCGAGCCCTGCTCCCATGCCTCCAGCGACTCATGGTCGCTCGGCAGCTCCTTGCTCCTAACAGTGGAGGCCAGACTTAGGCACAGCACGATGCCCACCACCACCAGTGTGCCGCACAAGGCCGTGGCGGTAGGGTATGTGTCTGAAAATGAGCTCGGGGAGCGGGCTTGCACCGCTGACGCATTTGGAAGACTTAAGGCAGCGGCAGAAGAAGATGCAGGCAGCTGAGTTGTTGTGTTCTGATAAGAGTCAGAGGTAACTCCCGTTGCGGTGCTGTTAACGGTGGAGGGCAGTGTAGTCTGAGCAGTACTCGTTGCTGCCGCGCGCGCCACCAGACATAATAGCTGACAGACTAACAGACTGTTCCTTTCCATGGGTCTTTTCTGCAGTCACCGTCCTTGACACGAAGCTTTAGACCACCATGGACATGAGGGTGCCCGCTCAGCTCCTGGGGCTCCTGCTGCTGTGGCTGAGAGGTGCCAGATGTGAGATCGTGCTGACACAGAGCCCCGCTACTTTATCTTTATCCCCCGGTGAGAGGGCCACCTTATCTTGTAGGGCCTCCAAGGGAGTGTCCACCTCCGGCTACAGCTATTTACACTGGTACCAGCAGAAGCCCGGTCAAGCTCCTCGGCTGCTGATCTATTTAGCCTCCTATTTAGAGTCCGGCGTGCCCGCTAGGTTCAGCGGCAGCGGCTCCGGAACCGACTTCACTTTAACCATCTCCTCTTTAGAGCCCGAGGACTTCGCCGTGTACTACTGCCAGCACTCCCGGGATCTGCCTCTGACATTCGGCGGCGGCACAAAGGTGGAAATCAAGAGGACCGTGGCCGCCCCCAGCGTGTTCATCTTCCCTCCCTCCGACGAGCAGCTGAAATCCGGCACCGCCTCCGTGGTGTGTTTACTCAACAACTTCTACCCTCGTGAGGCCAAGGTGCAGTGGAAGGTGGACAACGCTTTACAGTCCGGCAACTCCCAAGAATCCGTGACCGAGCAAGATTCCAAGGACTCCACCTACTCTTTATCCAGCACTTTAACTTTATCCAAGGCTGACTACGAGAAGCACAAGGTGTACGCTTGTGAGGTGACACACCAAGGTTTATCCTCCCCCGTGACCAAGTCCTTCAATCGTGGCGAGTGCTGAGAATTCATTGATCATAATCAGCCATACCACATTTGTAGAGGTTTTACTTGCTTTAAAAAACCTCCCACACCTCCCCCTGAACCTGAAACATAAAATGAATGCAATTGTTGTTGTTAACTTGTTTATTGCAGCTTATAATGGTTACAAATAAAGCAATAGCATCACAAATTTCACAAATAAAGCATTTTTTTCACTGCATTCTAGTTGTGGTTTGTCCAAACTCATCAATGTATCTTATCATGTCTGGCGGCCGCCGATATTTGAAAATATGGCATATTGAAAATGTCGCCGATGTGAGTTTCTGTGTAACTGATATCGCCATTTTTCCAAAAGTGATTTTTGGGCATACGCGATATCTGGCGATAGCGCTTATATCGTTTACGGGGGATGGCGATAGACGACTTTGGTGACTTGGGCGATTCTGTGTGTCGCAAATATCGCAGTTTCGATATAGGTGACAGACGATATGAGGCTATATCGCCGATAGAGGCGACATCAAGCTGGCACATGGCCAATGCATATCGATCTATACATTGAATCAATATTGGCCATTAGCCATATTATTCATTGGTTATATAGCATAAATCAATATTGGCTATTGGCCATTGCATACGTTGTATCCATATCATAATATGTACATTTATATTGGCTCATGTCCAACATTACCGCCATGTTGACATTGATTATTGACTAGTTATTAATAGTAATCAATTACGGGGTCATTAGTTCATAGCCCATATATGGAGTTCCGCGTTACATAACTTACGGTAAATGGCCCGCCTGGCTGACCGCCCAACGACCCCCGCCCATTGACGTCAATAATGACGTATGTTCCCATAGTAACGCCAATAGGGACTTTCCATTGACGTCAATGGGTGGAGTATTTACGGTAAACTGCCCACTTGGCAGTACATCAAGTGTATCATATGCCAAGTACGCCCCCTATTGACGTCAATGACGGTAAATGGCCCGCCTGGCATTATGCCCAGTACATGACCTTATGGGACTTTCCTACTTGGCAGTACATCTACGTATTAGTCATCGCTATTACCATGGTGATGCGGTTTTGGCAGTACATCAATGGGCGTGGATAGCGGTTTGACTCACGGGGATTTCCAAGTCTCCACCCCATTGACGTCAATGGGAGTTTGTTTTGGCACCAAAATCAACGGGACTTTCCAAAATGTCGTAACAACTCCGCCCCATTGACGCAAATGGGCGGTAGGCGTGTACGGTGGGAGGTCTATATAAGCAGAGCTCGTTTAGTGAACCGTCAGATCGCCTGGAGACGCCATCCACGCTGTTTTGACCTCCATAGAAGACACCGGGACCGATCCAGCCTCCGCGGCCGGGAACGGTGCATTGGAACGCGGATTCCCCGTGCCAAGAGTGACGTAAGTACCGCCTATAGAGTCTATAGGCCCACCCCCTTGGCTTCTTATGCATGCTATACTGTTTTTGGCTTGGGGTCTATACACCCCCGCTTCCTCATGTTATAGGTGATGGTATAGCTTAGCCTATAGGTGTGGGTTATTGACCATTATTGACCACTCCCCTATTGGTGACGATACTTTCCATTACTAATCCATAACATGGCTCTTTGCCACAACTCTCTTTATTGGCTATATGCCAATACACTGTCCTTCAGAGACTGACACGGACTCTGTATTTTTACAGGATGGGGTCTCATTTATTATTTACAAATTCACATATACAACACCACCGTCCCCAGTGCCCGCAGTTTTTATTAAACATAACGTGGGATCTCCACGCGAATCTCGGGTACGTGTTCCGGACATGGCCACTTCTCCGGTAGCGGCGGAGCTTCTACATCCGAGCCCTGCTCCCATGCCTCCAGCGACTCATGGTCGCTCGGCAGCTCCTTGCTCCTAACAGTGGAGGCCAGACTTAGGCACAGCACGATGCCCACCACCACCAGTGTGCCGCACAAGGCCGTGGCGGTAGGGTATGTGTCTGAAAATGAGCTCGGGGAGCGGGCTTGCACCGCTGACGCATTTGGAAGACTTAAGGCAGCGGCAGAAGAAGATGCAGGCAGCTGAGTTGTTGTGTTCTGATAAGAGTCAGAGGTAACTCCCGTTGCGGTGCTGTTAACGGTGGAGGGCAGTGTAGTCTGAGCAGTACTCGTTGCTGCCGCGCGCGCCACCAGACATAATAGCTGACAGACTAACAGACTGTTCCTTTCCATGGGTCTTTTCTGCAGTCACCGTCCTTGACACGAAGCTTTAGACCACCATGGACATGAGGGTGCCCGCTCAGCTCCTGGGGCTCCTGCTGCTGTGGCTGAGAGGTGCCAGATGTCAAGTTCAGCTGGTGCAGAGCGGCGTGGAGGTGAAGAAACCCGGTGCTTCCGTGAAGGTGTCTTGTAAGGCTTCCGGCTACACCTTCACCAATTACTATATGTATTGGGTGAGGCAAGCTCCCGGTCAAGGCCTCGAGTGGATGGGAGGCATTAACCCTAGCAACGGCGGCACCAACTTCAACGAAAAATTCAAGAATCGGGTGACTTTAACCACCGACTCCAGCACAACCACCGCTTACATGGAGCTGAAGTCCCTCCAGTTCGACGACACAGCCGTGTACTACTGTGCTCGTAGGGACTACCGGTTCGACATGGGCTTCGACTACTGGGGCCAAGGTACAACAGTGACAGTGAGCTCCGCCTCCACAAAGGGCCCCAGCGTGTTCCCTCTGGCTCCTTGCTCTCGTAGCACCAGCGAGAGCACAGCTGCTTTAGGCTGTCTGGTCAAGGATTACTTTCCCGAGCCCGTTACCGTGAGCTGGAACTCCGGCGCTCTGACCTCCGGCGTGCACACATTTCCCGCCGTTTTACAGAGCAGCGGACTCTACTCTTTATCCTCCGTGGTGACAGTGCCCAGCAGCTCTTTAGGCACCAAGACCTACACTTGTAACGTCGACCACAAGCCTAGCAATACCAAGGTGGACAAGAGGGTGGAAAGCAAGTACGGCCCCCCTTGTCCTCCTTGCCCCGCTCCCGAATTTTTAGGAGGCCCTTCCGTGTTCCTCTTCCCTCCCAAGCCCAAGGACACACTGATGATCTCTCGTACCCCCGAAGTGACATGTGTGGTGGTGGACGTCAGCCAAGAAGACCCCGAGGTGCAGTTCAATTGGTACGTGGACGGCGTCGAGGTCCATAACGCCAAGACCAAGCCTCGTGAGGAACAATTCAACAGCACCTATCGGGTGGTCAGCGTGCTGACCGTCCTCCACCAAGATTGGCTGAACGGCAAAGAGTATAAATGCAAGGTGTCCAACAAGGGTTTACCTAGCTCCATTGAGAAGACCATCTCCAAGGCCAAGGGACAGCCTCGGGAGCCCCAAGTTTACACACTGCCTCCCTCCCAAGAAGAGATGACCAAGAACCAAGTTTCCCTCACTTGTTTAGTCAAGGGCTTTTACCCTAGCGACATCGCTGTGGAGTGGGAGTCCAATGGCCAGCCCGAAAACAACTACAAGACCACACCTCCCGTGCTGGACAGCGACGGCTCCTTCTTTCTGTACTCCCGGCTGACCGTGGACAAGTCTCGTTGGCAAGAAGGCAACGTGTTCTCTTGTTCCGTGATGCATGAGGCTCTGCACAACCACTACACACAGAAGTCTTTATCTTTATCTTTAGGAAAGTGAGAATTCATTGATCATAATCAGCCATACCACATTTGTAGAGGTTTTACTTGCTTTAAAAAACCTCCCACACCTCCCCCTGAACCTGAAACATAAAATGAATGCAATTGTTGTTGTTAACTTGTTTATTGCAGCTTATAATGGTTACAAATAAAGCAATAGCATCACAAATTTCACAAATAAAGCATTTTTTTCACTGCATTCTAGTTGTGGTTTGTCCAAACTCATCAATGTATCTTATCATGTCTGGATCCTCTACGCCGGACGCATCGTGGCCGGCATCACCGGCGCCACAGGTGCGGTTGCTGGCGCCTATATCGCCGACATCACCGATGGGGAAGATCGGGCTCGCCACTTCGGGCTCATGAGCGCTTGTTTCGGCGTGGGTATGGTGGCAGGCCCCGTGGCCGGGGGACTGTTGGGCGCCATCTCCTTGCATGCACCATTCCTTGCGGCGGCGGTGCTCAACGGCCTCAACCTACTACTGGGCTGCTTCCTAATGCAGGAGTCGCATAAGGGAGAGCGTCGACCTCGGGCCGCGTTGCTGGCGTTTTTCCATAGGCTCCGCCCCCCTGACGAGCATCACAAAAATCGACGCTCAAGTCAGAGGTGGCGAAACCCGACAGGACTATAAAGATACCAGGCGTTTCCCCCTGGAAGCTCCCTCGTGCGCTCTCCTGTTCCGACCCTGCCGCTTACCGGATACCTGTCCGCCTTTCTCCCTTCGGGAAGCGTGGCGCTTTCTCATAGCTCACGCTGTAGGTATCTCAGTTCGGTGTAGGTCGTTCGCTCCAAGCTGGGCTGTGTGCACGAACCCCCCGTTCAGCCCGACCGCTGCGCCTTATCCGGTAACTATCGTCTTGAGTCCAACCCGGTAAGACACGACTTATCGCCACTGGCAGCAGCCACTGGTAACAGGATTAGCAGAGCGAGGTATGTAGGCGGTGCTACAGAGTTCTTGAAGTGGTGGCCTAACTACGGCTACACTAGAAGAACAGTATTTGGTATCTGCGCTCTGCTGAAGCCAGTTACCTTCGGAAAAAGAGTTGGTAGCTCTTGATCCGGCAAACAAACCACCGCTGGTAGCGGTGGTTTTTTTGTTTGCAAGCAGCAGATTACGCGCAGAAAAAAAGGATCTCAAGAAGATCCTTTGATCTTTTCTACGGGGTCTGACGCTCAGTGGAACGAAAACTCACGTTAAGGGATTTTGGTCATGAGATTATCAAAAAGGATCTTCACCTAGATCCTTTTAAATTAAAAATGAAGTTTTAAATCAATCTAAAGTATATATGAGTAAACTTGGTCTGACAGTTACCAATGCTTAATCAGTGAGGCACCTATCTCAGCGATCTGTCTATTTCGTTCATCCATAGTTGCCTGACTCCCCGTCGTGTAGATAACTACGATACGGGAGGGCTTACCATCTGGCCCCAGTGCTGCAATGATACCGCGAGACCCACGCTCACCGGCTCCAGATTTATCAGCAATAAACCAGCCAGCCGGAAGGGCCGAGCGCAGAAGTGGTCCTGCAACTTTATCCGCCTCCATCCAGTCTATTAATTGTTGCCGGGAAGCTAGAGTAAGTAGTTCGCCAGTTAATAGTTTGCGCAACGTTGTTGCCATTGCTACAGGCATCGTGGTGTCACGCTCGTCGTTTGGTATGGCTTCATTCAGCTCCGGTTCCCAACGATCAAGGCGAGTTACATGATCCCCCATGTTGTGCAAAAAAGCGGTTAGCTCCTTCGGTCCTCCGATCGTTGTCAGAAGTAAGTTGGCCGCAGTGTTATCACTCATGGTTATGGCAGCACTGCATAATTCTCTTACTGTCATGCCATCCGTAAGATGCTTTTCTGTGACTGGTGAGTACTCAACCAAGTCATTCTGAGAATAGTGTATGCGGCGACCGAGTTGCTCTTGCCCGGCGTCAATACGGGATAATACCGCGCCACATAGCAGAACTTTAAAAGTGCTCATCATTGGAAAACGTTCTTCGGGGCGAAAACTCTCAAGGATCTTACCGCTGTTGAGATCCAGTTCGATGTAACCCACTCGTGCACCCAACTGATCTTCAGCATCTTTTACTTTCACCAGCGTTTCTGGGTGAGCAAAAACAGGAAGGCAAAATGCCGCAAAAAAGGGAATAAGGGCGACACGGAAATGTTGAATACTCATACTCTTCCTTTTTCAATATTATTGAAGCATTTATCAGGGTTATTGTCTCATGAGCGGATACATATTTGAATGTATTTAGAAAAATAAACAAATAGGGGTTCCGCGCACATTTCCCCGAAAAGTGCCACCTGACGTCTAAGAAACCATTATTATCATGACATTAACCTATAAAAATAGGCGTATCACGAGGCCCTGATGGCTCTTTGCGGCACCCATCGTTCGTAATGTTCCGTGGCACCGAGGACAACCCTCAAGAGAAAATGTAATCACACTGGCTCACCTTCGGGTGGGCCTTTCTGCGTTTATAAGGAGACACTTTATGTTTAAGAAGGTTGGTAAATTCCTTGCGGCTTTGGCAGCCAAGCTAGATCCGGCTGTGGAATGTGTGTCAGTTAGGGTGTGGAAAGTCCCCAGGCTCCCCAGCAGGCAGAAGTATGCAAAGCATGCATCTCAATTAGTCAGCAACCAGGTGTGGAAAGTCCCCAGGCTCCCCAGCAGGCAGAAGTATGCAAAGCATGCATCTCAATTAGTCAGCAACCATAGTCCCGCCCCTAACTCCGCCCATCCCGCCCCTAACTCCGCCCAGTTCCGCCCATTCTCCGCCCCATGGCTGACTAATTTTTTTTATTTATGCAGAGGCCGAGGCCGCCTCGGCCTCTGAGCTATTCCAGAAGTAGTGAGGAGGCTTTTTTGGAGGCCTAGGCTTTTGCAAAAAGCTAGCTTGGGGCCACCGCTCAGAGCACCTTCCACCATGGCCACCTCAGCAAGTTCCCACTTGAACAAAAACATCAAGCAAATGTACTTGTGCCTGCCCCAGGGTGAGAAAGTCCAAGCCATGTATATCTGGGTTGATGGTACTGGAGAAGGACTGCGCTGCAAAACCCGCACCCTGGACTGTGAGCCCAAGTGTGTAGAAGAGTTACCTGAGTGGAATTTTGATGGCTCTAGTACCTTTCAGTCTGAGGGCTCCAACAGTGACATGTATCTCAGCCCTGTTGCCATGTTTCGGGACCCCTTCCGCAGAGATCCCAACAAGCTGGTGTTCTGTGAAGTTTTCAAGTACAACCGGAAGCCTGCAGAGACCAATTTAAGGCACTCGTGTAAACGGATAATGGACATGGTGAGCAACCAGCACCCCTGGTTTGGAATGGAACAGGAGTATACTCTGATGGGAACAGATGGGCACCCTTTTGGTTGGCCTTCCAATGGCTTTCCTGGGCCCCAAGGTCCGTATTACTGTGGTGTGGGCGCAGACAAAGCCTATGGCAGGGATATCGTGGAGGCTCACTACCGCGCCTGCTTGTATGCTGGGGTCAAGATTACAGGAACAAATGCTGAGGTCATGCCTGCCCAGTGGGAGTTCCAAATAGGACCCTGTGAAGGAATCCGCATGGGAGATCATCTCTGGGTGGCCCGTTTCATCTTGCATCGAGTATGTGAAGACTTTGGGGTAATAGCAACCTTTGACCCCAAGCCCATTCCTGGGAACTGGAATGGTGCAGGCTGCCATACCAACTTTAGCACCAAGGCCATGCGGGAGGAGAATGGTCTGAAGCACATCGAGGAGGCCATCGAGAAACTAAGCAAGCGGCACCGGTACCACATTCGAGCCTACGATCCCAAGGGGGGCCTGGACAATGCCCGTCGTCTGACTGGGTTCCACGAAACGTCCAACATCAACGACTTTTCTGCTGGTGTCGCCAATCGCAGTGCCAGCATCCGCATTCCCCGGACTGTCGGCCAGGAGAAGAAAGGTTACTTTGAAGACCGCCGCCCCTCTGCCAATTGTGACCCCTTTGCAGTGACAGAAGCCATCGTCCGCACATGCCTTCTCAATGAGACTGGCGACGAGCCCTTCCAATACAAAAACTAATTAGACTTTGAGTGATCTTGAGCCTTTCCTAGTTCATCCCACCCCGCCCCAGAGAGATCTTTGTGAAGGAACCTTACTTCTGTGGTGTGACATAATTGGACAAACTACCTACAGAGATTTAAAGCTCTAAGGTAAATATAAAATTTTTAAGTGTATAATGTGTTAAACTACTGATTCTAATTGTTTGTGTATTTTAGATTCCAACCTATGGAACTGATGAATGGGAGCAGTGGTGGAATGCCTTTAATGAGGAAAACCTGTTTTGCTCAGAAGAAATGCCATCTAGTGATGATGAGGCTACTGCTGACTCTCAACATTCTACTCCTCCAAAAAAGAAGAGAAAGGTAGAAGACCCCAAGGACTTTCCTTCAGAATTGCTAAGTTTTTTGAGTCATGCTGTGTTTAGTAATAGAACTCTTGCTTGCTTTGCTATTTACACCACAAAGGAAAAAGCTGCACTGCTATACAAGAAAATTATGGAAAAATATTCTGTAACCTTTATAAGTAGGCATAACAGTTATAATCATAACATACTGTTTTTTCTTACTCCACACAGGCATAGAGTGTCTGCTATTAATAACTATGCTCAAAAATTGTGTACCTTTAGCTTTTTAATTTGTAAAGGGGTTAATAAGGAATATTTGATGTATAGTGCCTTGACTAGAGATCATAATCAGCCATACCACATTTGTAGAGGTTTTACTTGCTTTAAAAAACCTCCCACACCTCCCCCTGAACCTGAAACATAAAATGAATGCAATTGTTGTTGTTAACTTGTTTATTGCAGCTTATAATGGTTACAAATAAAGCAATAGCATCACAAATTTCACAAATAAAGCATTTTTTTCACTGCATTCTAGTTGTGGTTTGTCCAAACTCATCAATGTATCTTATCATGTCTGGATCTAGCTTCGTGTCAAGGACGGTGACTGCAGTGAATAATAAAATGTGTGTTTGTCCGAAATACGCGTTTTGAGATTTCTGTCGCCGACTAAATTCATGTCGCGCGATAGTGGTGTTTATCGCCGATAGAGATGGCGATATTGGAAAAATCGATATTTGAAAATATGGCATATTGAAAATGTCGCCGATGTGAGTTTCTGTGTAACTGATATCGCCATTTTTCCAAAAGTGATTTTTGGGCATACGCGATATCTGGCGATAGCGCTTATATCGTTTACGGGGGATGGCGATAGACGACTTTGGTGACTTGGGCGATTCTGTGTGTCGCAAATATCGCAGTTTCGATATAGGTGACAGACGATATGAGGCTATATCGCCGATAGAGGCGACATCAAGCTGGCACATGGCCAATGCATATCGATCTATACATTGAATCAATATTGGCCATTAGCCATATTATTCATTGGTT
