# Supplementary material for: Base editing-mediated exon-specific knockout of glutamine synthetase generates a high-productivity CHO cell line for antibody manufacturing
Source: Bioresour Bioprocess. 2026 Oct 23;13(1):156. doi: 10.1186/s40643-026-01150-0 (PMC13694664; doi:10.1186/s40643-026-01150-0)
Supplement: Supplementary file 3 [file 40643_2026_1150_MOESM1_ESM.docx]

# **Supplementary figure legends Supplementary Figure 1. Validation of GS-knockout phenotypes and GS protein expression in commercial CHO cell lines.**

# (a, b) Cell viability profiles of untransfected ZN (a) and AK1 (b) cells cultured in medium containing 6 mM glutamine without MSX, glutamine-free medium without MSX, or glutamine-free medium supplemented with 10 μM MSX. (c) Viable cell density and viability of ZN and AK1 cells cultured in glutamine-free medium. Data in (a-c) were obtained from a single independent 30-mL culture for each cell line/condition using a standardized industrial CDMO platform. Analytical measurements were performed in technical triplicate, but these were not treated as biological replicates. (d) Western blot analysis of GS protein in EK1, ZN, and AK1 cells cultured with 6 mM glutamine; GAPDH served as the loading control.

# **Supplementary Figure 2. Design of genome-editing strategies, sequence validation of exon-specific GS-edited clones, and selection of an UPF1-targeting shRNA.**

# (a) Distribution of target sites in Glul exon 6 for seven TALEN pairs, 10 CRISPR/Cas9 sgRNAs, one validated ZFN pair, and eight CBE sgRNAs. Target codons converted to PTCs by CBE are indicated. (b) Sanger sequencing confirmation of independent GS-edited clones carrying PTC-generating substitutions in exons 2-7. The edited bases and resulting amino acid changes (Q27X, W60X, Q135X, Q201X, Q205X, and R286X) are indicated. (c) RT-qPCR comparison of Upf1 knockdown by four candidate shRNAs, with a non-targeting scramble shRNA and wild-type cells as controls. Data are mean ± SD from three independent biological replicates (n = 3). (d) Western blot validation of UPF1 knockdown by the four candidate shRNAs; GAPDH served as the loading control. shRNA4 showed the greatest knockdown and was selected for subsequent experiments.

# **Supplementary Figure 3. Serial-passage growth and viability of E3-E6 cells under different glutamine concentrations.**

# (a-d) Viable cell density profiles of E3 (a), E4 (b), E5 (c), and E6 (d) cells cultured in medium containing 0, 1, 2, or 6 mM glutamine. Cells were inoculated at 5 × 10⁵ viable cells/mL and re-inoculated at the same density every 3 days for five consecutive passages. (e-h) Cell viability profiles corresponding to (a-d). Data were obtained from a single independent 30-mL culture for each cell line condition using a standardized industrial CDMO platform. Analytical measurements were performed in technical triplicate, but these were not treated as biological replicates.

**Supplementary Figure 4. Assessment of GS1 cross-editing and validation of independent E2 and E7 clones.**

(a) Alignment of the exon 7 sgRNA with the GS5 on-target site and the homologous GS1 site, followed by Sanger sequencing of GS1 in three E7 clones. GS1 was homozygously edited in E7-1, heterozygously edited in E7-2, and unedited in E7-3. (b) GS1 transcript levels in E2-E7, ZN, AK1, and EK1 cells measured by GS1-specific RT-qPCR. Data are mean ± SD from three independent biological replicates (n = 3). Statistical significance was assessed by one-way ANOVA followed by Dunnett's multiple-comparisons test using EK1 as the control; ns, not significant; **P* < 0.05. (c, d) Sanger sequencing confirmation of independently derived E2 (Q27X) (c) and E7 (R286X) (d) clones. (e) Western blot analysis of GS protein in three independent E2 clones and three independent E7 clones; EK1 was included as a positive control and GAPDH served as the loading control. (f) Viable cell density and cell viability profiles of three independently derived E7 clones cultured in medium containing 0 or 6 mM glutamine. All three clones exhibited comparable glutamine-dependent growth and viability phenotypes.

**Supplementary Figure 5. Short-term growth and viability of parental and GS-edited cell lines under different glutamine concentrations.**

(a-h) Viable cell density profiles of E2-E7, EK1, and ZN cells cultured in medium containing 0, 1, 2, or 6 mM glutamine. Cells were inoculated at 5 × 10⁵ viable cells/mL and monitored for up to 9 days. (i-p) Cell viability profiles corresponding to (a-h). Data were obtained from a single independent 30-mL culture for each cell line condition using a standardized industrial CDMO platform. Analytical measurements were performed in technical triplicate, but these were not treated as biological replicates.

**Supplementary Figure 6. GS protein domain mapping and targeted transcriptional comparison of E4 and E7 cells.**

(a) Schematic of the Chinese hamster GS protein showing the relative regions encoded by exons 2-7, the annotated N-terminal region and catalytic domain, and the PTC positions in E4 (Q135X) and E7 (R286X). (b-f) RT-qPCR analysis of genes associated with glutamine/nitrogen metabolism (*Gls*, *Glud1*, *Got1*, *Got2*, *Slc1a5*, *and Slc38a2*) (b), apoptosis (*Bcl2*, *Bax*, *Mcl1*, and *Casp3*) (c), proliferation (*Myc*, *Ccnd1*, and *Mki67*) (d), amino-acid stress responses (*Atf4*, *Asns*, and *Sesn2*) (e), and ER stress/unfolded protein response (*Hspa5* and *Xbp1s*) (f) in E4 and E7 cells. Relative expression was calculated by the 2⁻^ΔΔCt^ method with E4 as the calibrator. Data are mean ± SEM from three independent biological replicates (n = 3); dots indicate individual replicates. Statistical comparisons were performed on ΔCt values using multiple unpaired Welch's t-tests followed by the two-stage step-up false-discovery-rate procedure of Benjamini, Krieger, and Yekutieli (Q = 0.05). ns, not significant; *q < 0.05 and **q < 0.01.

**Supplementary Figure 7. Assessment of off-target editing in E7 cells and residual plasmid sequences in GS-edited clones.**

(a) Targeted analysis of the GS5 on-target locus and 13 computationally predicted off-target sites in E7-1. Candidate sites are ordered by CFD score; bubble size represents the CFD score, and bubble color indicates whether editing was detected by Sanger sequencing. OT1 corresponds to the GS1 locus, which has complete sequence identity to the GS5 target and was the only edited off-target site. Mismatched nucleotides at OT2-OT13 are shown in red. (b) Numbers of homozygous and heterozygous indels (left) and SNPs (right) identified from RNA-seq data for AK1, ZN, EK1, and E7. (c) Distribution of variants according to predicted coding consequence in the four cell lines. Data are mean ± SD from three independent biological replicates (n = 3). (d) PCR detection of the CBE4Max-SpRY and sgRNA-BSD plasmid sequences in the initial screening populations and the derived E2-E7 monoclonal lines. Plasmid DNA and untransfected cells served as positive and negative controls, respectively. Neither plasmid-derived sequence was detected in the established monoclonal lines.
